# Global, regional, and national burden of gallbladder and biliary tract cancer among adults aged 55 years and older, 2010–2021

**DOI:** 10.3389/fnut.2025.1561712

**Published:** 2025-07-01

**Authors:** Mingjuan Li, Jiaguang Li, Shuangjiang Li, Minmin Zhang, Shuang Li, Jiahui Zhao, Tao Gan, Min Wu, Shunwen Luo, Yunying Liang, Qiuyun Li, Guangdong Pan, Jianqing Yang, Guoqing Ouyang

**Affiliations:** ^1^Department of General Surgery, Liuzhou People's Hospital Affiliated to Guangxi Medical University, Liuzhou, China; ^2^Liuzhou Hepatobiliary and Pancreatic Diseases Precision Diagnosis Research Center of Engineering Technology, Liuzhou People's Hospital Affiliated to Guangxi Medical University, Liuzhou, China; ^3^Pathology Department, Liuzhou People's Hospital Affiliated to Guangxi Medical University, Liuzhou, China; ^4^Emergency Medicine Department, Liuzhou People's Hospital Affiliated to Guangxi Medical University, Liuzhou, China; ^5^Guangxi Clinical Key Specialty (Emergency Medicine Department), Liuzhou, China

**Keywords:** gallbladder and biliary tract cancer, GBD, incidence, death, DALYs, 55 years and older

## Abstract

**Introduction:**

Gallbladder and Biliary Tract Cancer (GBTC) accounts for a notable proportion of cancer cases worldwide. This study aims to assess the burden of GBTC among aged 55 years and older, enhancing our understanding of its trends and their relationship with Socio-Demographic Index (SDI) across regions and countries.

**Methods:**

We used the data from the Global Burden of Disease study from 2010 to 2021 to analyze GBTC incidence, death numbers, disability-adjusted life years (DALYs) and their respective rates for individuals aged 55 years and older. We also reported the GBTC percentage trend during this period.

**Results:**

The global incidence and death cases of GBTC changed significantly between 2010 and 2021. Compared to 2010, GBTC incidence and deaths increased by 35.9% and 29.7% individually in 2021. High-income Asia Pacific was the region with the highest age-standardized incidence, deaths, and DALYs in 2021, and China was the country with the largest number of incidence cases, deaths, and DALYs in 2021. Furthermore, the highest age-standardized incidence rate and age-standardized death rate occurred in Japan. Among individuals aged 55 years and older, the highest incident and death cases were observed in 70–74 age group, and females were more suffered from GBTC than males between 2010 and 2021.

**Conclusion:**

GBTC remains a substantial and growing global health burden, particularly among females and the aged from 2010 to 2021. The absolute number of GBTC cases continued to rise over the past decade in aged 55 years and older.

## 1 Introduction

Although gallbladder and biliary tract cancers (GBTC) are relatively rare globally, their prognosis remains poor, with a 5-year survival rate under 20% in most European countries (1). Recent years have witnessed an alarming rise in GBTC prevalence (2, 3), making it a significant health burden worldwide, especially in China (4). GBTC is categorized by anatomical location as intrahepatic cholangiocarcinoma, perihilar cholangiocarcinoma, distal cholangiocarcinoma, and gallbladder cancer (5). The prognosis for both gallbladder cancer and cholangiocarcinoma are generally poor, with median survival rate ranging from ~3–6 months in unresectable cases (4). Even after complete resection, the overall 5-year survival rate remains disappointingly low (6). The incidence and epidemiology of GBTC are complex and vary widely. Gallbladder cancer constitutes 80%−95% of biliary tract cancers (3, 7). Notably, according to our previous study geographic variations significantly influence the incidence of GBTC, with highest age-standardized incidence rates (ASIR) observed in high-income Asia Pacific and southern Latin America in 2017, particular in countries like Japan and Chile (7–9).

Simultaneously, the global population aging—driven by increasing life expectancy and population growth, particularly in China and India—is expected to intensify the burden of chronic diseases, including cancers. By 2050, it is projected that over 0.75 billion people aged sixty and older will reside in these two nations, comprising 38.5% of the world's sixty-plus population (10). Previous study showed that cancer incidence is notably higher among aging populations, especially those aged 55 years and older, who often face age-related functional declines and multi-system diseases (11, 12). In our global burden of diseases (GBD) 2017 study also showed that the incidence and deaths number of age above 55 groups was higher than age below 55 groups (9). Therefore, it is crucial to consider GBTC disease burden in this age group, as the impact on families and society could be substantial and help policy makers in implementing effective policies.

While several studies have estimated the GBTC burden, indicating GBTC is still a major public issue (9, 13), most studies have focused on general populations across all age groups and the past 30 years burden, with little emphasis on older adults, who are more susceptible to GBTC (14–16). Until now, no previous studies have specifically focused on the GBTC burden in individuals aged 55 and older, especially the trends of GBTC over the past decade. To address this gap, this study presents estimates of the number of cases and age-standardized rates (ASRs) for GBTC incidence, mortality, and disability-adjusted life years (DALYs) across 204 countries and territories from 2010 to 2021 for individuals aged 55 years and older (17). These estimates are stratified by age, sex, and the Socio-Demographic Index (SDI). To our knowledge, this is the first study to explore the relationship between trends in GBTC and SDI at both regional and national levels over this period and age groups.

## 2 Materials and methods

### 2.1 Overviews

The Global Burden of Disease (GBD) study was initiated to provide a comprehensive, publicly accessible resource for global disease burden metrics. Data were collected from 204 countries and territories, which were grouped into 21 regions. These countries were further classified into five SDI levels—high, high-middle, middle, low-middle, and low—using data spanning from 2010 to 2021. The Global Health Data Exchange (http://ghdx.healthdata.org/gbd-results-tool) provides detailed datasets on incidence, mortality, DALYs, Years of Life Lost (YLLs), and Years of Lived Disability (YLDs). The methodologies employed to derive these metrics have been thoroughly documented in previous research (17, 18). This study was conducted in compliance with the Guidelines for Accurate and Transparent Health Estimates Reporting (GATHER) (19).

### 2.2 Data sources

The GBD estimation process integrates multiple relevant data sources for each disease or injury, including censuses, household surveys, civil registration and vital statistics, disease registries, health service use, air pollution monitors, satellite imaging, disease notifications, and other sources (20). All cancers were coded as C23 through C24.9 based on the 11th revision of the *International Classification of Diseases* (ICD) were included in this study (20). Intrahepatic bile duct cancer (ICD-10 code C22.1) was excluded to avoid overlap with others research.

### 2.3 Statistical analysis

In our study, we utilized the ASIR, age-standardized mortality rate (ASMR) and age-standardized disability-adjusted life year (ASDR) to quantify the trends in the global, regions and countries GBTC burden. DALYs were employed to evaluate GBTC's societal impact and inform public health strategies. IHME calculated the percentage change in each metric between 2010 and 2021 by subtracting the 2010 value from the 2021 value and then dividing by the 2010 baseline. Specifically, the trend of incidence, death, and DALYs of were assessed in this way to quantify (18). All data were drawn from the GBD database. All data statistics were analyzed using the R statistical software program (version 3.6.3; R Foundation for Statistical Computing). *P* values < 0.05 were considered statistically significant.

### 2.4 Uncertainty analysis

We report the 95% uncertainty intervals (UIs) for all estimates. The 95% UI represents the range from the 2.5th to the 97.5th percentile of the calculated parameters based on the disease data.

### 2.5 Mortality estimates

First, by using data sources that reported accurate incidence and mortality rates in the same year, the mortality-to-incidence ratio (MIR) for GBTC was estimated using a linear mixed-effects model with the Healthcare Access and Quality (HAQ) Index, age, and sex as covariates. Second, the MIR was combined with cancer registry incidence data to estimate mortality. Third, the estimated mortality for GBTC was combined with observed mortality (from vital registrations) and entered into the Cause of Death Ensemble Model (CODEm). CODEm was then used to predict GBTC mortality for all locations, years, sexes, and ages. Finally, the CoD Correct algorithm was used to adjust the sum of predicted single-cause mortality (CODEm results) to be consistent with the all-cause mortality estimation (9).

### 2.6 Socio-demographic index

The SDI was calculated based on the total fertility rate for those aged under 25 years, the average education level, and the per capita income of the female population aged over 15 years. It serves as a composite indicator to measure a country or region's per capita income, mean education level, and total fertility rate.

## 3 Results

### 3.1 Global burden of gallbladder and biliary tract cancer

**Figure 1:**
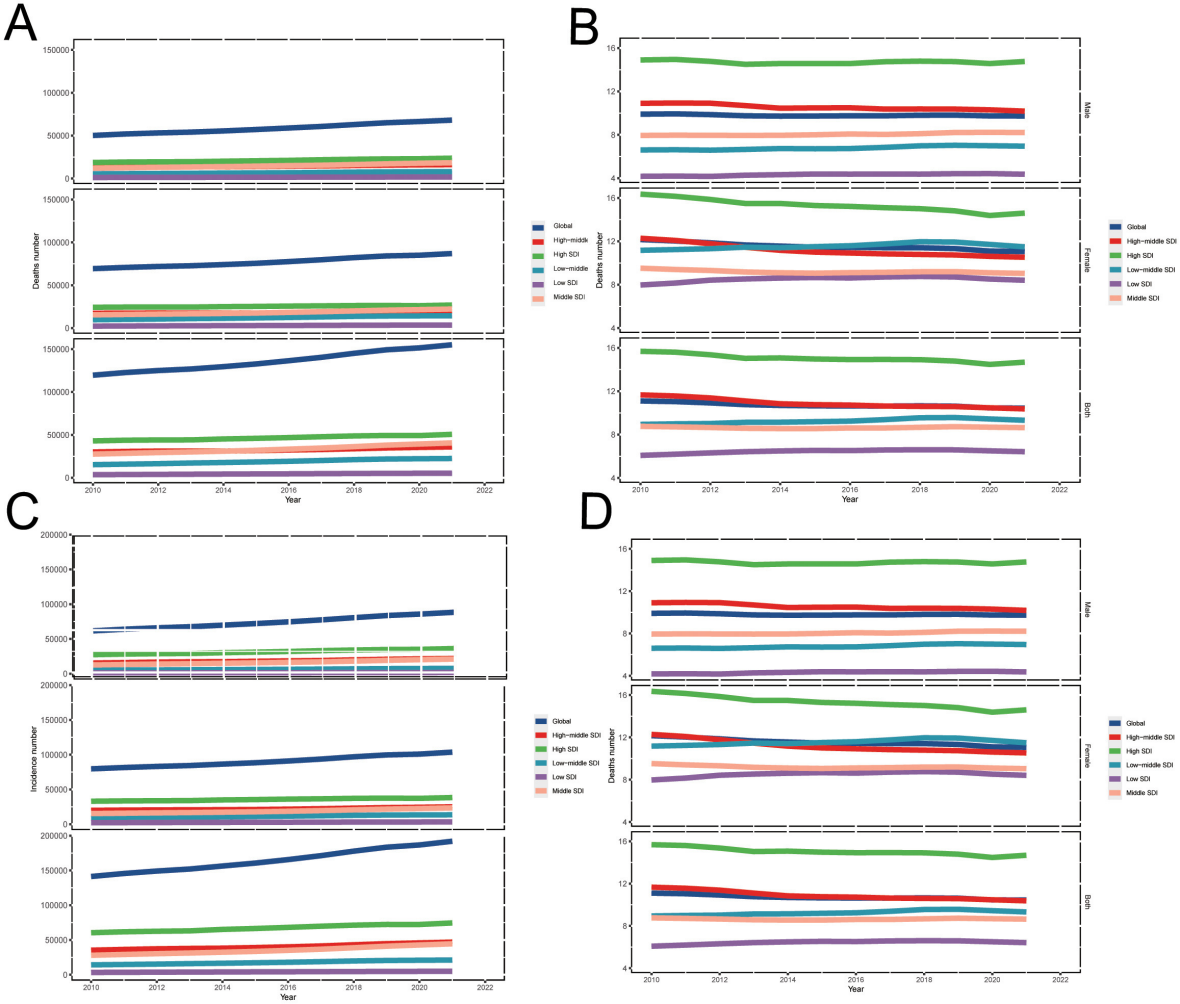
Trends in GBTC burden of **(A)** deaths cases, **(B)** ASMR, **(C)** incident cases, and **(D)** ASIR across global and 5 SDI regions, from 2010 to 2021 for aged 55 years and older. GBTC, gallbladder and biliary tract cancer; ASMR, age-standardized death rate; ASIR, age-standardized incidence rate; SDI, Socio-Demographic Index.

Globally in 2021, the number of GBTC deaths was 155,077 (95% UI: 129,703 to 174,512) in aged ≥55 group (Table 1, Figure 1A), while in 2010, the number of deaths cases were 119,606 (95% UI: 102,399 to 132,423). The ASMR decreased from 11.1 (95% UI: 9.5 to 12.3) per 100,000 population in 2010 to 10.4 (95% UI: 8.7 to 11.7) per 100,000 population in 2021 in aged 55 years and older group (Figure 1B, Table 1). Globally, the number of GBTC incidence cases was 141,299 (95% UI: 121,392 to 154,339) in 2010, which increased to 192,028 (95% UI: 163,007 to 216,508) in 2021 (Table 1, Figure 1C). Over the past decade, there has been a slight decrease in the overall ASIR for GBTC among individuals aged 55 years and older worldwide. In 2010, the ASIR was 13.1 per 100,000 population, whereas in 2021, it decreased to 12.9 per 100,000 population, representing a 1.4% reduction in incidence (Figure 1D, Table 1).

**Table 1:** Deaths, incidence cases, and disability-adjusted life years (DALYs) for GBTC in 2021 and percentage change in age-standardized rates (ASRs) per 100,000 population from 2010 to 2021 by global, 5 SDI, 21 regions burden for the population aged 55 years and older of disease.

	**Incidence (95% uncertainty interval)**			**Deaths (95% uncertainty interval)**			**DALYs (95% uncertainty interval)**		
**Location name**	**Number_2021**	**ASIR per 100,000 population in 2021**	**Percentage change in ASIRs per 100,000 population**	**Number_2021**	**ASMR per 100,000 population in 2021**	**Percentage change in ASMRs per 100,000 population**	**Number_2021**	**ASDR per 100,000 population in 2021**	**Percentage change in ASDRs per 100,000 population**
Global	192028 (163007 to 216508)	12.9 (11 to 14.6)	−1.4 (−8.1 to 5)	155077 (129703 to 174512)	10.4 (8.7 to 11.7)	−6 (−12.4 to 0.1)	2999189 (2489403 to 3445819)	201.8 (167.5 to 231.9)	−7.1 (−14.2 to −0.8)
Female	103666 (85095 to 118251)	13.2 (10.8 to 15)	−5.6 (−12.5 to 1.4)	86997 (70330 to 101030)	11.1 (8.9 to 12.8)	−9 (−15.6 to −2.3)	1656130 (1316137 to 1924050)	210.6 (167.3 to 244.6)	−9.3 (−16.2 to −2.1)
Male	88362 (68721 to 101666)	12.6 (9.8 to 14.5)	3.9 (−5.9 to 14.5)	68080 (51662 to 79621)	9.7 (7.4 to 11.4)	−1.7 (−11 to 7.5)	1343059 (997441 to 1585590)	192 (142.6 to 226.7)	−4.3 (−14.2 to 5.6)
Middle SDI	44488 (36183 to 57850)	9.5 (7.7 to 12.3)	6.6 (−5.4 to 18.5)	40619 (33079 to 51794)	8.6 (7 to 11)	−1.3 (−11.9 to 8.8)	856494 (693810 to 1087204)	182.3 (147.7 to 231.4)	−2 (−12.8 to 8)
Low SDI	4910 (3366 to 6010)	6 (4.1 to 7.3)	6.4 (−5.4 to 19.6)	5271 (3617 to 6428)	6.4 (4.4 to 7.8)	5.6 (−6 to 18.7)	117969 (81116 to 144980)	143.8 (98.9 to 176.7)	4.2 (−8 to 17.6)
Low-middle SDI	21123 (16675 to 26296)	8.8 (6.9 to 10.9)	5.5 (−3.9 to 15.1)	22457 (17706 to 27976)	9.3 (7.3 to 11.6)	4.2 (−4.9 to 13.5)	495546 (384644 to 614392)	205.6 (159.5 to 254.8)	3.6 (−5.9 to 13.3)
High SDI	74481 (64593 to 81773)	21.6 (18.7 to 23.7)	−2 (−6.4 to 2.1)	50641 (43444 to 55944)	14.7 (12.6 to 16.2)	−6.4 (−10.7 to −2.4)	821871 (720349 to 899679)	238.2 (208.8 to 260.8)	−11.6 (−15.5 to −7.5)
High-middle SDI	46880 (36383 to 53931)	13.5 (10.5 to 15.6)	−1.2 (−12.2 to 10.5)	35957 (28256 to 41125)	10.4 (8.2 to 11.9)	−11 (−19.9 to −1.3)	704746 (547705 to 806560)	203.3 (158 to 232.7)	−11.9 (−21.2 to −1.6)
East Asia	46000 (32202 to 59042)	11.7 (8.2 to 15.1)	7.8 (−12.2 to 32.4)	35027 (24815 to 45315)	8.9 (6.3 to 11.6)	−6.7 (−23.9 to 13.8)	708497 (507185 to 918246)	180.7 (129.3 to 234.2)	−9.1 (−26.6 to 11.5)
Southeast Asia	10092 (7316 to 12808)	8.8 (6.4 to 11.2)	7.5 (−13.6 to 34.7)	9689 (7099 to 12366)	8.5 (6.2 to 10.8)	3 (−16.5 to 27.9)	208887 (152480 to 266553)	182.3 (133.1 to 232.7)	2.3 (−16.9 to 27.4)
Oceania	28 (19 to 35)	2.3 (1.5 to 2.9)	−8.4 (−23.7 to 6.5)	29 (20 to 37)	2.4 (1.6 to 3)	−9.2 (−24.6 to 6.1)	698 (473 to 898)	56.6 (38.3 to 72.7)	−7.8 (−23.4 to 8.8)
Central Asia	552 (490 to 624)	3.8 (3.4 to 4.3)	3.1 (−9 to 18)	572 (507 to 647)	3.9 (3.5 to 4.4)	0.1 (−11.7 to 14.7)	13041 (11522 to 14798)	89.6 (79.2 to 101.7)	4.8 (−8.2 to 20.2)
Central Europe	6003 (5446 to 6563)	16.2 (14.7 to 17.7)	−7 (−13.7 to −0.4)	5668 (5171 to 6172)	15.3 (14 to 16.7)	−10 (−16.1 to −4)	107815 (98927 to 117558)	291.2 (267.2 to 317.5)	−11.7 (−18 to −5.5)
Eastern Europe	5907 (5468 to 6376)	9.5 (8.8 to 10.3)	9.6 (2.5 to 17.7)	4362 (4018 to 4715)	7 (6.5 to 7.6)	−3.2 (−10 to 4.5)	88709 (81798 to 96141)	142.9 (131.8 to 154.9)	−4.3 (−11.2 to 4)
High-income Asia Pacific	35718 (29693 to 40139)	50.7 (42.1 to 56.9)	6 (−1.2 to 12.3)	27236 (22411 to 30617)	38.6 (31.8 to 43.4)	1.3 (−4.4 to 6.4)	397408 (340285 to 441791)	563.7 (482.7 to 626.6)	−8.7 (−14.1 to −3.8)
Australasia	1675 (1447 to 1815)	19 (16.4 to 20.5)	−10.8 (−17.2 to −4.4)	592 (516 to 638)	6.7 (5.8 to 7.2)	−8.7 (−13.2 to −4.4)	10325 (9250 to 11061)	116.9 (104.7 to 125.2)	−9.9 (−14.5 to −5.7)
Western Europe	24022 (21051 to 25922)	16.1 (14.1 to 17.4)	−4.7 (−8.1 to −1.5)	15507 (13491 to 16822)	10.4 (9 to 11.3)	−9.5 (−12.4 to −6.7)	258346 (231838 to 276474)	173.2 (155.5 to 185.4)	−12.6 (−15.2 to −10)
Southern Latin America	3917 (3553 to 4172)	26.6 (24.1 to 28.4)	−19.4 (−23.5 to −14.5)	3618 (3302 to 3843)	24.6 (22.4 to 26.1)	−23.7 (−27.5 to −18.9)	71856 (66744 to 76018)	488.3 (453.5 to 516.6)	−23.8 (−27.7 to −19.1)
High-income North America	12854 (11523 to 13601)	11.4 (10.2 to 12.1)	−5.7 (−8.1 to −3.2)	5611 (4999 to 5948)	5 (4.4 to 5.3)	−8.3 (−10.5 to −6.1)	106033 (97680 to 111349)	94.2 (86.8 to 98.9)	−6.6 (−8.8 to −4.4)
Caribbean	460 (402 to 524)	5 (4.3 to 5.7)	−12.1 (−21.6 to −3.8)	460 (402 to 528)	5 (4.3 to 5.7)	−13.7 (−23.2 to −5.6)	9494 (8240 to 10981)	102.5 (89 to 118.6)	−12.4 (−22.1 to −3.6)
Andean Latin America	2002 (1529 to 2607)	20.2 (15.4 to 26.3)	−3.9 (−21.8 to 18.9)	2059 (1574 to 2644)	20.8 (15.9 to 26.7)	−7.8 (−24.8 to 13.7)	42241 (31942 to 55514)	426.4 (322.4 to 560.4)	−6.9 (−24.7 to 16)
Central Latin America	4504 (4012 to 4993)	10.5 (9.4 to 11.7)	−8.8 (−17.9 to 0.6)	4602 (4109 to 5090)	10.8 (9.6 to 11.9)	−11.6 (−20.6 to −3)	96533 (86102 to 107719)	225.7 (201.3 to 251.9)	−9.6 (−19.2 to 0.1)
Tropical Latin America	4894 (4450 to 5158)	11 (10 to 11.6)	−1.3 (−4.6 to 2.1)	5078 (4593 to 5355)	11.5 (10.4 to 12.1)	−4.1 (−7.2 to −0.7)	105304 (97650 to 110140)	237.7 (220.4 to 248.6)	−2.9 (−6.5 to 0.6)
North Africa and Middle East	4766 (3524 to 5910)	6.3 (4.6 to 7.8)	−6.8 (−15.5 to 2.9)	4662 (3454 to 5781)	6.1 (4.5 to 7.6)	−11.1 (−19.2 to −2)	98775 (72369 to 120634)	129.6 (94.9 to 158.2)	−10.8 (−19.6 to −1.5)
South Asia	26892 (18705 to 31802)	10.8 (7.5 to 12.8)	11.8 (−1.4 to 25.1)	28423 (19697 to 33691)	11.4 (7.9 to 13.6)	9.9 (−2.6 to 22.7)	633426 (434141 to 751236)	255.1 (174.8 to 302.6)	8.9 (−3.7 to 22)
Central Sub-Saharan Africa	128 (86 to 180)	1.4 (1 to 2)	1.2 (−18 to 23.2)	136 (92 to 193)	1.5 (1 to 2.1)	0.1 (−18.3 to 22.7)	3233 (2185 to 4580)	35.8 (24.2 to 50.8)	2 (−18.5 to 25.7)
Eastern Sub-Saharan Africa	1173 (830 to 1547)	4.3 (3.1 to 5.7)	1.6 (−9.9 to 13.4)	1271 (900 to 1681)	4.7 (3.3 to 6.2)	1.2 (−10.2 to 12.7)	28172 (19781 to 37345)	104.2 (73.2 to 138.1)	0.2 (−11.7 to 11.6)
Southern Sub-Saharan Africa	367 (258 to 432)	3.8 (2.7 to 4.4)	−3.5 (−16.5 to 10)	390 (274 to 457)	4 (2.8 to 4.7)	−5.4 (−18 to 7.7)	8594 (6052 to 10159)	88.3 (62.2 to 104.4)	−4.4 (−17.6 to 10)
Western Sub-Saharan Africa	77 (52 to 94)	0.2 (0.2 to 0.3)	7.9 (−12.2 to 26.8)	84 (57 to 103)	0.3 (0.2 to 0.3)	6.9 (−12.8 to 25.6)	1803 (1246 to 2237)	5.6 (3.9 to 7)	8.2 (−11.8 to 29.1)

ASIR represents age-standardized incidence rates.

ASMR represents age-standardized mortality rates.

ASDR represents age-standardized disability-adjusted life year rates.

Globally in 2010, GBTC DALYs cases accounted for 2,341,779 (95% UI: 1,970,210 to 2,623,883). By 2021, the number of DALYs cases had risen to 2,999,189 (95% UI: 2,489,403 to 3,445,819), representing a 28.1% increase (Table 1). The ASDR of GBTC in aged 55 years and older were 217.0 (95% UI: 182.8 to 243.5) per 100,000 population in 2010 and, 201.8 (95% UI: 167.5 to 231.9) per 100,000 population in 2021, a 7.1% (95% UI: −14.2% to −0.8%) decrease from 2010 to 2021 (Table 1).

In the worldwide, the percentage of ASMR [−6.0% (95% UI: −12.4% to 0.1%)] and ASIR [−1.4% (95% UI: −8.1% to 5.0%)] were both showed a declined trends from 2010 to 2021 (Table 1). However, absolute numbers rose from 2010 to 2021, namely, the number of deaths were increase 29.7% (95% UI: 20.8% to 38.0%) and incidence were 35.9% (95% UI: 26.7% to 44.9%; Table 1).

### 3.2 Regional burden of gallbladder and biliary tract cancer

**Figure 2:**
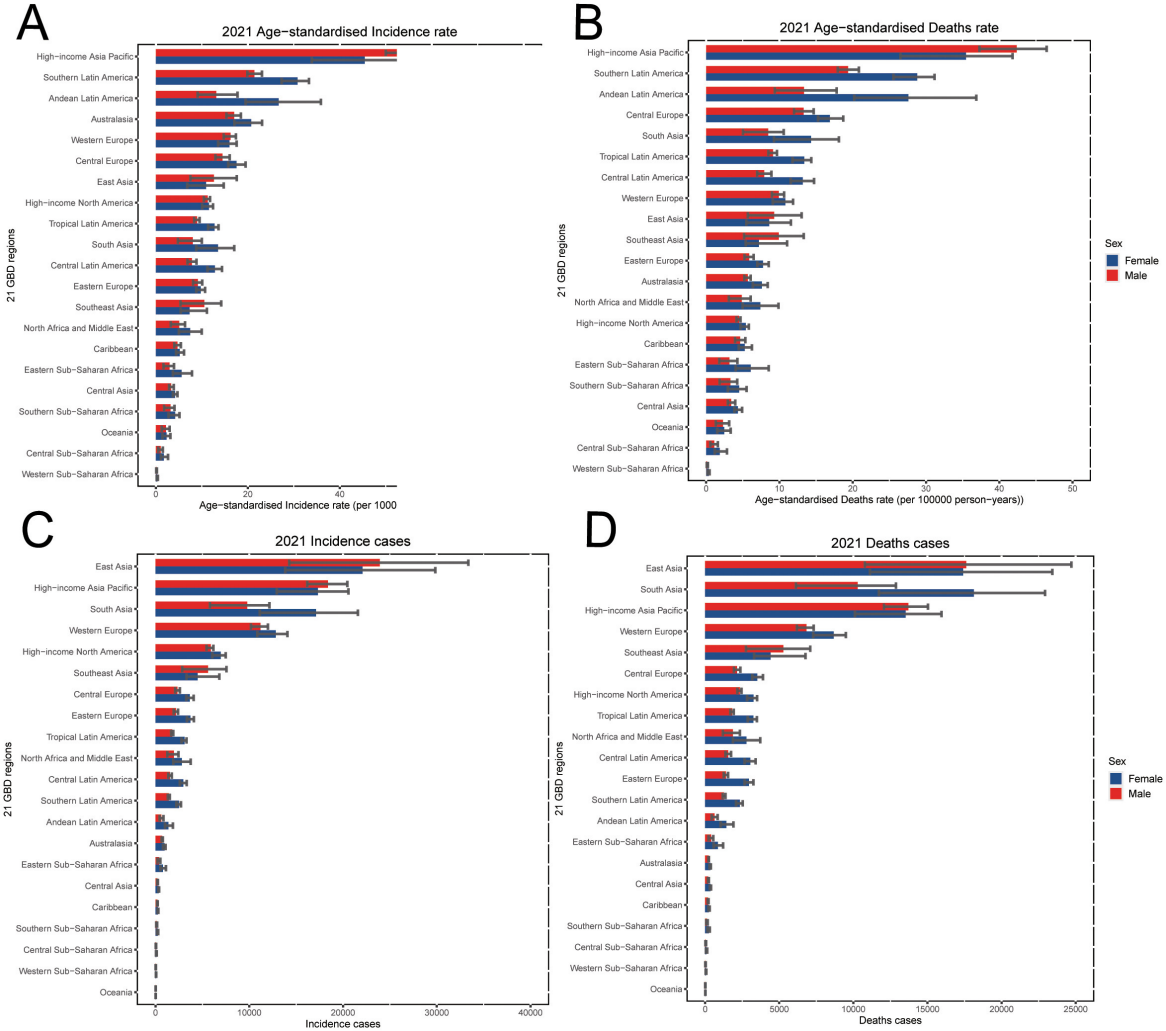
In 2021, the GBTC burden of 21 regions for individuals aged 55 years and older, including **(A)** ASIR, **(B)** ASMR, **(C)** incidence cases and **(D)** death cases by sexes. GBTC, gallbladder and biliary tract cancer; ASIR, age-standardized incidence rate; ASMR, age-standardized death rate.

In 2021, the highest ASIR for individuals aged 55 years and older was observed in the High-income Asia Pacific region for both sexes, with a rate of 50.7 per 100,000 population (95% UI: 42.1 to 56.9), followed by Southern Latin America [26.6 per 100,000 (95% UI: 24.1 to 28.4)], and Andean Latin America [20.2 per 100,000 (95% UI: 15.4 to 26.3), Figure 2A, Table 1]. In the same time, East Asia has the largest incidence number than the other regions (Figure 2C). In contrast, the lowest ASIRs were recorded in Western Sub-Saharan Africa [0.2 per 100,000 (95% UI: 0.2 to 0.3)], Central Sub-Saharan Africa [1.4 per 100,000 (95% UI: 1.0 to 2.0)], and Oceania [2.3 per 100,000 (95% UI: 1.5 to 2.9), Figure 2A, Table 1].

At the regional level, the highest ASMR in 2021 was also found in the High-income Asia Pacific region [38.6 per 100,000 population (95% UI: 31.8 to 43.4)], followed by Southern Latin America [24.6 per 100,000 (95% UI: 22.4 to 26.1)], and Andean Latin America [20.8 per 100,000 (95% UI: 15.9 to 26.7), Figure 2B, Table 1]. And the East Asia had the highest death number than the other regions (Figure 2D). Conversely, the lowest ASMRs of GBTC for individuals aged 55 years and older were found in Western Sub-Saharan Africa [0.3 per 100,000 (95% UI: 0.2 to 0.3)], Central Sub-Saharan Africa [1.5 per 100,000 (95% UI: 1.0 to 2.1)], and Oceania at 2.4 per 100,000 (95% UI: 1.6 to 3.0; Figure 2B, Table 1).

Regarding the ASDR for GBTC in 2021 among those aged 55 years and older, the lowest burdens were observed in Western Sub-Saharan Africa [5.6 per 100,000 (95% UI: 3.9 to 7.0)], Central Sub-Saharan Africa [35.8 per 100,000 (95% UI: 24.2 to 50.8)], and Oceania [56.6 per 100,000 (95% UI: 38.3 to 72.7), Supplementary Figure 1]. In contrast, the highest ASDRs were found in the High-income Asia Pacific [563·7 per 100,000 (95% UI: 482.7 to 626.6)], Southern Latin America [488.3 per 100,000 (95% UI: 453.5 to 516.6)], and Andean Latin America [426.4 per 100,000 (95% UI: 322.4 to 560.4)] regions among the 21 GBD regions for the population aged 55 years and older (Supplementary Figure 1).

Despite regional variation in incidence numbers, all 21 GBD regions experienced increasing trends between 2010 and 2021. Southeast Asia [66.4% (95% UI: 33.9% to 108.6%)], South Asia [60.5% (95% UI: 41.6% to 79.6%)] and East Asia [58.0% (95% UI: 28.6% to 94.0%)] showed the largest increasing trends of incidence number during this period (Supplementary Table 1). South Asia [11.8% (95% UI: −1.4% to 25.1%)], Eastern Europe [9.6% (95% UI: 2.5% to 17.7%)] and Western Sub-Saharan Africa [7.9% (95% UI: −12.2% to 26.8%)] were top three increased in the percentage of ASIR from 2010 to 2021 (Table 1). Meanwhile, Southern Lartin America [−19.4% (95% UI: −23.5% to −14.5%)], Caribbean [−12.1% (95% UI: −21·6% to −3·8%)] and Australasia [−10.8% (95% UI: −17.2% to −4.4%)] were top three decreased in ASIR (Table 1). The regional changes in death counts did not always parallel incidence. Southeast Asia [59.5% (95% UI: 29.3% to 98.1%)] saw the greatest increase in deaths, whereas Southern Latin America had the largest decrease [−2.6% (95% UI: −7.4% to 3.5%), Supplementary Table 1]. For ASMR between 2010 and 2021, South Asia and Southern Latin America showed the largest increased and decrease trend, respectively (Table 1).

### 3.3 204 countries and territories burden of gallbladder and biliary tract cancer

**Figure 3:**
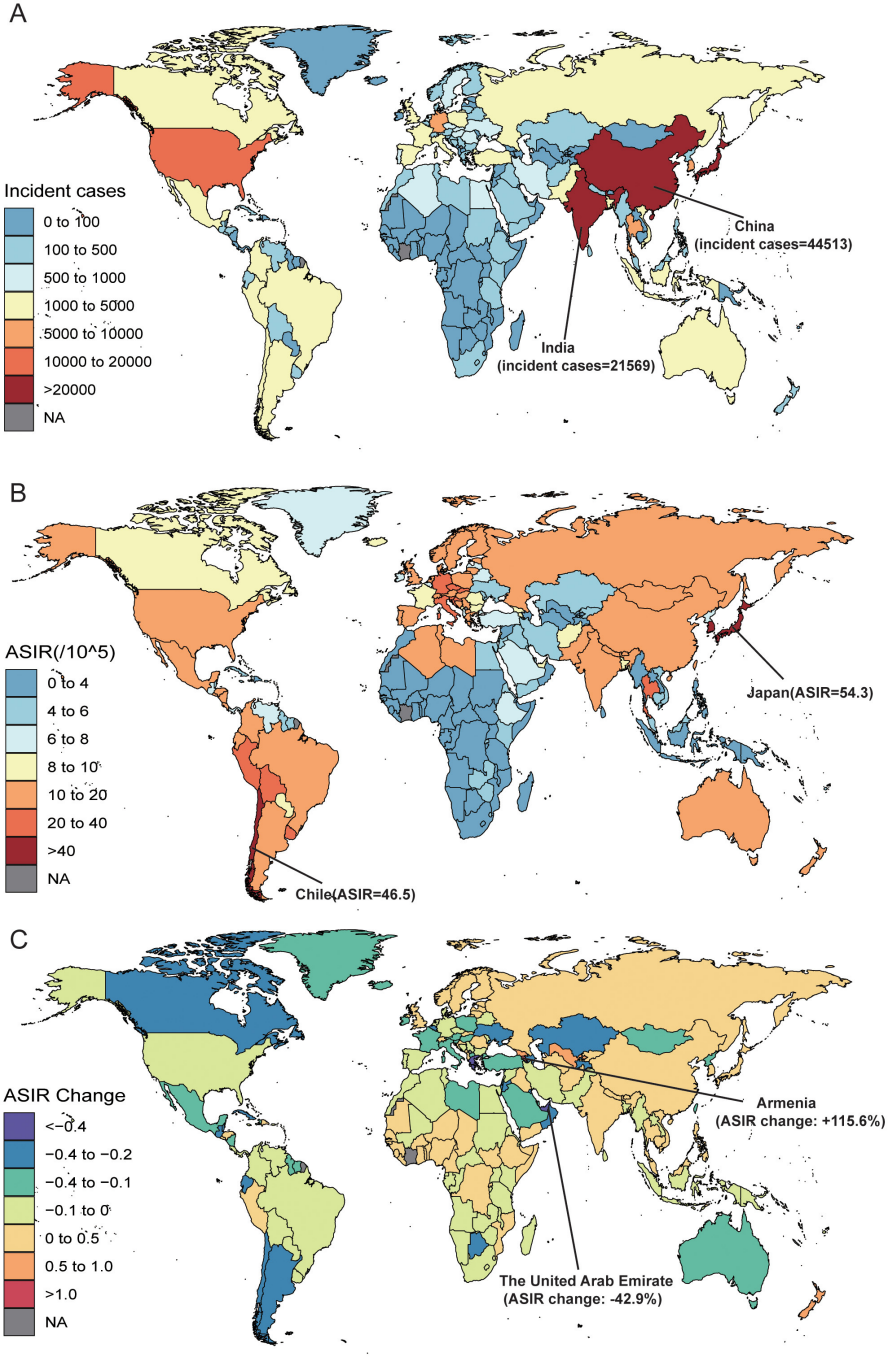
The Gallbladder and Biliary Tract Cancer (GBTC) burden of 204 countries and territories, for aged 55 years and older, including **(A)** the incidence cases of GBTC in 2021, **(B)** the age-standardized incidence rate (ASIR) of GBTC in 2021, and **(C)** from 2010 to 2021, the annual percent change of ASIR. GBTC indicates gallbladder and biliary tract cancer; ASIR, age-standardized incidence rate.

At the national and territories level in 2021, China (44,513; 95% UI: 30,655 to 57,373), Japan (28,358; 95% UI: 22,977 to 31,518) and India (21,569; 95% UI: 14,886 to 25,381) had the highest number of incident cases, and the highest number of deaths were China (33,806; 95% UI: 23,681 to 43,755), India (22,728; 95% UI: 15,624 to 26,748) and Japan (21,786; 95% UI: 17,687 to 24,178; Figures 3A, B, Supplementary Tables 2-S1, S2). In 2021, Japan suffered the highest ASIR (54.3 per 100,000; 95% UI: 44.0 to 60.4), ASMR (41.7 per 100,000; 95% UI: 33.9 to 46.3) (Figures 3A, B; Supplementary Figure 3, Tables 2-S1, S2). Whereas, Gambia, the Tokelau Islands, and Niue suffer the lowest burden in 2021. From 2010 to 2021, percentage changes in ASIR and ASMR varied across all countries and territories. Armenia showed the largest increase in ASIR (115.6%; 95% UI: 72.6% to 168.5%) and ASMR (110.2%; 95% UI: 68.3% to 161.2%), followed by Georgia and Uzbekistan (Figure 3C; Supplementary Figure 4, Tables 2-S1, S2). In contrast, United Arab Emirates experienced the most significant decreases in ASIR (−42.9%; 95% UI: −59.0% to 16.1%) and ASMR (−45.6%; 95% UI: −40.9% to −20.3%; Supplementary Tables 2-S1, S2).

In 2021, China [683,908 (95% UI: 482,234 to 886,803)] had the highest number of DALYs related to the burden of GBTC among individuals aged ≥55, followed by India and Japan (Supplementary Figure 5, Table 2-S3). The lowest number of DALYs were observed in Gambia, the Tokelau Islands, and Niue (Supplementary Table 2-S3). The highest ASDR for GBTC aged ≥ 55 in 2021 was recorded in Chile [805.4 per 100,000 (95% UI: 736.1 to 872.7)], followed by Thailand and Bolivia (Supplementary Figure 6, Table 2-S3). Conversely, the lowest ASDRs were recorded in Gambia (approaching 0.0), the Tokelau Islands and Niue (Supplementary Table 2-S3). Most countries and territories showed increasing trends in ASDR among individuals aged ≥55 from 2010 to 2021. From 2010 to 2021 the largest increases in ASDR were observed in Armenia [114.8% (95% UI: 71.3% to 168.0%)], followed by Georgia and Uzbekistan (Supplementary Figure 7, Table 2-S3). The largest decreases in ASDR were found in United Arab Emirates, Greece, and San Marino (Supplementary Table 2-S3).

### 3.4 The burden of gallbladder and biliary tract cancer between regions, countries and SDI

**Figure 4:**
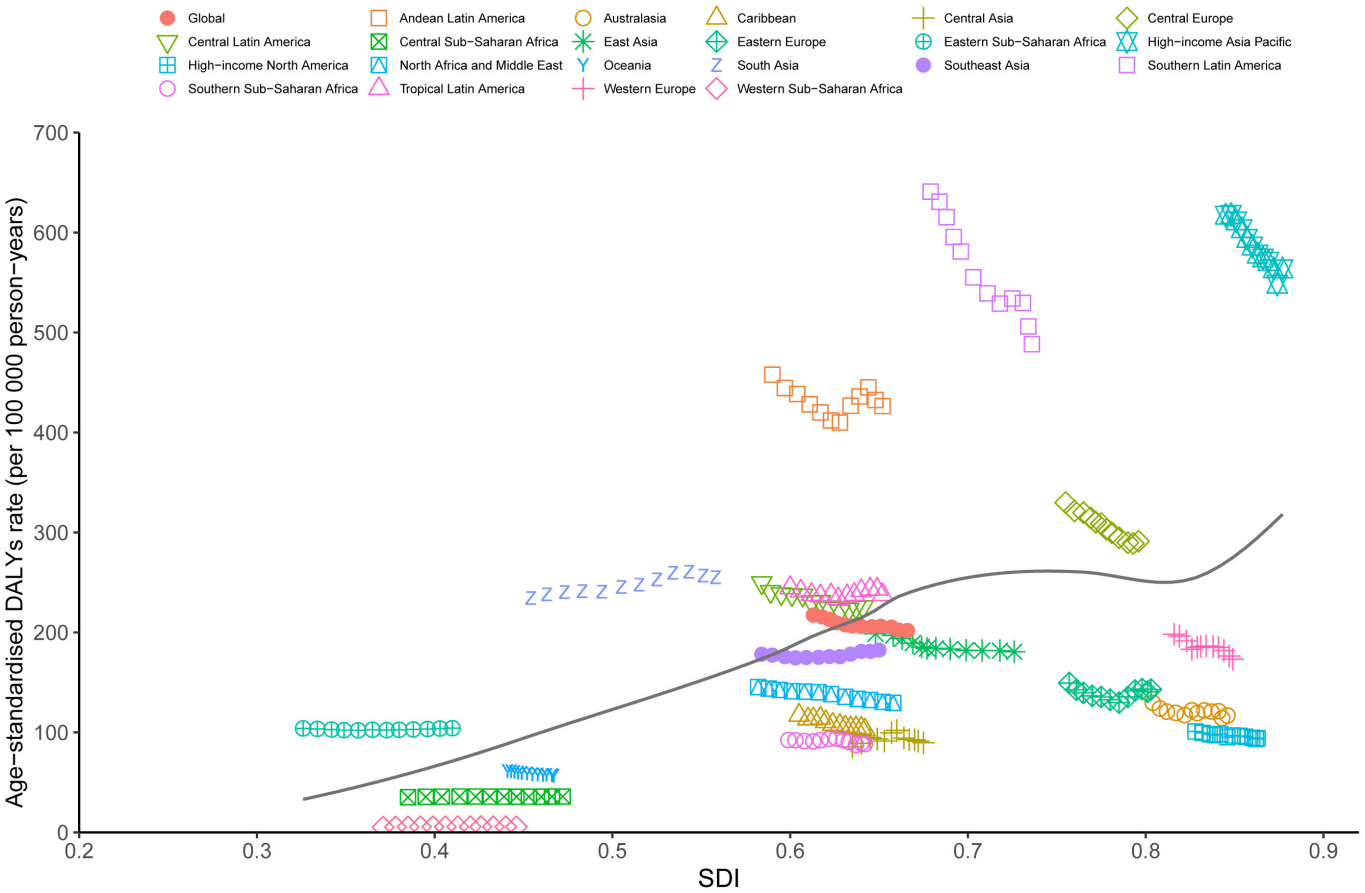
Trends in age-standardized DALYs rate (ASDR) for GBTC across 21 regions burden of disease is illustrated by SDI, from 2010 to 2021 for aged 55 years and older. The black line indicates expected values. GBTC, gallbladder and biliary tract cancer; ASDR, age-standardized disability-adjusted life years rate; SDI, Socio-Demographic Index.

Our results showed that the ASDR of GBTC aged ≥55 was positively correlated with the SDI at the regional level from 2010 to 2021 (Figure 4). The lowest ASDR was associated with an SDI value of ~0.371. Notably, regions such as High-Income Asia Pacific, Southern Latin America, Andean Latin America, and South Asia exhibited higher-than-expected ASDR levels, while areas including Eastern Europe, High-Income North America, Central Asia, and Southern Sub-Saharan Africa consistently showed lower-than-expected ASDR values (Figure 4). A similar trend was observed for the relationship between ASMR and ASIR with SDI (Supplementary Figures 8A, B).

**Figure 5:**
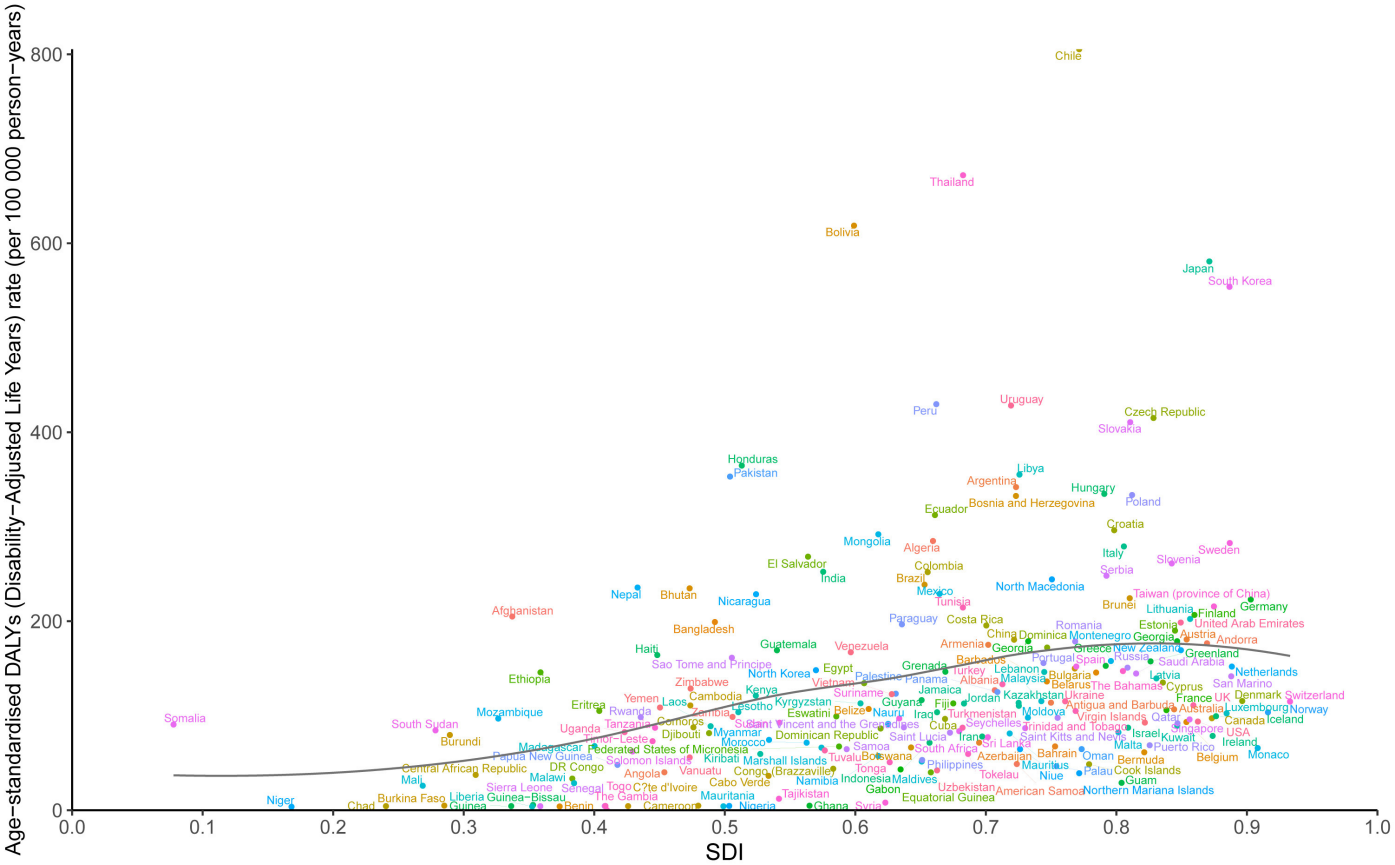
Trends in age-standardized DALYs rate (ASDR) for GBTC across 204 countries and territories burden of disease is illustrated by SDI, from 2010 to 2021 for aged 55 years and older. The black line indicates expected values. GBTC, gallbladder and biliary tract cancer; ASDR, age-standardized disability-adjusted life years rate; SDI, Socio-Demographic Index.

The analysis of DALYs for GBTC across 204 countries from 2010 to 2021 also revealed a general positive association between DALYs and SDI (Figure 5). The lowest ASDR among these countries was observed at an SDI value of around 0.410. Countries such as Chile, Thailand, Bolivia, Japan, and South Korea displayed significantly higher DALY rates than expected based on their SDI levels. Conversely, nations including Gambia, Niger, Liberia, Benin, and Mauritania reported markedly lower rates than anticipated (Figure 5). Additionally, ASIR and ASMR followed trends similar to ASDR from 2010 to 2021 (Supplementary Figures 9A, B).

### 3.5 The burden of gallbladder and biliary tract cancer between age and sex pattern

**Figure 6:**
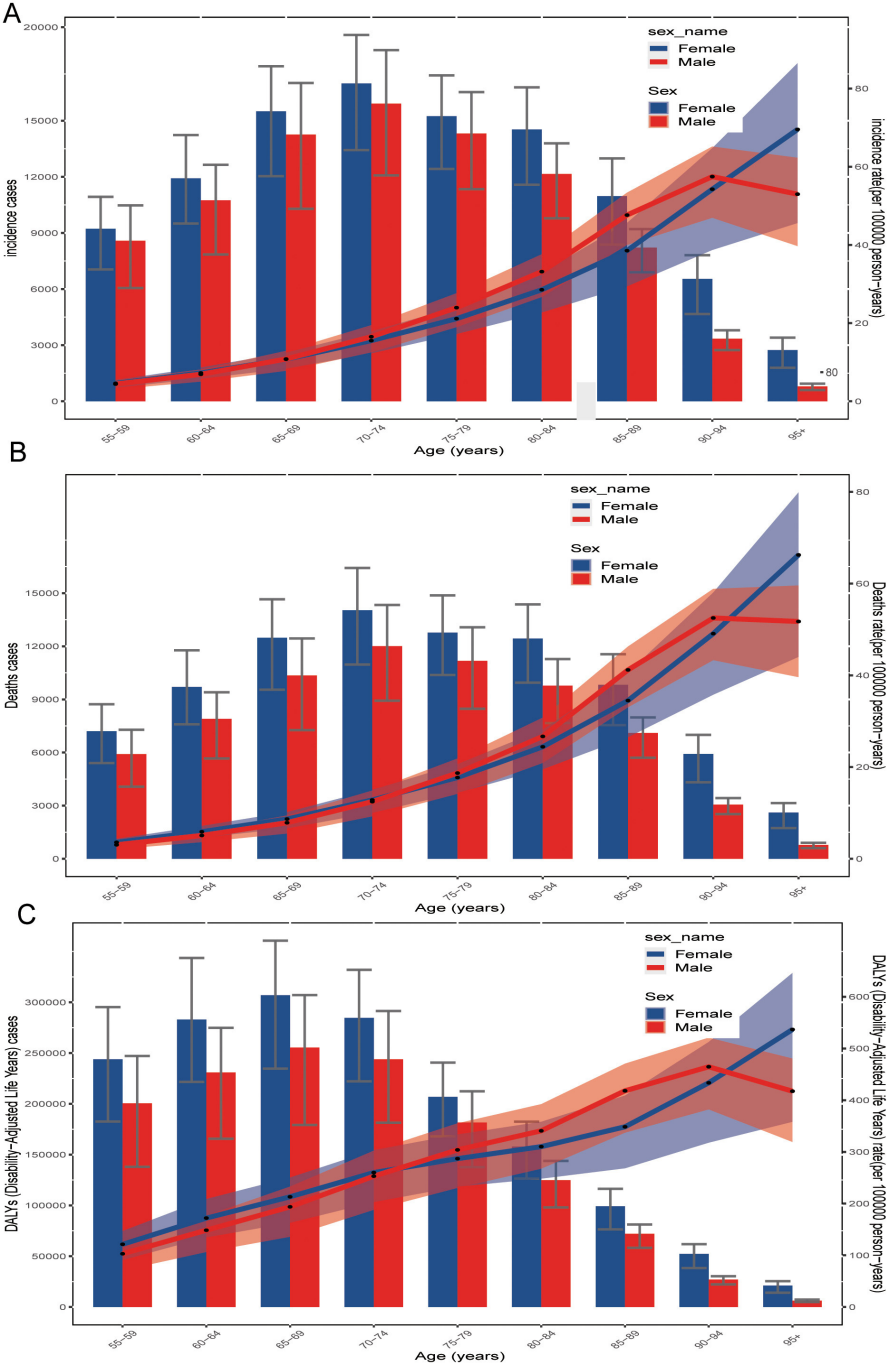
Age-specific numbers of **(A)** incidence cases, **(B)** death cases, and **(C)** disability-adjusted life years (DALYs) from GBTC in 2021 both are illustrated by age and sex, which aged 55 years and older. GBTC, gallbladder and biliary tract cancer; DALYs, disability-adjusted life years.

In 2021, the GBTC incidence, death cases, and DALYs varied by sex and age. The number of GBTC cases showed an increasing trend with age in both males and females, peaking in the 70–74 age group, followed by a slight annual decline (Figure 6A). Females consistently exhibiting a higher burden of incidence and death burden than males. Additionally, the incidence rate of GBTC generally increased with age, reaching its highest point in the 90–94 age group (Figure 6A). After this peak, the incidence rate declined rapidly in males but continued upward in females. This same trend was also observed in death cases rates (Figure 6B).

The pattern of DALYs differed somewhat. GBTC DALYs increased steadily with age, peaking in the 65–69 age group for both sexes, and then gradually declining in older age groups (Figure 6C). The DALY rate, like the incidence rate, generally increased with age, peaking in the 90–94 age group. After this peak, the DALY rate declined rapidly in males but continued to rise in females (Figure 6C).

### 3.6 Risk factors

GBD estimates identify elevated body-mass index (BMI) as the only major risk factor driving GBTC-related deaths and DALYs, and they demonstrate that GBTC burden varies markedly by age, sex, and region (Supplementary Figure 10). In our analysis of adults aged ≥55 years, the contribution of high BMI to both mortality and DALYs was particularly pronounced among those aged 55–59. In 2021, globally, 11.6% of GBTC deaths (10.3% in males; 12.6% in females) and 12.0% of DALYs (10.7% in males; 13.1% in females) were attributable to elevated BMI (Supplementary Figures 10A, B). Across the five SDI regions, the high-middle SDI category exhibited the highest BMI-attributable mortality proportion–14.1% overall (12.4% in males; 15.6% in females)—whereas the low SDI region showed the lowest proportion at 5.8% (4.9% in males; 6.3% in females; Supplementary Figures 10C, D).

## 4 Discussion

GBTC is relatively uncommon compared to other cancers, but recent evidence indicates increasing increase in incidence, mortality, and DALYs. Our study showed the most up-to-date and comprehensive data on GBTC aged 55 years and older from 2010 to 2021, revealing a substantial and rising global health burden, particularly among women and older adults. This finding underscores the need for focused prevention and treatment strategies for some specific groups. Additionally, our study emphasizes emphasize the critical role of improving early diagnosis rates to boost the chances of successful surgical interventions.

We found that the number of incidence cases and death of GBTC aged 55 years and older increased globally from 2010 to 2021, Whereas the ASR of GBTC for this age group were decreased during the past 10 years, consistent with previous studies (9, 21). A recent study predicted that global ASR of GBTC incidence and mortality would continue to decline from 2020 to 2030 (21). Nevertheless, researchers note that despite these ASR reductions, the absolute number of new cases and deaths may rise slightly in certain regions in certain regions (22). These global disparities in ASIR and ASMR stem from various complex factors, including local dietary habits, socioeconomic inequalities, environmental risk factors, genetic predisposition, and the prevalence of gallstone disease. Moreover, limited access to diagnostic tools and inconsistent data quality, especially in low- and lower-middle-income countries, significantly influence these trends (9, 13, 23, 24).

Several researchers have reported substantial geographic variation, with certain Asian regions showing notably high figures (9, 25, 26). Among the 21 GBD regions analyzed for GBTC burden in aged 55 years and older, our study found that the High-income Asia Pacific region (Japan and Korea) had the highest ASR in 2021. This elevated ASR may be linked to greater prevalence of clonorchiasis, hepatitis B, and obesity. Furthermore, liver fluke infections resulting from raw fish consumption in this area represent a key risk factor (27–29). Additional contributors to this region's high GBTC burden include unhealthy dietary patterns—such as excessive salt intake from salted fish, pickles, and cured meats—along with smoking and heavy alcohol use (9, 30, 31). Additionally, disparities in access to quality healthcare, particularly for early detection and treatment, may further amplify the burden of GBTC in different region. Socioeconomic development, dietary habits, population growth, and improvements in detection measures in these regions likely play significant roles in influencing this burden (32). Tumor development and progression are intricate processes driven by multiple carcinogenic factors. Consequently, the trends in GBTC morbidity and mortality are complex, underscoring the necessity for comprehensive, the need for tailored, region-specific interventions to improve healthcare access and prevention efforts. Implementing effective and feasible strategies for prevention, early detection, and treatment in these high-risk regions is essential to addressing the global GBTC burden.

In 2021, China had the highest number of GBTC incidence cases and deaths, largely due to its large population. A previous study reported that China had 254 million individuals aged 60 or older in 2019, this number is expected to rise to 402 million by 2040, which would account for approximately 28% of the total population (33). Studies also forecast a steady increase in GBTC incidence and mortality in China from 2020 to 2044 (22), driven by an aging population and demographic shifts that are expected to elevate age-standardized cancer rates (22, 25, 34). Additional factors, such as improved diagnostic, a shift from grain-based to high-energy, high-fat Western diets, rising body mass index (BMI), and viral hepatitis, further contribute to China's increased GBTC burden (22, 34–38). Japan recorded the highest ASIR and the ASMR, potentially tied to a higher prevalence of biliary tree malformations, growing obesity rates, an aging populace, and advanced diagnostic technologies (9, 37, 39). From 2010 to 2021, Armenia and Georgia saw the largest increases in GBTC burden among individuals aged 55 years and older. This mirrors the trend observed from 1990 to 2017 in these countries, indicating that the rising burden has continued over the past 4 years. This persistence suggests a lack of effective strategies to control GBTC in these regions, emphasizing the urgent need for policymakers to prioritize targeted interventions and preventive efforts. For example, tailored strategies should reflect each country's unique circumstances, including cultural practices, available resources, healthcare systems, and cancer awareness levels.

We also observed a significant disparity in the burden of GBTC between males and females, with females experiencing a higher burden—a finding consistent with prior research despite varying age groups (26, 37, 40, 41). The gender disparity in GBTC incidence may partly attributed to genetic and hormonal factors, as females have elevated estrogen levels during reproductive years, potentially increasing their risk (42–44). A previous report found that from 2011 to 2019, younger individuals (aged 20–54) accounted for just 10.1% of biliary tract cancer cases, while those aged 55–84 comprised 89.9% (45). Another study also reported that the incidence of GBTC in older patients was nearly double that of younger patients (46–48). Therefore, our research focused on the population aged 55 years and older and found that the number of incidence cases and deaths showed a generally increasing trend with age. Both the GBD 2017 (9) and GBD 2021 studies demonstrated that individuals aged 65–79 had a higher incidence and mortality rates. Several studies have identified older age as a significant independent risk factor for poor outcomes in patients with GBTC (32, 49, 50). These findings suggest that GBTC poses a significant threat to the elderly population, particularly affecting quality of life in the later stages and influencing therapeutic strategies (48, 51). Therefore, developing gender- and age-specific strategies to address GBTC's unique risk factors is essential. Screening programs for GBTC should be tailored to the specific needs of different age groups and genders, and future research should explore how genetics, hormones, lifestyle, and healthcare access shape these patterns. For example, screening women aged 70–74, launching dietary campaigns to discourage raw fish consumption, and promoting physical activity to reduce BMI and obesity could prove effective.

Although we previously reported the association between GBTC burden and SDI and HAQ, finding a negative correlation between GBTC burden and SDI across all age groups from 1990 to 2017 (9), and another study observed lower incidence of cholangiocarcinoma in high-income countries (52). However, we found a positive correlation between the GBTC burden and SDI in individuals aged 55 years and older from 2010 to 2021. This suggested that elderly GBTC patients in higher SDI regions or countries were face higher disease burden compared to lower SDI regions or countries. The discrepancy between the two studies may stem from differences in the age groups and study periods included. In our previous study, all age groups from 1990 to 2019 were considered, while the current study focuses on patients aged ≥55 from 2010 to 2021. Previous study showed that higher SDI regions are generally higher obesity rates, driven by lifestyle factors like high-calorie diets and reduced physical activity (53). In addition, Gallbladder and biliary diseases, particularly gallbladder cancer, are associated with higher BMI or obesity, which tend to have a higher prevalence in high-SDI regions (54). Therefore, greater attention should be focus on individuals aged 55 years and older in high-SDI regions. In addition, lower burden of GBTC in low SDI region may due to lower rate of obesity. However, although lower SDI regions have lower ASR, but we cannot overlook the burden of these regions, because there may be limited healthcare resources, lower educational attainment, and higher environmental risk exposures, which may lead to delays in GBTC diagnosis or undiagnosed and treatment (55).

Our study provided a high-quality analysis of global, regional, and national burden of GBTC from 2010 to 2021, focusing on individuals aged 55 and older. However, some limitations of this study warrant consideration. Firstly, this study is a secondary analysis of GBD data, and as with many GBD-based studies, the accuracy and reliability of the estimates depend heavily on the quality and quantity of the input data used for modeling. Secondly, the GBD data in this study covers only the period from 2010 to 2021, potentially introducing uncertainties and biases in the estimates. Furthermore, incorporating additional cancer databases could enhance the analysis's comprehensiveness and objectivity. Thirdly, this study does not investigate GBTC's economic impact or detail prevention strategies across different regions and countries, specifically, in low-SDI areas, underreporting, incomplete registries, and ICD coding changes may affect the results. So, significant variations may exist between low- and middle-income countries and high-income countries. Finally, this study focuses on individuals aged 55 years and older but lacks analysis of those under 55, who still represent a significant proportion of GBTC cases. Future research should include both age groups to assess age-related risk factors and further investigate gender differences in incidence.

In summary, our study examined the burden of GBTC among individuals aged 55 years and older, highlighting an escalating public health challenge with notable variations across age, sex, and geographic regions. Although ASRs of GBTC in this age group decreased from 2010 to 2021, the absolute number of GBTC cases continued to rise over the past decade. This underscores the need for a comprehensive and coordinated global response to this complex disease. Our findings may guide the development of strategies and interventions to prevent and manage GBTC risk factors effectively.

## Data Availability

The original contributions presented in the study are included in the article/Supplementary material, further inquiries can be directed to the corresponding authors.
