# Supplementary material for: Global, regional, and national burden of gallbladder and biliary tract cancer among adults aged 55 years and older, 2010–2021
Source: Front Nutr. 2025 Jul 1;12:1561712. doi: 10.3389/fnut.2025.1561712 (PMC12259433; doi:10.3389/fnut.2025.1561712)
Supplement: Supplementary file 1 [file Table_1.docx]

***Supplementary Materials***

Global, Regional, and National Burden of Gallbladder and Biliary Tract Cancer Among Adults Aged 55 Years and Older, 2010–2021

[**1.** **Section 1: The flowchart of estimation for Gallbladder and Biliary Tract Diseases.** 3](#_Toc199066339)

[**2.** **Section 2: Supplementary Tables** 4](#_Toc199066340)

[**Table1 Deaths, incidence cases, and DALYs for GBTC percentage change from 2010 to 2021 in 21 regions burden for the population aged 55 years and older of disease.** 4](#_Toc199066341)

[**Table 2-S1. The incidence of GBTC between 2010 and 2021 at national level, both sexes in aged 55 years and older** 5](#_Toc199066342)

[**Table** 2-**S2. The deaths of GBTC between 2010 and 2021 at national level, both sexes in aged 55 years and older** 14](#_Toc199066343)

[**Table 2-S3. The DALYs of GBTC between 2010 and 2021 at national level, both sexes in aged 55 years and older** 22](#_Toc199066344)

[**3.** **Section 2: Supplementary Figures** 31](#_Toc199066345)

[**Supplemental Figure 1** The ASDR of GBTC in 2021, for aged 55 years and older. 31](#_Toc199066346)

[**Supplemental Figure 2** The death cases of GBTC burden of 204 countries and territories in 2021, for aged 55 years and older. 32](#_Toc199066347)

[**Supplemental Figure 3** The ASMR of GBTC burden of 204 countries and territories in 2021 for aged 55 years and older. 33](#_Toc199066348)

[**Supplemental Figure 4** The ASMR percentage change of GBTC from 2010 to 2021, for aged 55 years and older. 34](#_Toc199066349)

[**Supplemental Figure 5** The DALY of GBTC burden in 2021, at the aged 55 years and older. 35](#_Toc199066350)

[**Supplemental Figure 6** The ASDR of GBTC burden of 204 countries and territories in 2021 for aged 55 years and older. 36](#_Toc199066351)

[**Supplemental Figure 7** The ASDR percentage change of GBTC from 2010 to 2021 for aged 55 years and older. 37](#_Toc199066352)

[**Supplemental Figure 8** The trend in (A) ASIR and (B) ASMR in aged 55 years and older of GBTC across 21 GBD regions by SDI for both sexes, from 2010 to 2021. Expected values are shown as the black line. 39](#_Toc199066353)

[**Supplemental Figure 9** The trend in (A) ASIR and (B) ASMR in aged 55 years and older of GBTC across 204 countries and territories by SDI for both sexes, from 2010 to 2021. Expected values are shown as the black line. 41](#_Toc199066354)

[**Supplemental Figure 10** The distribution of GBTC risk factor in sex, regions, and aged 55 years and older. 43](#_Toc199066355)

1. **Section 1: The flowchart of estimation for Gallbladder and Biliary Tract Diseases.**

**
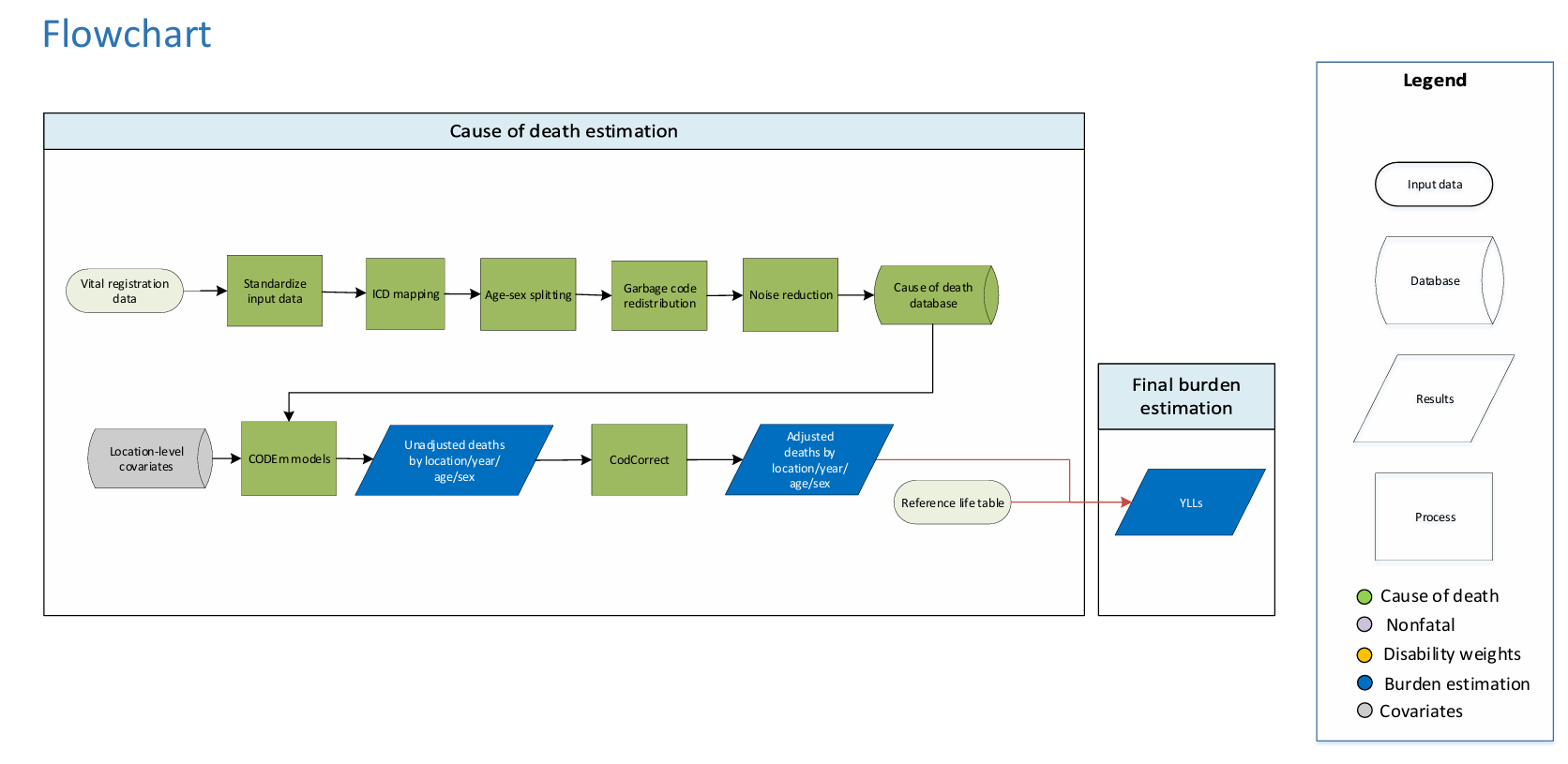
**

This flowchart download in [GBD data and tools guide | Institute for Health Metrics and Evaluation](https://www.healthdata.org/research-analysis/about-gbd/gbd-data-and-tools-guide), illustrates the systematic progression from raw data harmonization and correction to comprehensive burden quantification. It emphasizes rigorous data standardization, covariate adjustments, and multidimensional analysis, reflecting the GBD framework’s emphasis on methodological transparency and accuracy(1,2).

Reference

1. Global incidence, prevalence, years lived with disability (YLDs), disability-adjusted life-years (DALYs), and healthy life expectancy (HALE) for 371 diseases and injuries in 204 countries and territories and 811 subnational locations, 1990–2021: A systematic analysis for the global burden of disease study 2021. *Lancet (lond Engl)* (2024) 403:2133–2161. doi: 10.1016/S0140-6736(24)00757-8

2. Ouyang G, Pan G, Liu Q, Wu Y, Liu Z, Lu W, Li S, Zhou Z, Wen Y. The global, regional, and national burden of pancreatitis in 195 countries and territories, 1990–2017: A systematic analysis for the global burden of disease study 2017. *BMC Med* (2020) 18:388. doi: 10.1186/s12916-020-01859-5

1. **Section 2: Supplementary Tables**

**Table1 Deaths, incidence cases, and DALYs for GBTC percentage change from 2010 to 2021 in 21 regions burden for the population aged 55 years and older of disease.**

|  | | | |
| --- | --- | --- | --- |
| Location name | Percentage change in incidence number (95% UI) | Percentage change in death number (95% UI) | Percentage change in DALYs number (95% UI) |
| Andean Latin America | 39.9% (13.8% to 73.0%) | 34.1% (9.4% to 65.5%) | 35.5% (9.5% to 68.8%) |
| Australasia | 21.7% (13.0% to 30.4%) | 24.5% (18.4% to 30.5%) | 23.0% (16.7% to 28.7%) |
| Caribbean | 16.2% (3.7% to 27.2%) | 14.2% (1.6% to 24.9%) | 15.8% (3.1% to 27.5%) |
| Central Asia | 57.7% (39.2% to 80.5%) | 53.1% (35.0% to 75.4%) | 60.3% (40.5% to 83.8%) |
| Central Europe | 3.1% (-4.3% to 10.5%) | -0.2% (-7.0% to 6.5%) | -2.1% (-9.0% to 4.8%) |
| Central Latin America | 37.3% (23.5% to 51.4%) | 33.0% (19.6% to 46.1%) | 36.1% (21.7% to 50.8%) |
| Central Sub-Saharan Africa | 54.5% (25.2% to 88.0%) | 52.8% (24.7% to 87.3%) | 55.6% (24.3% to 91.9%) |
| East Asia | 58.0% (28.6% to 94.0%) | 36.7% (11.6% to 66.8%) | 33.2% (7.5% to 63.4%) |
| Eastern Europe | 26.9% (18.6% to 36.2%) | 12.0% (4.2% to 21.0%) | 10.7% (2.8% to 20.4%) |
| Eastern Sub-Saharan Africa | 46.0% (29.4% to 62.8%) | 45.4% (29.0% to 61.9%) | 43.9% (26.9% to 60.3%) |
| High-income Asia Pacific | 24.5% (16.1% to 31.9%) | 19.0% (12.3% to 25.0%) | 7.3% (1.0% to 13.0%) |
| High-income North America | 24.4% (21.2% to 27.6%) | 20.8% (17.9% to 23.8%) | 23.1% (20.2% to 26.0%) |
| North Africa and Middle East | 41.7% (28.5% to 56.4%) | 35.2% (22.9% to 49.0%) | 35.6% (22.3% to 49.8%) |
| Oceania | 40.3% (16.9% to 63.2%) | 39.0% (15.6% to 62.6%) | 41.2% (17.4% to 66.6%) |
| South Asia | 60.5% (41.6% to 79.6%) | 57.9% (39.9% to 76.1%) | 56.3% (38.3% to 75.2%) |
| Southeast Asia | 66.4% (33.9% to 108.6%) | 59.5% (29.3% to 98.1%) | 58.5% (28.7% to 97.2%) |
| Southern Latin America | 2.9% (-2.4% to 9.1%) | -2.6% (-7.4% to 3.5%) | -2.8% (-7.8% to 3.2%) |
| Southern Sub-Saharan Africa | 31.2% (13.5% to 49.6%) | 28.6% (11.5% to 46.5%) | 30.0% (12.1% to 49.5%) |
| Tropical Latin America | 46.0% (41.2% to 51.2%) | 41.9% (37.4% to 47.0%) | 43.7% (38.4% to 48.9%) |
| Western Europe | 13.0% (8.9% to 16.7%) | 7.2% (3.8% to 10.6%) | 3.6% (0.6% to 6.7%) |
| Western Sub-Saharan Africa | 55.2% (26.3% to 82.3%) | 53.7% (25.4% to 80.7%) | 55.6% (26.9% to 85.7%) |

* GBTC represents gallbladder and biliary tract cancer, ASR represents age-standardized rate.

**Table 2-S1. The incidence of GBTC between 2010 and 2021 at national level, both sexes in aged 55 years and older**

|  | | | | | | |
| --- | --- | --- | --- | --- | --- | --- |
| region | Case in 2010 | Case in 2021 | Change in number (95% UI) | ASR in 2010 | ASR in 2021 | change in ASR per 100 000 population (95% UI) |
| People's Republic of China | 27903(19691 to 32602) | 44513(30655 to 57373) | 59.5(29.2 to 97) | 10.8(7.6 to 12.6) | 11.7(8.1 to 15.1) | 8.7(-12 to 34.3) |
| Democratic People's Republic of Korea | 335(229 to 585) | 377(252 to 682) | 12.6(-8.3 to 37.2) | 7.8(5.3 to 13.6) | 6.7(4.5 to 12.1) | -13.9(-29.8 to 5) |
| Taiwan (Province of China) | 881(814 to 942) | 1110(988 to 1229) | 26(13.6 to 38.1) | 17.3(15.9 to 18.5) | 14.7(13.1 to 16.3) | -14.5(-23 to -6.4) |
| Kingdom of Cambodia | 56(38 to 118) | 100(63 to 217) | 78(28.8 to 150.1) | 4.1(2.8 to 8.7) | 4.6(2.9 to 10) | 11.7(-19.2 to 56.9) |
| Republic of Indonesia | 855(585 to 1840) | 1244(816 to 2691) | 45.4(14.6 to 75.5) | 3.2(2.2 to 6.8) | 3(1.9 to 6.4) | -6.4(-26.2 to 13) |
| Lao People's Democratic Republic | 20(12 to 42) | 28(18 to 63) | 43.5(7.5 to 84.5) | 3.8(2.3 to 8.1) | 3.6(2.3 to 8.1) | -3.3(-27.6 to 24.2) |
| Malaysia | 158(107 to 195) | 262(168 to 320) | 65.2(40.5 to 98.9) | 4.9(3.3 to 6) | 5.3(3.4 to 6.5) | 9.1(-7.3 to 31.3) |
| Republic of Maldives | 1(1 to 1) | 1(1 to 2) | 35.9(5.3 to 70.9) | 3.5(2.3 to 4.1) | 2.7(1.8 to 3.4) | -24.8(-41.8 to -5.5) |
| Republic of the Union of Myanmar | 187(116 to 385) | 265(173 to 568) | 41.9(10.5 to 82.8) | 3.2(2 to 6.7) | 3.1(2 to 6.7) | -2.6(-24.2 to 25.5) |
| Republic of the Philippines | 199(171 to 352) | 310(244 to 555) | 55.8(30.7 to 83.9) | 2.1(1.8 to 3.8) | 2.2(1.8 to 4) | 3.9(-12.9 to 22.6) |
| Democratic Socialist Republic of Sri Lanka | 135(108 to 247) | 188(114 to 354) | 39.2(-11 to 87.8) | 4(3.2 to 7.3) | 3.9(2.4 to 7.4) | -2.5(-37.7 to 31.5) |
| Kingdom of Thailand | 3810(2170 to 4667) | 6640(3390 to 9008) | 74.3(26.3 to 135.7) | 31.2(17.8 to 38.2) | 34.2(17.4 to 46.3) | 9.6(-20.6 to 48.2) |
| Democratic Republic of Timor-Leste | 3(2 to 7) | 4(3 to 9) | 45.5(16.6 to 78.4) | 2.7(1.9 to 5.9) | 3.1(2 to 6.7) | 13.8(-8.8 to 39.6) |
| Socialist Republic of Viet Nam | 622(492 to 859) | 1022(697 to 1507) | 64.2(22.4 to 115.2) | 5.8(4.6 to 8) | 5.8(4 to 8.6) | 0.7(-24.9 to 32.1) |
| Republic of Fiji | 5(3 to 6) | 6(4 to 9) | 31.6(-2.2 to 73.6) | 4.7(2.9 to 5.6) | 4.6(2.7 to 6.3) | -2.6(-27.6 to 28.5) |
| Republic of Kiribati | 0(0 to 0) | 0(0 to 0) | 33.9(8.3 to 64.7) | 2.6(1.3 to 3.4) | 2.4(1.2 to 3.2) | -8.9(-26.3 to 12) |
| Republic of the Marshall Islands | 0(0 to 0) | 0(0 to 0) | 37.9(10.1 to 74) | 2.7(1.7 to 3.7) | 2.6(1.6 to 3.6) | -6.1(-25 to 18.5) |
| Federated States of Micronesia | 0(0 to 0) | 0(0 to 0) | 25.8(-1.1 to 57.7) | 2.9(1.9 to 3.9) | 2.7(1.8 to 3.7) | -9.5(-28.8 to 13.5) |
| Independent State of Papua New Guinea | 10(6 to 14) | 15(10 to 22) | 52(17.1 to 93.1) | 2(1.3 to 2.9) | 1.9(1.2 to 2.6) | -6.9(-28.2 to 18.4) |
| Independent State of Samoa | 1(0 to 1) | 1(0 to 1) | 17.1(-5.4 to 42.3) | 3(2 to 3.9) | 2.8(1.9 to 3.7) | -8(-25.7 to 11.8) |
| Solomon Islands | 1(1 to 1) | 1(1 to 2) | 41.1(10.4 to 78.1) | 2.4(1.4 to 3.4) | 2.5(1.6 to 3.5) | 2(-20.1 to 28.8) |
| Kingdom of Tonga | 0(0 to 0) | 0(0 to 0) | 5.4(-15.7 to 28.5) | 2.5(1.7 to 3.4) | 2.3(1.6 to 3.3) | -7.4(-25.9 to 13) |
| Republic of Vanuatu | 0(0 to 1) | 1(0 to 1) | 45.9(20.9 to 77.4) | 2.3(1.5 to 3.1) | 2.2(1.5 to 3) | -0.8(-17.8 to 20.6) |
| Republic of Armenia | 23(20 to 27) | 64(54 to 75) | 173.1(118.7 to 240.2) | 3.8(3.2 to 4.3) | 8.1(6.8 to 9.6) | 115.6(72.6 to 168.5) |
| Republic of Azerbaijan | 43(28 to 63) | 56(33 to 84) | 29.8(-18.9 to 99.8) | 3.9(2.5 to 5.7) | 2.9(1.7 to 4.4) | -25.7(-53.6 to 14.3) |
| Georgia | 46(38 to 55) | 84(70 to 102) | 84.1(47.3 to 131.1) | 4.6(3.8 to 5.4) | 8(6.6 to 9.7) | 76.3(41.1 to 121.4) |
| Republic of Kazakhstan | 144(131 to 158) | 155(128 to 184) | 7.9(-12.9 to 29.4) | 6.2(5.7 to 6.9) | 4.9(4 to 5.8) | -21.7(-36.7 to -6) |
| Kyrgyz Republic | 20(17 to 24) | 39(32 to 48) | 93.3(51.2 to 143.7) | 3.7(3.1 to 4.3) | 4.6(3.8 to 5.6) | 24(-3 to 56.4) |
| Mongolia | 30(22 to 45) | 46(33 to 69) | 52.4(2.6 to 114.4) | 13.7(9.9 to 20.5) | 11.7(8.3 to 17.6) | -14.9(-42.7 to 19.7) |
| Republic of Tajikistan | 4(3 to 5) | 5(3 to 7) | 29.2(-11.9 to 89.9) | 0.7(0.5 to 1) | 0.5(0.3 to 0.7) | -32(-53.6 to 0) |
| Turkmenistan | 11(10 to 13) | 24(19 to 32) | 121.8(68.1 to 194.9) | 2.5(2.2 to 2.8) | 3.5(2.6 to 4.5) | 38.8(5.2 to 84.6) |
| Republic of Uzbekistan | 28(23 to 35) | 78(60 to 99) | 174.2(103 to 270.7) | 1(0.8 to 1.3) | 1.7(1.3 to 2.1) | 61.5(19.6 to 118.4) |
| Republic of Albania | 38(24 to 58) | 53(36 to 78) | 40.2(-7.1 to 109.1) | 6.3(4.1 to 9.8) | 6.7(4.5 to 9.9) | 6.6(-29.4 to 58.9) |
| Bosnia and Herzegovina | 183(147 to 301) | 186(136 to 318) | 1.7(-22.9 to 30.3) | 18.4(14.8 to 30.2) | 17(12.4 to 29) | -7.5(-29.9 to 18.5) |
| Republic of Bulgaria | 240(220 to 259) | 225(189 to 266) | -6.4(-21.6 to 12.1) | 10(9.2 to 10.8) | 9.5(7.9 to 11.2) | -5.6(-21 to 13) |
| Republic of Croatia | 313(282 to 346) | 353(297 to 405) | 12.7(-5 to 31.6) | 23.3(21 to 25.8) | 23.6(19.9 to 27.2) | 1.6(-14.3 to 18.6) |
| Czech Republic | 934(801 to 1039) | 946(795 to 1113) | 1.3(-15.5 to 21.2) | 29.7(25.5 to 33) | 27.1(22.7 to 31.9) | -8.8(-24 to 9.1) |
| Hungary | 656(605 to 704) | 579(504 to 651) | -11.7(-22.3 to -0.3) | 21.5(19.9 to 23.1) | 18.2(15.8 to 20.5) | -15.4(-25.6 to -4.4) |
| North Macedonia | 65(49 to 80) | 71(52 to 92) | 9.6(-14.1 to 35.7) | 13.5(10 to 16.4) | 12(8.8 to 15.6) | -10.6(-30 to 10.7) |
| Montenegro | 13(10 to 17) | 15(11 to 20) | 13.9(-15 to 49.7) | 8.5(6.2 to 11.1) | 8.6(6.2 to 11.6) | 1(-24.6 to 32.7) |
| Republic of Poland | 1915(1783 to 1993) | 1922(1738 to 2085) | 0.4(-7.3 to 8.1) | 18.6(17.3 to 19.4) | 15.9(14.3 to 17.2) | -14.7(-21.2 to -8.1) |
| Romania | 509(471 to 545) | 540(470 to 612) | 6.1(-8.8 to 21) | 8.7(8 to 9.3) | 9(7.8 to 10.2) | 3.7(-10.8 to 18.3) |
| Republic of Serbia | 329(245 to 421) | 349(243 to 465) | 6.1(-20.7 to 37.3) | 12.7(9.4 to 16.2) | 12.5(8.7 to 16.6) | -1.8(-26.6 to 27) |
| Slovak Republic | 391(303 to 499) | 514(365 to 699) | 31.4(-1.3 to 70) | 28.9(22.4 to 36.9) | 31.4(22.2 to 42.6) | 8.5(-18.6 to 40.3) |
| Republic of Slovenia | 147(132 to 162) | 162(136 to 189) | 9.6(-6.4 to 27.4) | 24.5(22 to 26.9) | 22.1(18.5 to 25.8) | -9.9(-23.1 to 4.8) |
| Republic of Belarus | 198(177 to 217) | 220(179 to 269) | 11.1(-8.6 to 37.5) | 8.1(7.2 to 8.8) | 7.7(6.2 to 9.4) | -5.2(-22 to 17.4) |
| Republic of Estonia | 40(36 to 44) | 53(45 to 60) | 31.4(14 to 49.2) | 10.2(9.1 to 11.1) | 12.1(10.3 to 13.7) | 18.6(2.9 to 34.6) |
| Republic of Latvia | 45(42 to 49) | 51(44 to 58) | 12.1(-2.4 to 29) | 7.1(6.6 to 7.7) | 7.7(6.7 to 8.8) | 8(-6 to 24.3) |
| Republic of Lithuania | 87(78 to 95) | 122(106 to 138) | 40.3(21 to 59.1) | 9.8(8.8 to 10.8) | 12.6(11 to 14.3) | 28.3(10.6 to 45.5) |
| Republic of Moldova | 43(40 to 45) | 48(44 to 54) | 12.4(0.3 to 26.8) | 4.8(4.5 to 5.1) | 4.6(4.1 to 5.1) | -5.7(-15.9 to 6.3) |
| Russian Federation | 3328(3167 to 3423) | 4626(4247 to 4994) | 39(28.6 to 49.7) | 9.3(8.8 to 9.5) | 10.9(10 to 11.7) | 17.6(8.8 to 26.7) |
| Ukraine | 913(855 to 966) | 787(590 to 1012) | -13.8(-35.2 to 12.8) | 7.4(6.9 to 7.8) | 5.8(4.3 to 7.5) | -21.2(-40.8 to 3) |
| Brunei Darussalam | 4(3 to 5) | 6(5 to 8) | 46.8(23.3 to 77.7) | 12.1(9.2 to 15.1) | 10.2(8 to 13.3) | -15.8(-29.3 to 1.9) |
| Japan | 24172(20525 to 26159) | 28358(22977 to 31518) | 17.3(10.3 to 23.6) | 49.7(42.2 to 53.8) | 54.3(44 to 60.4) | 9.4(2.8 to 15.2) |
| Republic of Korea | 4448(3167 to 5336) | 7248(4442 to 9240) | 63(33.6 to 96.6) | 42.5(30.3 to 51) | 43.3(26.6 to 55.3) | 1.9(-16.5 to 22.9) |
| Republic of Singapore | 67(61 to 72) | 105(93 to 115) | 56.1(41.8 to 70.4) | 7.7(7 to 8.3) | 6.9(6.1 to 7.6) | -10.5(-18.7 to -2.2) |
| Australia | 1242(1108 to 1335) | 1393(1195 to 1519) | 12.1(3.3 to 21.1) | 22.9(20.4 to 24.6) | 18.8(16.1 to 20.5) | -17.9(-24.4 to -11.3) |
| New Zealand | 135(120 to 148) | 282(247 to 313) | 109.5(91.8 to 128) | 12.8(11.4 to 14.1) | 19.8(17.3 to 21.9) | 54.1(41.1 to 67.7) |
| Principality of Andorra | 4(3 to 6) | 4(3 to 6) | -1.4(-27 to 30.5) | 20.8(14.8 to 28.1) | 15.4(10.4 to 20.9) | -25.8(-45 to -1.7) |
| Republic of Austria | 418(372 to 448) | 415(362 to 456) | -0.6(-8.3 to 7.6) | 17.1(15.2 to 18.3) | 14.1(12.2 to 15.4) | -17.8(-24.1 to -11) |
| Kingdom of Belgium | 324(284 to 353) | 295(253 to 327) | -9(-15.8 to -1.4) | 10.1(8.9 to 11) | 7.8(6.7 to 8.6) | -23(-28.8 to -16.5) |
| Republic of Cyprus | 33(25 to 41) | 39(28 to 52) | 18(-5 to 48.5) | 13.5(10.4 to 16.8) | 11.1(8.1 to 14.8) | -17.6(-33.7 to 3.6) |
| Kingdom of Denmark | 203(187 to 218) | 233(207 to 256) | 15(4.3 to 25.5) | 12.4(11.5 to 13.4) | 12.1(10.8 to 13.3) | -2.6(-11.7 to 6.3) |
| Republic of Finland | 255(225 to 277) | 333(285 to 370) | 30.5(17.1 to 42.4) | 14.7(13 to 16) | 16.5(14.2 to 18.4) | 12.4(0.9 to 22.6) |
| French Republic | 2155(1891 to 2336) | 2158(1818 to 2442) | 0.1(-9.8 to 10.3) | 11.6(10.2 to 12.6) | 9.8(8.2 to 11) | -16(-24.3 to -7.4) |
| Federal Republic of Germany | 5477(4923 to 5827) | 6323(5461 to 6907) | 15.4(7.2 to 24.3) | 20.4(18.3 to 21.7) | 20.1(17.3 to 21.9) | -1.7(-8.7 to 5.9) |
| Hellenic Republic | 698(636 to 749) | 455(405 to 493) | -34.8(-39.5 to -29.4) | 20.6(18.8 to 22.1) | 12.4(11 to 13.4) | -40(-44.3 to -35.1) |
| Republic of Iceland | 8(7 to 9) | 9(8 to 11) | 14.5(1.8 to 28.1) | 11.4(10.2 to 12.5) | 9.6(8.3 to 10.9) | -15.6(-25 to -5.6) |
| Ireland | 83(74 to 91) | 93(80 to 104) | 12.6(1.6 to 22.6) | 8.5(7.6 to 9.3) | 7.1(6.1 to 7.9) | -16.1(-24.3 to -8.6) |
| State of Israel | 123(109 to 134) | 133(115 to 148) | 7.6(-2.9 to 17.8) | 8.3(7.3 to 9) | 6.6(5.7 to 7.4) | -20.1(-27.9 to -12.5) |
| Republic of Italy | 4696(4079 to 5062) | 4820(4150 to 5292) | 2.6(-2 to 7.8) | 24(20.8 to 25.8) | 21.2(18.3 to 23.3) | -11.5(-15.5 to -7) |
| Grand Duchy of Luxembourg | 13(12 to 14) | 15(13 to 17) | 19.2(6.7 to 32.8) | 10.4(9.4 to 11.2) | 8.7(7.6 to 9.8) | -16.1(-24.9 to -6.6) |
| Republic of Malta | 9(8 to 9) | 10(9 to 12) | 21.5(6.7 to 35.9) | 6.8(6.1 to 7.4) | 6.6(5.7 to 7.4) | -2.7(-14.5 to 8.9) |
| Kingdom of the Netherlands | 694(618 to 747) | 841(745 to 931) | 21.2(10.4 to 32.7) | 14.8(13.1 to 15.9) | 14.4(12.7 to 15.9) | -2.6(-11.2 to 6.7) |
| Kingdom of Norway | 149(132 to 160) | 189(165 to 206) | 26.9(18.6 to 35.4) | 11.3(10 to 12.2) | 11.7(10.2 to 12.7) | 2.8(-3.9 to 9.7) |
| Portuguese Republic | 462(413 to 502) | 546(481 to 602) | 18.2(9.1 to 29.2) | 14(12.5 to 15.2) | 14(12.4 to 15.5) | 0.3(-7.4 to 9.7) |
| Kingdom of Spain | 2489(2170 to 2716) | 2877(2471 to 3214) | 15.6(5.4 to 26) | 19.1(16.7 to 20.9) | 18.6(16 to 20.8) | -2.8(-11.4 to 5.9) |
| Kingdom of Sweden | 459(411 to 493) | 576(494 to 649) | 25.4(12.7 to 39.1) | 15.6(14 to 16.8) | 17.1(14.7 to 19.3) | 9.5(-1.6 to 21.5) |
| Swiss Confederation | 376(324 to 421) | 489(405 to 564) | 30(16.7 to 44.3) | 16.4(14.1 to 18.3) | 16.7(13.8 to 19.2) | 2(-8.5 to 13.2) |
| United Kingdom of Great Britain and Northern Ireland | 2113(1928 to 2207) | 3142(2805 to 3311) | 48.7(43.9 to 52.9) | 11.9(10.9 to 12.5) | 14.9(13.3 to 15.7) | 25.1(21 to 28.6) |
| Argentine Republic | 1692(1585 to 1794) | 1613(1475 to 1723) | -4.6(-10.9 to 3.1) | 22(20.6 to 23.3) | 17.3(15.8 to 18.5) | -21.3(-26.4 to -14.9) |
| Republic of Chile | 1910(1763 to 2022) | 2083(1879 to 2279) | 9(0.7 to 18.3) | 62.9(58 to 66.5) | 46.5(42 to 50.9) | -26(-31.7 to -19.8) |
| Eastern Republic of Uruguay | 205(187 to 219) | 220(199 to 242) | 7.5(-1.4 to 17.9) | 26(23.7 to 27.8) | 24.4(22 to 26.7) | -6.4(-14.2 to 2.6) |
| Canada | 1143(1012 to 1236) | 1209(1045 to 1326) | 5.8(-3.1 to 15) | 12.6(11.1 to 13.6) | 9.9(8.5 to 10.8) | -21.7(-28.3 to -14.9) |
| United States of America | 9193(8281 to 9679) | 11643(10470 to 12307) | 26.7(23.4 to 29.5) | 12.1(10.9 to 12.7) | 11.6(10.4 to 12.3) | -3.6(-6.1 to -1.4) |
| Antigua and Barbuda | 1(1 to 1) | 1(1 to 1) | 19.5(9.7 to 29.8) | 6.9(6.3 to 7.3) | 5.5(5.1 to 5.8) | -20.1(-26.6 to -13.2) |
| Commonwealth of the Bahamas | 4(3 to 4) | 5(4 to 6) | 32.2(8.7 to 64.6) | 7.6(7 to 8.2) | 6.5(5.4 to 7.9) | -13.8(-29.1 to 7.3) |
| Barbados | 5(5 to 5) | 6(5 to 8) | 29.5(1.4 to 60.2) | 7.4(6.9 to 7.9) | 6.9(5.3 to 8.5) | -7.7(-27.8 to 14.2) |
| Belize | 2(2 to 2) | 2(2 to 3) | 28.4(12.7 to 46.1) | 6.3(5.9 to 6.7) | 4.6(4 to 5.2) | -26.6(-35.5 to -16.5) |
| Republic of Cuba | 169(158 to 180) | 176(152 to 197) | 4.1(-10.2 to 19.1) | 6.5(6 to 6.9) | 5.1(4.4 to 5.8) | -20.3(-31.2 to -8.7) |
| Commonwealth of Dominica | 1(1 to 1) | 1(1 to 1) | 13.3(-6.3 to 41.1) | 9(6.7 to 11.5) | 7.8(5.7 to 9.8) | -13.7(-28.6 to 7.4) |
| Dominican Republic | 46(35 to 66) | 65(49 to 94) | 43.3(9.4 to 86.4) | 3.8(2.9 to 5.5) | 3.9(2.9 to 5.6) | 3(-21.3 to 34) |
| Grenada | 1(1 to 1) | 1(1 to 1) | 8.5(-5.7 to 23.8) | 7.4(6.8 to 8) | 6.5(5.6 to 7.4) | -12.5(-23.9 to -0.2) |
| Republic of Guyana | 4(4 to 5) | 5(4 to 7) | 22.3(-3.5 to 54.4) | 5.3(4.8 to 5.8) | 4.7(3.7 to 5.9) | -10.5(-29.3 to 13.1) |
| Republic of Haiti | 62(36 to 92) | 77(48 to 116) | 24.9(-1.2 to 60) | 7.2(4.2 to 10.7) | 6.5(4.1 to 9.8) | -9.5(-28.4 to 15.8) |
| Jamaica | 23(21 to 25) | 28(22 to 36) | 21.6(-5.6 to 56.3) | 5.7(5.2 to 6.1) | 5.3(4.2 to 6.9) | -5.6(-26.7 to 21.3) |
| Saint Lucia | 1(1 to 1) | 2(1 to 2) | 53.5(24.7 to 86.6) | 4(3.7 to 4.2) | 3.9(3.2 to 4.7) | -3.2(-21.4 to 17.6) |
| Saint Vincent and the Grenadines | 1(1 to 1) | 1(1 to 1) | 17.5(1.9 to 36) | 5(4.6 to 5.3) | 4(3.5 to 4.5) | -19.9(-30.6 to -7.3) |
| Republic of Suriname | 4(3 to 5) | 5(3 to 6) | 29.1(-3.8 to 69.5) | 4.9(4 to 6.6) | 4.2(3 to 5.7) | -16(-37.5 to 10.3) |
| Republic of Trinidad and Tobago | 12(11 to 12) | 17(13 to 21) | 43(10.2 to 80.9) | 4.8(4.5 to 5) | 4.8(3.7 to 6) | 0(-22.9 to 26.6) |
| Plurinational State of Bolivia | 322(206 to 452) | 415(266 to 603) | 28.8(3.8 to 65.2) | 29(18.6 to 40.7) | 26.9(17.2 to 39) | -7.5(-25.4 to 18.7) |
| Republic of Ecuador | 349(318 to 384) | 412(328 to 508) | 18.1(-7.7 to 49.8) | 18.7(17 to 20.5) | 14.9(11.8 to 18.3) | -20.2(-37.6 to 1.2) |
| Republic of Peru | 760(551 to 947) | 1175(820 to 1643) | 54.6(10.2 to 108.2) | 19.8(14.4 to 24.7) | 21(14.7 to 29.4) | 5.8(-24.6 to 42.5) |
| Republic of Colombia | 817(753 to 876) | 1193(997 to 1398) | 46(23.3 to 70.4) | 12.8(11.8 to 13.8) | 12.5(10.4 to 14.6) | -2.9(-18 to 13.3) |
| Republic of Costa Rica | 65(59 to 70) | 96(84 to 108) | 47.1(29 to 65.9) | 10.6(9.6 to 11.4) | 10(8.7 to 11.3) | -5.2(-16.9 to 6.9) |
| Republic of El Salvador | 95(77 to 135) | 131(102 to 183) | 38.7(12.3 to 70.1) | 11.8(9.7 to 16.8) | 12.8(9.9 to 17.9) | 8.5(-12.1 to 33) |
| Republic of Guatemala | 125(118 to 130) | 137(119 to 159) | 10(-5.7 to 28.4) | 10.1(9.6 to 10.5) | 7.5(6.5 to 8.7) | -25.9(-36.5 to -13.5) |
| Republic of Honduras | 103(71 to 141) | 162(110 to 216) | 57(17.6 to 102.8) | 14.5(10 to 19.8) | 15.4(10.5 to 20.5) | 6(-20.7 to 36.9) |
| United Mexican States | 1708(1636 to 1751) | 2254(1992 to 2527) | 31.9(17.1 to 46.5) | 12.1(11.5 to 12.4) | 10.5(9.2 to 11.7) | -13.3(-23 to -3.6) |
| Republic of Nicaragua | 64(52 to 88) | 83(64 to 129) | 28.8(3.3 to 61.4) | 12(9.8 to 16.4) | 10.2(7.8 to 15.8) | -15.5(-32.2 to 6) |
| Republic of Panama | 34(31 to 36) | 47(37 to 55) | 39(9.4 to 65.1) | 6.7(6.2 to 7.2) | 6.3(5 to 7.4) | -7.1(-26.9 to 10.4) |
| Bolivarian Republic of Venezuela | 269(251 to 283) | 402(302 to 513) | 49.5(11.3 to 93.2) | 7.7(7.2 to 8.1) | 7.7(5.8 to 9.8) | -0.5(-25.9 to 28.6) |
| Federative Republic of Brazil | 3289(3046 to 3436) | 4804(4368 to 5058) | 46.1(41.1 to 51.1) | 11.2(10.4 to 11.8) | 11.1(10.1 to 11.7) | -1.4(-4.7 to 2.1) |
| Republic of Paraguay | 63(46 to 82) | 90(62 to 127) | 42.6(7.2 to 85.3) | 9.2(6.6 to 11.9) | 9.1(6.3 to 12.8) | -1.2(-25.7 to 28.5) |
| People's Democratic Republic of Algeria | 562(406 to 748) | 854(611 to 1124) | 52(23.2 to 85.8) | 14.5(10.4 to 19.2) | 14.1(10.1 to 18.5) | -2.8(-21.2 to 18.9) |
| Kingdom of Bahrain | 3(2 to 4) | 5(4 to 8) | 88.7(41.1 to 155) | 3.8(2.5 to 4.6) | 3.3(2.2 to 4.6) | -11.5(-33.8 to 19.6) |
| Arab Republic of Egypt | 458(328 to 540) | 637(447 to 795) | 39.1(11.8 to 74.3) | 6.1(4.3 to 7.2) | 5.8(4 to 7.2) | -5.2(-23.8 to 18.8) |
| Islamic Republic of Iran | 360(199 to 419) | 524(290 to 610) | 45.8(34.2 to 57.6) | 4.3(2.4 to 5) | 4(2.2 to 4.7) | -5.1(-12.6 to 2.6) |
| Republic of Iraq | 103(73 to 139) | 184(127 to 239) | 78.4(31.9 to 134) | 4.2(3 to 5.7) | 4.7(3.3 to 6.2) | 12.8(-16.7 to 47.8) |
| Hashemite Kingdom of Jordan | 37(25 to 46) | 69(47 to 96) | 83.6(41 to 137.5) | 7.1(4.8 to 8.8) | 5.5(3.7 to 7.6) | -22.9(-40.8 to -0.2) |
| State of Kuwait | 15(14 to 16) | 25(20 to 30) | 66(37 to 96.9) | 7.3(6.6 to 7.9) | 5.4(4.4 to 6.4) | -26.2(-39 to -12.4) |
| Lebanese Republic | 71(43 to 90) | 88(54 to 115) | 23.4(-0.4 to 53.6) | 9.9(6 to 12.5) | 8.9(5.5 to 11.8) | -9.4(-26.9 to 12.7) |
| State of Libya | 99(72 to 121) | 136(91 to 184) | 37.8(6.7 to 76.9) | 19(14 to 23.4) | 16.2(10.9 to 21.9) | -14.7(-33.9 to 9.5) |
| Kingdom of Morocco | 126(77 to 169) | 190(110 to 242) | 50.1(16 to 91.1) | 3.2(1.9 to 4.2) | 3.2(1.8 to 4) | -0.3(-23 to 26.9) |
| Palestine | 19(12 to 23) | 25(16 to 31) | 28.8(3.8 to 61.9) | 7.4(4.8 to 8.9) | 5.8(3.7 to 7.2) | -22.4(-37.4 to -2.5) |
| Sultanate of Oman | 7(5 to 9) | 10(7 to 13) | 33.9(4.7 to 69.8) | 4.5(3.2 to 5.6) | 3.2(2.3 to 4.3) | -28.8(-44.3 to -9.7) |
| State of Qatar | 4(3 to 5) | 8(5 to 12) | 86.8(26.2 to 178.4) | 6.2(4.5 to 7.9) | 5(3.3 to 7.7) | -19(-45.3 to 20.8) |
| Kingdom of Saudi Arabia | 137(111 to 191) | 197(148 to 293) | 43.8(14.8 to 71.8) | 7.7(6.3 to 10.8) | 6.6(4.9 to 9.7) | -15.3(-32.4 to 1.2) |
| Syrian Arab Republic | 7(4 to 8) | 9(5 to 12) | 36.7(-0.1 to 77.1) | 0.4(0.2 to 0.5) | 0.4(0.2 to 0.5) | -8.4(-33.1 to 18.6) |
| Republic of Tunisia | 176(116 to 235) | 256(167 to 354) | 45.7(10 to 95.9) | 11.5(7.6 to 15.4) | 10.9(7.1 to 15.1) | -5(-28.2 to 27.7) |
| Republic of Turkey | 887(624 to 1080) | 1155(866 to 1538) | 30.2(0.1 to 65.1) | 8(5.6 to 9.7) | 7(5.2 to 9.3) | -12.5(-32.7 to 11) |
| United Arab Emirates | 28(20 to 39) | 61(45 to 88) | 116(55 to 217.4) | 15(10.3 to 20.7) | 8.6(6.2 to 12.2) | -42.9(-59 to -16.1) |
| Republic of Yemen | 67(47 to 103) | 102(66 to 152) | 50.9(18.1 to 94.2) | 4.4(3 to 6.7) | 4.5(2.9 to 6.7) | 3.4(-19.1 to 33) |
| Islamic Republic of Afghanistan | 103(52 to 173) | 103(52 to 161) | 0.7(-19.2 to 28.4) | 8.1(4.1 to 13.7) | 8.4(4.2 to 13.1) | 3.9(-16.6 to 32.5) |
| People's Republic of Bangladesh | 1252(881 to 1776) | 2043(1372 to 3127) | 63.2(25.6 to 109.2) | 8.8(6.2 to 12.5) | 8.7(5.9 to 13.4) | -0.7(-23.5 to 27.3) |
| Kingdom of Bhutan | 7(5 to 10) | 11(7 to 15) | 44.7(18.8 to 77) | 9.9(7.4 to 14.2) | 10.6(7.3 to 15.1) | 7.2(-11.9 to 31.1) |
| Republic of India | 13129(9077 to 15447) | 21569(14886 to 25381) | 64.3(42.7 to 86.7) | 9.2(6.4 to 10.9) | 10.7(7.4 to 12.6) | 16(0.8 to 31.8) |
| Federal Democratic Republic of Nepal | 243(182 to 340) | 398(291 to 549) | 63.9(27.1 to 108.9) | 8.4(6.3 to 11.8) | 10.1(7.4 to 13.9) | 19.3(-7.5 to 52) |
| Islamic Republic of Pakistan | 2125(1585 to 2742) | 2871(2070 to 3817) | 35.1(2.3 to 74.2) | 15.4(11.5 to 19.9) | 14.5(10.4 to 19.3) | -6.3(-29.1 to 20.8) |
| Republic of Angola | 19(13 to 25) | 31(20 to 42) | 60.8(20.1 to 111.6) | 1.6(1.1 to 2.1) | 1.6(1.1 to 2.2) | -2(-26.8 to 29) |
| Central African Republic | 4(2 to 6) | 5(3 to 8) | 26.2(1.7 to 58.7) | 1.6(1 to 2.5) | 1.4(0.9 to 2.3) | -9.5(-27 to 13.8) |
| Republic of the Congo | 5(4 to 7) | 8(5 to 10) | 48.7(19.5 to 92.7) | 1.8(1.3 to 2.5) | 1.8(1.2 to 2.3) | -4(-22.9 to 24.4) |
| Democratic Republic of the Congo | 51(33 to 81) | 80(51 to 122) | 56.2(22.9 to 98.8) | 1.3(0.8 to 2) | 1.3(0.8 to 2) | 3.9(-18.3 to 32.2) |
| Republic of Equatorial Guinea | 1(1 to 1) | 1(1 to 2) | 47.9(3.7 to 108.3) | 1.7(1 to 2.6) | 1.7(1 to 2.5) | 0.1(-29.8 to 41) |
| Gabonese Republic | 2(2 to 3) | 3(2 to 4) | 30.2(4.3 to 64.1) | 1.9(1.3 to 2.6) | 1.8(1.2 to 2.4) | -7.5(-25.9 to 16.6) |
| Republic of Burundi | 17(11 to 23) | 26(15 to 38) | 47.8(17.3 to 87) | 3.4(2.2 to 4.6) | 3.2(1.8 to 4.7) | -6.3(-25.6 to 18.6) |
| Union of the Comoros | 2(1 to 3) | 3(2 to 5) | 43.9(14.5 to 78.4) | 3.6(2.4 to 5.4) | 3.7(2.3 to 5.6) | 2.3(-18.6 to 26.7) |
| Republic of Djibouti | 2(1 to 3) | 3(2 to 6) | 72.7(32.3 to 133.6) | 3.3(1.9 to 5.1) | 3.3(1.9 to 5.3) | -0.3(-23.7 to 34.9) |
| State of Eritrea | 13(8 to 19) | 18(11 to 27) | 43.7(15.5 to 81.3) | 4.2(2.6 to 6.1) | 4.2(2.5 to 6.1) | -0.1(-19.7 to 26.1) |
| Federal Democratic Republic of Ethiopia | 288(196 to 398) | 426(294 to 601) | 47.7(18.8 to 78.1) | 5.8(4 to 8.1) | 6.2(4.3 to 8.8) | 6.4(-14.4 to 28.3) |
| Republic of Kenya | 125(94 to 177) | 189(136 to 270) | 51.4(25.1 to 84.8) | 5(3.8 to 7.2) | 5(3.6 to 7.2) | -0.1(-17.5 to 21.9) |
| Republic of Madagascar | 32(21 to 44) | 49(30 to 68) | 54.3(19.3 to 95) | 2.7(1.7 to 3.7) | 2.7(1.6 to 3.7) | -1.3(-23.7 to 24.7) |
| Republic of Malawi | 10(7 to 14) | 14(9 to 20) | 34.4(2 to 74.4) | 1.2(0.8 to 1.6) | 1.2(0.7 to 1.7) | -2.5(-25.9 to 26.6) |
| Republic of Mauritius | 8(7 to 9) | 13(12 to 14) | 62.8(49.9 to 75.7) | 3.6(3.3 to 3.8) | 3.8(3.5 to 4.1) | 7.2(-1.2 to 15.8) |
| Republic of Mozambique | 54(32 to 78) | 70(39 to 103) | 30.8(-0.3 to 71.1) | 3.9(2.3 to 5.7) | 3.9(2.2 to 5.8) | 1.2(-22.9 to 32.4) |
| Republic of Rwanda | 25(17 to 35) | 43(28 to 60) | 69.8(31.6 to 115.6) | 3.9(2.6 to 5.3) | 4.1(2.6 to 5.7) | 4.5(-19 to 32.6) |
| Republic of Seychelles | 1(1 to 1) | 1(1 to 1) | 21.6(-0.3 to 42.4) | 5.3(4.3 to 7.6) | 4(3.3 to 5.9) | -23.5(-37.2 to -10.3) |
| Federal Republic of Somalia | 25(15 to 42) | 33(19 to 55) | 32.7(3.1 to 66.5) | 3.3(2 to 5.6) | 3.5(2 to 5.8) | 5.6(-17.9 to 32.5) |
| United Republic of Tanzania | 109(73 to 151) | 151(99 to 207) | 38.6(7 to 78.8) | 3.7(2.5 to 5.2) | 3.7(2.4 to 5) | -2(-24.3 to 26.4) |
| Republic of Uganda | 56(41 to 75) | 82(56 to 115) | 46.6(12.3 to 93.9) | 3.5(2.6 to 4.8) | 3.5(2.4 to 4.9) | -2.3(-25.1 to 29.2) |
| Republic of Zambia | 30(20 to 39) | 44(27 to 59) | 46.1(9.9 to 88.6) | 4.3(2.9 to 5.6) | 4(2.5 to 5.5) | -5.1(-28.6 to 22.5) |
| Republic of Botswana | 6(4 to 8) | 7(5 to 10) | 20.8(-12.5 to 69.6) | 3.5(2.5 to 4.8) | 2.8(2 to 4.2) | -20(-42 to 12.3) |
| Kingdom of Lesotho | 7(5 to 11) | 8(5 to 11) | 5.1(-16.9 to 37.7) | 4(2.7 to 6) | 4.2(2.8 to 6.3) | 5.1(-17 to 37.6) |
| Republic of Namibia | 4(3 to 5) | 5(4 to 8) | 33.1(-3 to 72.9) | 2.5(1.7 to 3.2) | 2.4(1.6 to 3.4) | -2.6(-29 to 26.6) |
| Republic of South Africa | 213(152 to 251) | 287(187 to 339) | 34.8(14.9 to 55.4) | 3.7(2.7 to 4.4) | 3.6(2.4 to 4.3) | -2.6(-17 to 12.3) |
| Kingdom of Eswatini | 3(2 to 5) | 4(2 to 5) | 12.9(-15.5 to 61) | 4.4(2.5 to 6.8) | 4(2.6 to 6) | -8(-31.1 to 31.2) |
| Republic of Zimbabwe | 47(35 to 63) | 57(40 to 80) | 21.1(-5.8 to 54.5) | 5.4(3.9 to 7.2) | 5.2(3.6 to 7.2) | -3.6(-25.1 to 22.9) |
| Republic of Benin | 1(1 to 1) | 2(1 to 2) | 67.8(20.4 to 119.9) | 0.2(0.1 to 0.2) | 0.2(0.1 to 0.2) | 11.8(-19.7 to 46.6) |
| Burkina Faso | 2(1 to 3) | 3(2 to 4) | 71.7(21.3 to 123.4) | 0.2(0.1 to 0.2) | 0.2(0.1 to 0.3) | 25.6(-11.3 to 63.4) |
| Republic of Cameroon | 3(2 to 3) | 4(2 to 6) | 68.4(15.5 to 133.8) | 0.2(0.1 to 0.3) | 0.2(0.1 to 0.3) | 11.8(-23.4 to 55.2) |
| Republic of Cabo Verde | 1(0 to 1) | 1(0 to 2) | 38.2(0.6 to 76.6) | 1.9(0.7 to 2.7) | 1.7(0.6 to 2.3) | -10.7(-35 to 14.1) |
| Republic of Chad | 1(1 to 2) | 2(1 to 2) | 55.2(12.3 to 105.6) | 0.2(0.1 to 0.2) | 0.2(0.1 to 0.2) | 12.2(-18.8 to 48.6) |
| Republic of C么te d'Ivoire | 2(1 to 3) | 3(2 to 5) | 75.5(21.8 to 128.3) | 0.2(0.1 to 0.2) | 0.2(0.1 to 0.3) | 19.1(-17.4 to 54.9) |
| Republic of the Gambia | 0(0 to 0) | 0(0 to 0) | 34.9(8.9 to 68.2) | 0(0 to 0) | 0(0 to 0) | 0.9(-18.5 to 25.8) |
| Republic of Ghana | 3(2 to 4) | 6(3 to 8) | 90.2(33.8 to 153.5) | 0.2(0.1 to 0.2) | 0.2(0.1 to 0.3) | 32.5(-6.8 to 76.6) |
| Republic of Guinea | 1(1 to 2) | 2(1 to 3) | 27.4(-5 to 65) | 0.2(0.1 to 0.3) | 0.2(0.1 to 0.3) | 9.1(-18.6 to 41.3) |
| Republic of Guinea-Bissau | 0(0 to 0) | 0(0 to 0) | 53.4(14.2 to 100) | 0.2(0.1 to 0.3) | 0.2(0.1 to 0.3) | 16.1(-13.6 to 51.4) |
| Republic of Liberia | 0(0 to 1) | 1(0 to 1) | 49.1(9.9 to 92.3) | 0.2(0.1 to 0.2) | 0.2(0.1 to 0.3) | 6.3(-21.6 to 37.1) |
| Republic of Mali | 11(9 to 17) | 15(11 to 24) | 37.2(8.6 to 67.7) | 1.1(0.8 to 1.6) | 1.1(0.8 to 1.6) | -1.8(-22.2 to 20) |
| Islamic Republic of Mauritania | 0(0 to 1) | 1(0 to 1) | 72.7(25.4 to 124.4) | 0.2(0.1 to 0.2) | 0.2(0.1 to 0.3) | 21.7(-11.7 to 58.1) |
| Republic of the Niger | 1(1 to 2) | 2(1 to 3) | 109.6(46.3 to 181.8) | 0.1(0.1 to 0.2) | 0.2(0.1 to 0.2) | 32.9(-7.3 to 78.6) |
| Federal Republic of Nigeria | 19(14 to 26) | 28(19 to 39) | 51.8(10.3 to 112.5) | 0.2(0.1 to 0.2) | 0.2(0.1 to 0.3) | 4.8(-23.8 to 46.7) |
| Democratic Republic of Sao Tome and Principe | 1(1 to 1) | 1(1 to 2) | 29.2(4.3 to 65.2) | 7.6(4.9 to 10.1) | 6.7(4.3 to 8.7) | -11(-28.2 to 13.8) |
| Republic of Senegal | 2(1 to 2) | 3(2 to 4) | 70.4(28.5 to 118.3) | 0.2(0.1 to 0.2) | 0.2(0.1 to 0.3) | 23.3(-7 to 57.9) |
| Republic of Sierra Leone | 1(1 to 1) | 1(1 to 2) | 61.1(15.5 to 109.3) | 0.2(0.1 to 0.2) | 0.2(0.1 to 0.3) | 10.7(-20.6 to 43.8) |
| Togolese Republic | 1(0 to 1) | 1(1 to 2) | 98(38.8 to 170.9) | 0.2(0.1 to 0.2) | 0.2(0.1 to 0.3) | 25.2(-12.2 to 71.3) |
| American Samoa | 0(0 to 0) | 0(0 to 0) | -4.7(-28.6 to 35.8) | 3(2.2 to 3.7) | 2.1(1.6 to 3.3) | -29(-46.8 to 1.2) |
| Bermuda | 1(1 to 1) | 1(1 to 1) | 17.3(0 to 44.9) | 4.8(4.2 to 5.4) | 4.2(3.5 to 5.1) | -13.2(-26.1 to 7.1) |
| Cook Islands | 0(0 to 0) | 0(0 to 0) | 31.6(-5.7 to 78.8) | 2.7(2 to 3.3) | 2.6(1.7 to 3.9) | -4(-31.2 to 30.4) |
| Greenland | 1(1 to 1) | 1(1 to 1) | 19.8(-8.3 to 54) | 9.1(6.7 to 12.1) | 7.4(5.4 to 10.2) | -19(-38.1 to 4.1) |
| Guam | 0(0 to 1) | 0(0 to 1) | 7.4(-19.2 to 42.5) | 1.7(1.2 to 2.1) | 1.3(1 to 1.8) | -26.2(-44.4 to -2) |
| Principality of Monaco | 1(1 to 1) | 1(1 to 1) | 12.6(-9.5 to 41.6) | 5.9(4.2 to 7.8) | 5.8(4.2 to 7.9) | -2(-21.2 to 23.3) |
| Republic of Nauru | 0(0 to 0) | 0(0 to 0) | 6.6(-15.2 to 36.9) | 4(2.3 to 5.6) | 3.7(2.2 to 5.1) | -7.8(-26.7 to 18.4) |
| Republic of Niue | 0(0 to 0) | 0(0 to 0) | 2.6(-15.6 to 22.5) | 3.3(2.2 to 4) | 3(2.1 to 3.8) | -8.6(-24.8 to 9.1) |
| Northern Mariana Islands | 0(0 to 0) | 0(0 to 0) | 15.5(-13.5 to 78) | 2.4(1.7 to 3.1) | 1.7(1.4 to 3.1) | -28.3(-46.3 to 10.5) |
| Republic of Palau | 0(0 to 0) | 0(0 to 0) | 40.5(5.9 to 85.7) | 2.1(1.4 to 2.7) | 1.9(1.2 to 2.7) | -8.4(-31 to 21.1) |
| Puerto Rico | 45(42 to 49) | 49(40 to 57) | 7.7(-12.1 to 26.8) | 4.5(4.2 to 4.9) | 4.2(3.4 to 4.9) | -8(-24.9 to 8.3) |
| Saint Kitts and Nevis | 0(0 to 1) | 1(0 to 1) | 17.3(-3.5 to 38.2) | 6.2(5.7 to 6.7) | 4.3(3.5 to 5.1) | -30.8(-43.1 to -18.4) |
| Republic of San Marino | 2(1 to 3) | 2(1 to 2) | -23.8(-45.9 to 8.4) | 21.6(15.7 to 29.4) | 12.9(8.3 to 18.8) | -40.2(-57.6 to -14.9) |
| Tokelau | 0(0 to 0) | 0(0 to 0) | -6.7(-25.3 to 13.6) | 3.2(2.2 to 4.2) | 2.8(1.9 to 3.8) | -13.5(-30.7 to 5.3) |
| Tuvalu | 0(0 to 0) | 0(0 to 0) | 14.5(-5.9 to 38.8) | 2.9(1.9 to 3.8) | 2.7(1.9 to 3.5) | -7.5(-24 to 12.1) |
| United States Virgin Islands | 1(1 to 2) | 1(1 to 2) | 8.5(-15.3 to 43.5) | 4.7(3.6 to 6.5) | 4.6(3.3 to 6.6) | -2.3(-23.8 to 29.2) |
| Republic of South Sudan | 15(9 to 21) | 21(13 to 31) | 40.9(8.3 to 84.3) | 3.2(2 to 4.6) | 3.4(2.1 to 4.9) | 5.6(-18.9 to 38.1) |
| Republic of Sudan | 90(63 to 138) | 123(86 to 180) | 37.3(6.1 to 76.9) | 4(2.8 to 6.2) | 4(2.8 to 5.8) | -1.2(-23.6 to 27.3) |

* GBTC represents gallbladder and biliary tract cancer, ASR represents age-standardized rate.

**Table** 2-**S2. The deaths of GBTC between 2010 and 2021 at national level, both sexes in aged 55 years and older**

|  | | | | | | |
| --- | --- | --- | --- | --- | --- | --- |
| region | Case in 2010 | Case in 2021 | Change in number (95% UI) | ASR in 2010 | ASR in 2021 | change in ASR per 100 000 population (95% UI) |
| People's Republic of China | 24592(17484 to 28526) | 33806(23681 to 43755) | 37.5(11.6 to 69) | 9.5(6.8 to 11) | 8.9(6.2 to 11.5) | -6.3(-24 to 15.1) |
| Democratic People's Republic of Korea | 345(239 to 602) | 382(258 to 693) | 10.8(-10.1 to 35.6) | 8(5.5 to 14) | 6.8(4.6 to 12.3) | -15.2(-31.2 to 3.8) |
| Taiwan (Province of China) | 684(626 to 725) | 838(744 to 923) | 22.6(10.5 to 33.8) | 13.4(12.3 to 14.2) | 11.1(9.9 to 12.3) | -16.9(-25.1 to -9.3) |
| Kingdom of Cambodia | 60(41 to 126) | 105(67 to 226) | 74.8(26 to 145.2) | 4.4(3 to 9.2) | 4.8(3.1 to 10.4) | 9.6(-20.9 to 53.8) |
| Republic of Indonesia | 911(619 to 1970) | 1294(843 to 2838) | 42.1(12.4 to 71.2) | 3.4(2.3 to 7.3) | 3.1(2 to 6.8) | -8.5(-27.7 to 10.2) |
| Lao People's Democratic Republic | 21(13 to 46) | 30(19 to 67) | 42.1(6.6 to 83.3) | 4.1(2.5 to 8.8) | 3.9(2.5 to 8.7) | -4.3(-28.2 to 23.5) |
| Malaysia | 161(108 to 198) | 259(167 to 318) | 60.6(35.5 to 91.7) | 5(3.3 to 6.1) | 5.3(3.4 to 6.5) | 6(-10.6 to 26.5) |
| Republic of Maldives | 1(1 to 1) | 1(1 to 2) | 32.1(3.7 to 66.4) | 3.6(2.3 to 4.2) | 2.6(1.7 to 3.3) | -26.9(-42.7 to -8) |
| Republic of the Union of Myanmar | 202(127 to 417) | 281(184 to 597) | 39.5(8.4 to 81) | 3.5(2.2 to 7.2) | 3.3(2.2 to 7.1) | -4.2(-25.6 to 24.3) |
| Republic of the Philippines | 211(180 to 379) | 324(254 to 582) | 53.6(28.4 to 81.3) | 2.3(1.9 to 4.1) | 2.3(1.8 to 4.2) | 2.4(-14.4 to 20.8) |
| Democratic Socialist Republic of Sri Lanka | 135(108 to 248) | 178(109 to 323) | 31.6(-12.3 to 78.7) | 4(3.2 to 7.4) | 3.7(2.3 to 6.7) | -7.9(-38.6 to 25.1) |
| Kingdom of Thailand | 3702(2115 to 4511) | 6184(3115 to 8338) | 67(20 to 126) | 30.3(17.3 to 36.9) | 31.8(16 to 42.9) | 5(-24.5 to 42.1) |
| Democratic Republic of Timor-Leste | 3(2 to 7) | 5(3 to 10) | 47(16.6 to 81.8) | 2.9(2 to 6.3) | 3.3(2.1 to 7.2) | 15(-8.8 to 42.2) |
| Socialist Republic of Viet Nam | 650(512 to 877) | 1001(683 to 1470) | 53.9(16.6 to 102.2) | 6.1(4.8 to 8.2) | 5.7(3.9 to 8.4) | -5.5(-28.5 to 24.1) |
| Republic of Fiji | 5(3 to 6) | 7(4 to 9) | 31.6(-2.2 to 71.7) | 5(3.1 to 5.9) | 4.8(2.8 to 6.5) | -2.6(-27.6 to 27.1) |
| Republic of Kiribati | 0(0 to 0) | 0(0 to 0) | 32.6(7.3 to 63.6) | 2.8(1.4 to 3.7) | 2.5(1.3 to 3.4) | -9.8(-27 to 11.3) |
| Republic of the Marshall Islands | 0(0 to 0) | 0(0 to 0) | 37.4(10.3 to 72.2) | 2.8(1.8 to 3.9) | 2.7(1.7 to 3.7) | -6.5(-24.9 to 17.3) |
| Federated States of Micronesia | 0(0 to 0) | 0(0 to 1) | 22.8(-2.5 to 53.1) | 3.1(2.1 to 4.2) | 2.8(1.8 to 3.8) | -11.6(-29.8 to 10.1) |
| Independent State of Papua New Guinea | 11(7 to 15) | 16(11 to 23) | 50(15.4 to 92.6) | 2.2(1.4 to 3) | 2(1.3 to 2.8) | -8.1(-29.2 to 18) |
| Independent State of Samoa | 1(0 to 1) | 1(0 to 1) | 15.1(-6.8 to 40.5) | 3.2(2.1 to 4.2) | 2.9(2 to 3.9) | -9.6(-26.7 to 10.4) |
| Solomon Islands | 1(1 to 1) | 1(1 to 2) | 39.5(9.8 to 75.3) | 2.6(1.6 to 3.7) | 2.6(1.7 to 3.8) | 0.9(-20.6 to 26.8) |
| Kingdom of Tonga | 0(0 to 0) | 0(0 to 0) | 4(-16.6 to 26) | 2.7(1.8 to 3.7) | 2.4(1.7 to 3.5) | -8.6(-26.7 to 10.8) |
| Republic of Vanuatu | 0(0 to 1) | 1(0 to 1) | 45.3(20.1 to 75) | 2.4(1.6 to 3.3) | 2.4(1.6 to 3.2) | -1.2(-18.3 to 19) |
| Republic of Armenia | 25(21 to 28) | 66(55 to 77) | 166.4(113.3 to 231) | 4(3.4 to 4.6) | 8.4(7 to 9.8) | 110.2(68.3 to 161.2) |
| Republic of Azerbaijan | 46(30 to 68) | 58(34 to 87) | 25.1(-21.4 to 92.5) | 4.2(2.7 to 6.2) | 3(1.8 to 4.5) | -28.4(-55 to 10.1) |
| Georgia | 49(41 to 58) | 89(73 to 107) | 81.8(45.3 to 126.6) | 4.9(4.1 to 5.8) | 8.5(6.9 to 10.2) | 74.2(39.2 to 117) |
| Republic of Kazakhstan | 154(139 to 169) | 159(131 to 189) | 3.6(-16.1 to 23.8) | 6.7(6 to 7.3) | 5(4.1 to 5.9) | -24.8(-39 to -10.1) |
| Kyrgyz Republic | 22(18 to 25) | 41(33 to 49) | 86.2(47.4 to 134.1) | 4(3.4 to 4.6) | 4.8(3.9 to 5.8) | 19.4(-5.5 to 50.1) |
| Mongolia | 32(23 to 48) | 48(35 to 73) | 50.4(0.9 to 111.6) | 14.6(10.6 to 22) | 12.3(8.8 to 18.5) | -16(-43.6 to 18.2) |
| Republic of Tajikistan | 4(3 to 6) | 5(4 to 7) | 25.2(-14.1 to 86.1) | 0.8(0.5 to 1.1) | 0.5(0.3 to 0.7) | -34.1(-54.8 to -2) |
| Turkmenistan | 12(10 to 13) | 25(20 to 33) | 117.1(65.5 to 189.5) | 2.6(2.3 to 3) | 3.6(2.8 to 4.7) | 35.9(3.6 to 81.2) |
| Republic of Uzbekistan | 30(24 to 38) | 81(63 to 104) | 166.2(97.3 to 262.1) | 1.1(0.9 to 1.4) | 1.7(1.4 to 2.2) | 56.8(16.3 to 113.3) |
| Republic of Albania | 39(26 to 59) | 54(36 to 79) | 38.5(-8.1 to 104.6) | 6.5(4.3 to 9.9) | 6.9(4.6 to 10) | 5.3(-30.1 to 55.6) |
| Bosnia and Herzegovina | 186(150 to 307) | 188(138 to 316) | 0.8(-23.4 to 28.8) | 18.7(15 to 30.8) | 17.1(12.6 to 28.8) | -8.3(-30.3 to 17.2) |
| Republic of Bulgaria | 210(192 to 226) | 184(155 to 218) | -12.2(-26.2 to 4.2) | 8.8(8 to 9.4) | 7.8(6.5 to 9.2) | -11.5(-25.6 to 5) |
| Republic of Croatia | 241(217 to 265) | 250(213 to 289) | 3.7(-12.8 to 21.6) | 17.9(16.2 to 19.7) | 16.8(14.3 to 19.4) | -6.5(-21.4 to 9.6) |
| Czech Republic | 797(680 to 884) | 788(662 to 925) | -1.2(-17.3 to 18.5) | 25.4(21.6 to 28.1) | 22.5(18.9 to 26.5) | -11.1(-25.5 to 6.6) |
| Hungary | 658(611 to 705) | 557(488 to 626) | -15.4(-25.1 to -4.2) | 21.6(20.1 to 23.1) | 17.5(15.3 to 19.7) | -18.9(-28.3 to -8.2) |
| North Macedonia | 67(49 to 81) | 72(53 to 92) | 8.2(-15 to 33.4) | 13.8(10.2 to 16.7) | 12.1(8.9 to 15.6) | -11.8(-30.7 to 8.8) |
| Montenegro | 13(9 to 17) | 14(10 to 19) | 10.5(-17 to 46.2) | 8.3(6.1 to 10.9) | 8.2(5.9 to 10.9) | -2.1(-26.4 to 29.6) |
| Republic of Poland | 2110(1962 to 2199) | 2125(1914 to 2303) | 0.7(-6.9 to 8.6) | 20.5(19.1 to 21.4) | 17.5(15.8 to 19) | -14.5(-20.9 to -7.8) |
| Romania | 525(486 to 564) | 543(473 to 616) | 3.3(-10.9 to 17.8) | 8.9(8.3 to 9.6) | 9(7.9 to 10.3) | 1(-12.9 to 15.2) |
| Republic of Serbia | 346(259 to 442) | 359(252 to 479) | 3.8(-21.8 to 34.2) | 13.3(10 to 17) | 12.8(9 to 17.1) | -4(-27.7 to 24.1) |
| Slovak Republic | 292(228 to 373) | 343(247 to 465) | 17.5(-12.1 to 50.4) | 21.6(16.9 to 27.6) | 20.9(15.1 to 28.3) | -3.1(-27.5 to 24.1) |
| Republic of Slovenia | 109(98 to 119) | 108(92 to 126) | -0.2(-14.6 to 15.4) | 18.1(16.2 to 19.8) | 14.8(12.6 to 17.2) | -18(-29.8 to -5.2) |
| Republic of Belarus | 198(177 to 217) | 204(165 to 247) | 2.8(-15.4 to 27.4) | 8.1(7.2 to 8.8) | 7.1(5.8 to 8.6) | -12.3(-27.8 to 8.7) |
| Republic of Estonia | 38(34 to 41) | 47(41 to 54) | 25.4(9.9 to 43.5) | 9.6(8.6 to 10.4) | 10.8(9.3 to 12.3) | 13.2(-0.8 to 29.5) |
| Republic of Latvia | 46(42 to 50) | 49(43 to 56) | 8.1(-5 to 23.6) | 7.2(6.6 to 7.8) | 7.5(6.5 to 8.6) | 4.1(-8.5 to 19.1) |
| Republic of Lithuania | 80(73 to 87) | 107(93 to 121) | 32.8(15 to 50.2) | 9.1(8.2 to 9.9) | 11(9.6 to 12.5) | 21.5(5.1 to 37.4) |
| Republic of Moldova | 44(42 to 47) | 48(43 to 53) | 7.7(-4.1 to 21) | 5(4.7 to 5.3) | 4.5(4.1 to 5.1) | -9.7(-19.6 to 1.5) |
| Russian Federation | 2659(2530 to 2736) | 3190(2921 to 3449) | 20(11.1 to 29.2) | 7.4(7 to 7.6) | 7.5(6.9 to 8.1) | 1.5(-6 to 9.4) |
| Ukraine | 829(778 to 877) | 717(538 to 927) | -13.5(-34.6 to 12.9) | 6.7(6.3 to 7.1) | 5.3(4 to 6.8) | -21(-40.3 to 3.2) |
| Brunei Darussalam | 4(3 to 5) | 6(5 to 8) | 44.3(21.1 to 74.6) | 12.7(9.5 to 15.7) | 10.5(8.1 to 13.5) | -17.3(-30.6 to 0.1) |
| Japan | 19274(16287 to 20883) | 21786(17687 to 24178) | 13(7.4 to 16.7) | 39.6(33.5 to 42.9) | 41.7(33.9 to 46.3) | 5.4(0.1 to 8.8) |
| Republic of Korea | 3558(2527 to 4291) | 5364(3322 to 6829) | 50.8(22.7 to 80.9) | 34(24.2 to 41) | 32.1(19.9 to 40.8) | -5.8(-23.3 to 13.1) |
| Republic of Singapore | 55(50 to 59) | 79(70 to 86) | 44.2(31.6 to 57.4) | 6.3(5.8 to 6.8) | 5.2(4.6 to 5.7) | -17.3(-24.5 to -9.7) |
| Australia | 402(355 to 432) | 458(397 to 498) | 14(7.5 to 20.9) | 7.4(6.5 to 8) | 6.2(5.4 to 6.7) | -16.5(-21.3 to -11.5) |
| New Zealand | 73(65 to 79) | 133(117 to 145) | 82.6(70 to 96.1) | 7(6.2 to 7.5) | 9.3(8.2 to 10.2) | 34.3(25 to 44.2) |
| Principality of Andorra | 3(2 to 4) | 3(2 to 4) | -4.7(-28.5 to 27.6) | 13.8(10 to 18.7) | 9.9(6.7 to 13.6) | -28.2(-46.1 to -3.9) |
| Republic of Austria | 328(293 to 352) | 314(273 to 343) | -4.3(-11.4 to 3) | 13.4(12 to 14.4) | 10.6(9.2 to 11.6) | -20.8(-26.7 to -14.7) |
| Kingdom of Belgium | 245(215 to 267) | 208(177 to 231) | -15.2(-21.3 to -8.6) | 7.7(6.7 to 8.3) | 5.5(4.7 to 6.1) | -28.2(-33.4 to -22.6) |
| Republic of Cyprus | 24(18 to 30) | 27(20 to 36) | 10.9(-10.4 to 38.4) | 9.9(7.5 to 12.2) | 7.6(5.6 to 10.2) | -22.6(-37.5 to -3.4) |
| Kingdom of Denmark | 121(110 to 128) | 127(113 to 139) | 5.5(-1.8 to 13.5) | 7.4(6.7 to 7.9) | 6.6(5.9 to 7.2) | -10.7(-16.9 to -3.9) |
| Republic of Finland | 196(173 to 212) | 244(210 to 270) | 24.9(13.3 to 34.8) | 11.3(10 to 12.2) | 12.1(10.4 to 13.4) | 7.5(-2.4 to 16.1) |
| French Republic | 1599(1386 to 1723) | 1510(1281 to 1708) | -5.5(-13.5 to 3) | 8.6(7.5 to 9.3) | 6.8(5.8 to 7.7) | -20.7(-27.4 to -13.6) |
| Federal Republic of Germany | 3666(3271 to 3922) | 4116(3556 to 4491) | 12.3(5.2 to 19.3) | 13.7(12.2 to 14.6) | 13.1(11.3 to 14.3) | -4.4(-10.4 to 1.6) |
| Hellenic Republic | 505(461 to 540) | 344(307 to 371) | -31.8(-36.3 to -26.8) | 14.9(13.6 to 16) | 9.4(8.4 to 10.1) | -37.3(-41.4 to -32.7) |
| Republic of Iceland | 5(5 to 6) | 6(5 to 6) | 10.1(-1.3 to 21.2) | 7(6.3 to 7.7) | 5.7(4.9 to 6.4) | -18.9(-27.3 to -10.7) |
| Ireland | 58(52 to 63) | 59(50 to 65) | 1.8(-7.1 to 10.7) | 5.9(5.3 to 6.5) | 4.5(3.8 to 5) | -24.1(-30.8 to -17.5) |
| State of Israel | 99(88 to 108) | 101(88 to 113) | 1.9(-7.6 to 11.3) | 6.7(5.9 to 7.3) | 5.1(4.4 to 5.6) | -24.3(-31.4 to -17.3) |
| Republic of Italy | 3822(3333 to 4104) | 3889(3346 to 4244) | 1.8(-1.9 to 5.4) | 19.5(17 to 20.9) | 17.1(14.7 to 18.7) | -12.2(-15.4 to -9.1) |
| Grand Duchy of Luxembourg | 9(8 to 10) | 10(9 to 12) | 12.7(1.8 to 25.3) | 7.4(6.8 to 8) | 5.9(5.2 to 6.6) | -20.6(-28.3 to -11.8) |
| Republic of Malta | 7(6 to 7) | 7(6 to 8) | 13.1(0.7 to 25.3) | 5.2(4.6 to 5.6) | 4.7(4 to 5.2) | -9.4(-19.3 to 0.4) |
| Kingdom of the Netherlands | 426(380 to 455) | 502(440 to 552) | 17.8(7.9 to 26.8) | 9.1(8.1 to 9.7) | 8.6(7.5 to 9.4) | -5.3(-13.3 to 2) |
| Kingdom of Norway | 84(75 to 89) | 97(85 to 105) | 15(10.4 to 19.8) | 6.4(5.7 to 6.8) | 6(5.2 to 6.4) | -6.8(-10.6 to -2.9) |
| Portuguese Republic | 330(295 to 354) | 359(313 to 395) | 8.8(1.1 to 17.5) | 10(8.9 to 10.7) | 9.2(8 to 10.1) | -7.6(-14.2 to -0.2) |
| Kingdom of Spain | 1329(1160 to 1448) | 1418(1195 to 1576) | 6.7(-1.4 to 14.4) | 10.2(8.9 to 11.1) | 9.2(7.7 to 10.2) | -10.3(-17.1 to -3.9) |
| Kingdom of Sweden | 459(410 to 495) | 573(492 to 642) | 24.8(12.7 to 37.9) | 15.6(14 to 16.8) | 17(14.6 to 19.1) | 8.9(-1.6 to 20.4) |
| Swiss Confederation | 157(136 to 173) | 201(169 to 229) | 28(17.4 to 39.1) | 6.8(5.9 to 7.5) | 6.8(5.8 to 7.8) | 0.4(-7.9 to 9.1) |
| United Kingdom of Great Britain and Northern Ireland | 978(887 to 1027) | 1376(1215 to 1456) | 40.7(36.7 to 44.1) | 5.5(5 to 5.8) | 6.5(5.8 to 6.9) | 18.4(15 to 21.2) |
| Argentine Republic | 1733(1612 to 1840) | 1594(1456 to 1706) | -8(-13.9 to -0.8) | 22.5(20.9 to 23.9) | 17.1(15.6 to 18.3) | -24(-28.9 to -18.1) |
| Republic of Chile | 1778(1646 to 1887) | 1809(1627 to 1979) | 1.7(-5.5 to 9.9) | 58.5(54.2 to 62.1) | 40.4(36.3 to 44.2) | -31(-35.9 to -25.4) |
| Eastern Republic of Uruguay | 205(186 to 220) | 215(194 to 236) | 4.6(-4.1 to 14.2) | 26.1(23.7 to 27.9) | 23.7(21.4 to 26.1) | -8.9(-16.5 to -0.6) |
| Canada | 686(612 to 737) | 708(610 to 772) | 3.1(-4.6 to 11.5) | 7.6(6.7 to 8.1) | 5.8(5 to 6.3) | -23.7(-29.4 to -17.5) |
| United States of America | 3956(3532 to 4174) | 4902(4397 to 5194) | 23.9(21.2 to 26.7) | 5.2(4.6 to 5.5) | 4.9(4.4 to 5.2) | -5.7(-7.8 to -3.6) |
| Antigua and Barbuda | 1(1 to 1) | 1(1 to 1) | 15.5(6.3 to 25.3) | 7.1(6.6 to 7.6) | 5.5(5.2 to 5.9) | -22.7(-28.9 to -16.1) |
| Commonwealth of the Bahamas | 4(3 to 4) | 5(4 to 6) | 30.6(7.6 to 62.2) | 7.9(7.3 to 8.5) | 6.7(5.5 to 8.1) | -14.9(-29.9 to 5.7) |
| Barbados | 5(5 to 5) | 6(5 to 8) | 26.9(-1.2 to 55.6) | 7.7(7.2 to 8.2) | 7(5.5 to 8.6) | -9.6(-29.6 to 10.9) |
| Belize | 2(2 to 2) | 2(2 to 3) | 25.6(10.4 to 42.4) | 6.7(6.3 to 7.1) | 4.8(4.2 to 5.4) | -28.2(-36.9 to -18.6) |
| Republic of Cuba | 166(155 to 176) | 167(144 to 188) | 0.3(-13.4 to 13.9) | 6.4(5.9 to 6.7) | 4.9(4.2 to 5.5) | -23.1(-33.6 to -12.7) |
| Commonwealth of Dominica | 1(1 to 1) | 1(1 to 2) | 11.3(-8 to 37) | 9.8(7.3 to 12.6) | 8.3(6.1 to 10.4) | -15.2(-30 to 4.3) |
| Dominican Republic | 50(38 to 72) | 70(53 to 101) | 41(6.6 to 81.9) | 4.1(3.2 to 6) | 4.2(3.1 to 6) | 1.3(-23.3 to 30.8) |
| Grenada | 1(1 to 1) | 1(1 to 2) | 5.9(-8 to 20.8) | 7.9(7.3 to 8.5) | 6.7(5.8 to 7.7) | -14.6(-25.9 to -2.6) |
| Republic of Guyana | 5(4 to 5) | 6(4 to 7) | 20.3(-5.2 to 50.6) | 5.7(5.2 to 6.2) | 5(3.9 to 6.3) | -11.9(-30.6 to 10.3) |
| Republic of Haiti | 67(39 to 100) | 83(52 to 126) | 24.1(-1.3 to 60.3) | 7.8(4.6 to 11.6) | 7(4.4 to 10.6) | -10.2(-28.6 to 16.1) |
| Jamaica | 24(22 to 26) | 29(23 to 37) | 20.4(-6.9 to 53.7) | 5.9(5.4 to 6.4) | 5.6(4.3 to 7) | -6.5(-27.7 to 19.3) |
| Saint Lucia | 1(1 to 1) | 2(1 to 2) | 52.9(24.6 to 85.2) | 4.2(3.9 to 4.5) | 4.1(3.3 to 4.9) | -3.6(-21.5 to 16.7) |
| Saint Vincent and the Grenadines | 1(1 to 1) | 1(1 to 1) | 14.7(-0.1 to 32.1) | 5.4(5.1 to 5.8) | 4.2(3.8 to 4.8) | -21.9(-32 to -10) |
| Republic of Suriname | 4(3 to 5) | 5(4 to 7) | 27.6(-5.6 to 67) | 5.3(4.3 to 7.2) | 4.4(3.2 to 6.1) | -17(-38.6 to 8.6) |
| Republic of Trinidad and Tobago | 12(11 to 13) | 17(13 to 22) | 40.4(9.4 to 77.5) | 5(4.7 to 5.3) | 4.9(3.9 to 6.2) | -1.7(-23.5 to 24.2) |
| Plurinational State of Bolivia | 351(226 to 488) | 447(289 to 636) | 27.1(2.3 to 61.6) | 31.7(20.3 to 43.9) | 28.9(18.7 to 41.2) | -8.7(-26.5 to 16.1) |
| Republic of Ecuador | 379(344 to 416) | 435(347 to 538) | 14.7(-9.8 to 45.7) | 20.3(18.4 to 22.3) | 15.7(12.5 to 19.4) | -22.5(-39 to -1.6) |
| Republic of Peru | 804(582 to 996) | 1177(821 to 1642) | 46.4(5.8 to 97.9) | 21(15.2 to 26) | 21(14.7 to 29.4) | 0.2(-27.6 to 35.5) |
| Republic of Colombia | 846(781 to 907) | 1180(983 to 1381) | 39.5(17.4 to 62.5) | 13.3(12.3 to 14.3) | 12.3(10.3 to 14.4) | -7.2(-21.9 to 8.1) |
| Republic of Costa Rica | 65(59 to 70) | 91(80 to 103) | 40.6(23.4 to 58.3) | 10.5(9.6 to 11.3) | 9.6(8.4 to 10.8) | -9.4(-20.5 to 2) |
| Republic of El Salvador | 101(82 to 143) | 137(105 to 194) | 35.2(9.2 to 65.3) | 12.6(10.3 to 17.9) | 13.4(10.3 to 19) | 5.8(-14.6 to 29.3) |
| Republic of Guatemala | 136(129 to 142) | 148(129 to 170) | 9(-6.4 to 26.2) | 11(10.4 to 11.5) | 8.1(7 to 9.3) | -26.6(-36.9 to -15) |
| Republic of Honduras | 112(77 to 150) | 173(118 to 232) | 55.3(18.2 to 99.8) | 15.7(10.8 to 21.1) | 16.5(11.3 to 22.1) | 4.8(-20.2 to 34.8) |
| United Mexican States | 1816(1738 to 1862) | 2324(2058 to 2601) | 28(14.2 to 41.8) | 12.8(12.3 to 13.1) | 10.8(9.5 to 12.1) | -15.9(-24.9 to -6.8) |
| Republic of Nicaragua | 69(56 to 93) | 85(66 to 133) | 24.2(-0.1 to 54) | 12.8(10.5 to 17.4) | 10.5(8.2 to 16.4) | -18.5(-34.4 to 1.1) |
| Republic of Panama | 35(32 to 38) | 47(37 to 56) | 32.6(4.2 to 58.1) | 7.1(6.5 to 7.6) | 6.3(5 to 7.5) | -11.4(-30.4 to 5.7) |
| Bolivarian Republic of Venezuela | 279(261 to 294) | 416(313 to 529) | 48.7(11.1 to 89.1) | 8(7.5 to 8.4) | 7.9(6 to 10.1) | -1(-26 to 25.9) |
| Federative Republic of Brazil | 3509(3237 to 3673) | 4983(4510 to 5262) | 42(37.3 to 46.8) | 12(11.1 to 12.6) | 11.5(10.4 to 12.2) | -4.1(-7.3 to -0.9) |
| Republic of Paraguay | 68(50 to 87) | 95(66 to 131) | 38.3(4.9 to 78.1) | 10(7.2 to 12.7) | 9.6(6.7 to 13.2) | -4.2(-27.3 to 23.5) |
| People's Democratic Republic of Algeria | 590(428 to 785) | 861(617 to 1119) | 45.9(18.5 to 77.3) | 15.2(11 to 20.2) | 14.2(10.2 to 18.4) | -6.7(-24.2 to 13.4) |
| Kingdom of Bahrain | 3(2 to 3) | 5(3 to 7) | 74.3(30.7 to 136.9) | 3.6(2.4 to 4.3) | 2.9(1.9 to 4.1) | -18.2(-38.7 to 11.1) |
| Arab Republic of Egypt | 476(343 to 563) | 640(450 to 795) | 34.4(7.8 to 66.7) | 6.3(4.6 to 7.5) | 5.8(4.1 to 7.2) | -8.4(-26.6 to 13.6) |
| Islamic Republic of Iran | 368(204 to 429) | 500(275 to 580) | 35.9(25.9 to 47.2) | 4.4(2.4 to 5.1) | 3.8(2.1 to 4.5) | -11.6(-18.1 to -4.2) |
| Republic of Iraq | 106(76 to 143) | 181(125 to 233) | 70.1(26.2 to 121.9) | 4.3(3.1 to 5.8) | 4.7(3.2 to 6) | 7.5(-20.3 to 40.2) |
| Hashemite Kingdom of Jordan | 36(25 to 45) | 64(44 to 89) | 75.9(35.4 to 131.3) | 6.9(4.7 to 8.5) | 5.1(3.5 to 7.1) | -26.1(-43.1 to -2.9) |
| State of Kuwait | 13(12 to 15) | 20(16 to 24) | 49.8(24.2 to 79.5) | 6.5(5.9 to 7.1) | 4.3(3.5 to 5.2) | -33.3(-44.7 to -20.1) |
| Lebanese Republic | 70(42 to 88) | 82(50 to 108) | 17.6(-5.3 to 44.8) | 9.7(5.9 to 12.2) | 8.3(5.1 to 11) | -13.7(-30.5 to 6.3) |
| State of Libya | 100(73 to 122) | 136(91 to 183) | 36.4(5.2 to 73.9) | 19.3(14.1 to 23.7) | 16.3(10.9 to 21.9) | -15.5(-34.8 to 7.6) |
| Kingdom of Morocco | 136(83 to 182) | 197(114 to 251) | 45.5(12.1 to 86) | 3.4(2.1 to 4.6) | 3.3(1.9 to 4.2) | -3.4(-25.6 to 23.5) |
| Palestine | 20(13 to 24) | 24(16 to 31) | 22.2(-1 to 53.1) | 7.7(4.9 to 9.3) | 5.7(3.6 to 7.1) | -26.4(-40.3 to -7.7) |
| Sultanate of Oman | 7(5 to 9) | 9(6 to 12) | 22.4(-4.1 to 55.2) | 4.4(3.1 to 5.5) | 2.9(2 to 3.8) | -34.9(-49 to -17.4) |
| State of Qatar | 4(3 to 5) | 6(4 to 9) | 62.9(11.2 to 140.2) | 5.5(4 to 7) | 3.9(2.7 to 6) | -29.3(-51.8 to 4.2) |
| Kingdom of Saudi Arabia | 136(110 to 190) | 179(134 to 265) | 31.4(4.7 to 58.9) | 7.7(6.2 to 10.7) | 5.9(4.5 to 8.8) | -22.6(-38.3 to -6.3) |
| Syrian Arab Republic | 6(4 to 8) | 9(5 to 12) | 33(-2.2 to 71.1) | 0.4(0.2 to 0.5) | 0.4(0.2 to 0.5) | -10.9(-34.5 to 14.6) |
| Republic of Tunisia | 177(119 to 237) | 243(162 to 333) | 37.2(3.3 to 85.5) | 11.6(7.8 to 15.5) | 10.4(6.9 to 14.2) | -10.6(-32.6 to 20.9) |
| Republic of Turkey | 887(631 to 1076) | 1093(824 to 1477) | 23.2(-4.1 to 59.1) | 8(5.7 to 9.7) | 6.6(5 to 8.9) | -17.1(-35.5 to 7) |
| United Arab Emirates | 28(20 to 39) | 58(43 to 83) | 106(48 to 201.8) | 14.9(10.4 to 20.4) | 8.1(5.9 to 11.6) | -45.6(-60.9 to -20.3) |
| Republic of Yemen | 72(51 to 109) | 108(70 to 159) | 50.3(16.2 to 91) | 4.7(3.3 to 7.1) | 4.8(3.1 to 7.1) | 3(-20.4 to 30.8) |
| Islamic Republic of Afghanistan | 111(58 to 187) | 112(57 to 173) | 1(-19 to 28.1) | 8.7(4.5 to 14.8) | 9.1(4.7 to 14.1) | 4.2(-16.4 to 32.2) |
| People's Republic of Bangladesh | 1343(941 to 1899) | 2170(1467 to 3342) | 61.6(23.3 to 107.9) | 9.4(6.6 to 13.3) | 9.3(6.3 to 14.3) | -1.6(-25 to 26.5) |
| Kingdom of Bhutan | 8(6 to 11) | 11(8 to 16) | 43.6(17.1 to 75.8) | 10.8(8 to 15.5) | 11.4(7.9 to 16.2) | 6.4(-13.2 to 30.3) |
| Republic of India | 14083(9613 to 16574) | 22728(15624 to 26748) | 61.4(40.3 to 83) | 9.9(6.8 to 11.7) | 11.3(7.8 to 13.3) | 14(-0.9 to 29.2) |
| Federal Democratic Republic of Nepal | 262(195 to 369) | 426(312 to 583) | 62.7(25.9 to 107.4) | 9.1(6.8 to 12.8) | 10.8(7.9 to 14.7) | 18.4(-8.4 to 51) |
| Islamic Republic of Pakistan | 2310(1720 to 3009) | 3087(2237 to 4110) | 33.6(2 to 72) | 16.8(12.5 to 21.9) | 15.6(11.3 to 20.7) | -7.3(-29.3 to 19.4) |
| Republic of Angola | 20(14 to 26) | 33(22 to 45) | 59.6(19 to 111.5) | 1.7(1.2 to 2.2) | 1.7(1.1 to 2.3) | -2.7(-27.4 to 28.9) |
| Central African Republic | 4(3 to 7) | 5(3 to 9) | 25.5(0.8 to 57.5) | 1.7(1 to 2.6) | 1.5(0.9 to 2.5) | -10(-27.7 to 13) |
| Republic of the Congo | 6(4 to 8) | 8(5 to 11) | 46.8(18.3 to 89.9) | 2(1.4 to 2.7) | 1.9(1.2 to 2.5) | -5.2(-23.6 to 22.6) |
| Democratic Republic of the Congo | 55(36 to 86) | 85(53 to 130) | 54.3(21.6 to 97.8) | 1.4(0.9 to 2.2) | 1.4(0.9 to 2.2) | 2.6(-19.1 to 31.6) |
| Republic of Equatorial Guinea | 1(1 to 2) | 1(1 to 2) | 45.7(2.3 to 105.3) | 1.8(1.1 to 2.8) | 1.8(1.1 to 2.7) | -1.4(-30.8 to 38.9) |
| Gabonese Republic | 3(2 to 4) | 3(2 to 5) | 27.3(2.2 to 59.6) | 2.1(1.4 to 2.8) | 1.9(1.2 to 2.6) | -9.6(-27.4 to 13.4) |
| Republic of Burundi | 19(12 to 25) | 28(16 to 40) | 46.5(16.1 to 85.5) | 3.7(2.4 to 5) | 3.5(2 to 5) | -7.1(-26.4 to 17.6) |
| Union of the Comoros | 2(2 to 3) | 3(2 to 5) | 43.9(14.3 to 79.7) | 3.9(2.6 to 5.8) | 4(2.5 to 6.2) | 2.3(-18.8 to 27.7) |
| Republic of Djibouti | 2(1 to 3) | 4(2 to 6) | 72.2(32.3 to 134.6) | 3.5(2 to 5.4) | 3.5(2.1 to 5.7) | -0.6(-23.6 to 35.4) |
| State of Eritrea | 14(9 to 20) | 20(12 to 29) | 44.1(16.6 to 81.4) | 4.5(2.8 to 6.5) | 4.5(2.6 to 6.6) | 0.2(-18.9 to 26.2) |
| Federal Democratic Republic of Ethiopia | 314(212 to 435) | 466(321 to 658) | 48.1(19.6 to 78) | 6.4(4.3 to 8.8) | 6.8(4.7 to 9.6) | 6.7(-13.9 to 28.2) |
| Republic of Kenya | 136(102 to 194) | 203(145 to 290) | 49.3(22.9 to 82.4) | 5.5(4.1 to 7.9) | 5.4(3.9 to 7.8) | -1.5(-18.9 to 20.3) |
| Republic of Madagascar | 34(22 to 47) | 52(32 to 72) | 52.8(17.9 to 93.3) | 2.9(1.9 to 4) | 2.8(1.7 to 3.9) | -2.3(-24.6 to 23.6) |
| Republic of Malawi | 11(7 to 15) | 15(9 to 22) | 33.9(2.3 to 71.2) | 1.3(0.9 to 1.8) | 1.3(0.8 to 1.9) | -2.8(-25.8 to 24.2) |
| Republic of Mauritius | 8(8 to 9) | 13(12 to 14) | 59.1(46.9 to 71.6) | 3.6(3.4 to 3.9) | 3.8(3.5 to 4.1) | 4.8(-3.2 to 13.1) |
| Republic of Mozambique | 59(35 to 85) | 76(43 to 112) | 29.7(-1.7 to 70.8) | 4.2(2.5 to 6.1) | 4.3(2.4 to 6.2) | 0.3(-23.9 to 32.1) |
| Republic of Rwanda | 27(18 to 38) | 46(30 to 64) | 69(31 to 115.8) | 4.2(2.8 to 5.8) | 4.4(2.8 to 6.1) | 4(-19.4 to 32.7) |
| Republic of Seychelles | 1(1 to 1) | 1(1 to 1) | 17.1(-3.4 to 36.7) | 5.6(4.6 to 7.9) | 4.1(3.3 to 6) | -26.2(-39.2 to -13.9) |
| Federal Republic of Somalia | 26(16 to 44) | 35(20 to 59) | 33.6(3.3 to 67.4) | 3.5(2.1 to 5.9) | 3.7(2.1 to 6.2) | 6.3(-17.8 to 33.2) |
| United Republic of Tanzania | 119(80 to 164) | 164(108 to 226) | 38(6.3 to 79.3) | 4.1(2.7 to 5.6) | 4(2.6 to 5.5) | -2.4(-24.9 to 26.8) |
| Republic of Uganda | 61(44 to 81) | 89(61 to 124) | 45.9(12 to 92.3) | 3.9(2.8 to 5.1) | 3.7(2.6 to 5.2) | -2.7(-25.3 to 28.2) |
| Republic of Zambia | 33(22 to 43) | 47(29 to 63) | 44.8(10 to 86.1) | 4.6(3.1 to 6.1) | 4.4(2.7 to 5.9) | -6(-28.6 to 20.9) |
| Republic of Botswana | 6(4 to 9) | 7(5 to 11) | 18.7(-14.4 to 67) | 3.8(2.7 to 5.3) | 3(2.1 to 4.5) | -21.4(-43.3 to 10.6) |
| Kingdom of Lesotho | 8(5 to 12) | 8(5 to 12) | 4.5(-17.4 to 38.4) | 4.3(2.9 to 6.5) | 4.5(3 to 6.8) | 4.5(-17.4 to 38.3) |
| Republic of Namibia | 4(3 to 6) | 6(4 to 8) | 31.1(-3.7 to 68.9) | 2.7(1.9 to 3.5) | 2.6(1.7 to 3.6) | -4.1(-29.6 to 23.6) |
| Republic of South Africa | 231(164 to 272) | 304(200 to 359) | 31.8(12.7 to 51.2) | 4(2.9 to 4.8) | 3.9(2.5 to 4.5) | -4.8(-18.6 to 9.3) |
| Kingdom of Eswatini | 3(2 to 5) | 4(2 to 6) | 12.7(-15.4 to 60.5) | 4.7(2.7 to 7.3) | 4.3(2.7 to 6.4) | -8.1(-31.1 to 30.9) |
| Republic of Zimbabwe | 51(38 to 69) | 61(43 to 85) | 19.9(-6.7 to 53.6) | 5.8(4.3 to 7.8) | 5.5(3.9 to 7.7) | -4.6(-25.8 to 22.2) |
| Republic of Benin | 1(1 to 1) | 2(1 to 2) | 66.8(19 to 118) | 0.2(0.1 to 0.2) | 0.2(0.1 to 0.3) | 11.2(-20.7 to 45.2) |
| Burkina Faso | 2(1 to 3) | 4(2 to 5) | 71.2(20.6 to 124.8) | 0.2(0.1 to 0.3) | 0.3(0.1 to 0.3) | 25.2(-11.8 to 64.4) |
| Republic of Cameroon | 3(2 to 4) | 5(3 to 7) | 67.5(15.2 to 132.7) | 0.2(0.1 to 0.3) | 0.2(0.1 to 0.3) | 11.2(-23.6 to 54.5) |
| Republic of Cabo Verde | 1(0 to 1) | 1(1 to 2) | 33.9(-4 to 69.1) | 2.2(0.8 to 3) | 1.9(0.7 to 2.5) | -13.5(-38 to 9.2) |
| Republic of Chad | 1(1 to 2) | 2(1 to 3) | 54(11.6 to 103.3) | 0.2(0.1 to 0.3) | 0.2(0.1 to 0.3) | 11.3(-19.3 to 46.9) |
| Republic of C么te d'Ivoire | 2(2 to 3) | 4(2 to 5) | 74.1(21.5 to 126.1) | 0.2(0.1 to 0.2) | 0.2(0.1 to 0.3) | 18.1(-17.6 to 53.4) |
| Republic of the Gambia | 0(0 to 0) | 0(0 to 0) | 33.6(5.6 to 67.1) | 0(0 to 0) | 0(0 to 0) | -0.1(-21 to 25) |
| Republic of Ghana | 3(2 to 4) | 7(4 to 9) | 89(32.3 to 154.9) | 0.2(0.1 to 0.2) | 0.2(0.1 to 0.3) | 31.6(-7.9 to 77.5) |
| Republic of Guinea | 2(1 to 2) | 2(1 to 3) | 25.9(-5.2 to 63.4) | 0.2(0.1 to 0.3) | 0.2(0.1 to 0.3) | 7.8(-18.8 to 39.8) |
| Republic of Guinea-Bissau | 0(0 to 0) | 0(0 to 0) | 52.8(13.6 to 98.3) | 0.2(0.2 to 0.3) | 0.3(0.2 to 0.4) | 15.6(-14.1 to 50) |
| Republic of Liberia | 0(0 to 1) | 1(0 to 1) | 47.1(8.1 to 89.2) | 0.2(0.1 to 0.2) | 0.2(0.1 to 0.3) | 4.9(-22.9 to 34.9) |
| Republic of Mali | 12(9 to 18) | 17(12 to 25) | 36.9(8.4 to 68.3) | 1.2(0.9 to 1.8) | 1.1(0.8 to 1.8) | -2(-22.4 to 20.5) |
| Islamic Republic of Mauritania | 0(0 to 1) | 1(0 to 1) | 69.4(22.7 to 120.7) | 0.2(0.1 to 0.2) | 0.2(0.1 to 0.3) | 19.3(-13.6 to 55.5) |
| Republic of the Niger | 1(1 to 2) | 2(1 to 4) | 109.7(44.2 to 184.2) | 0.1(0.1 to 0.2) | 0.2(0.1 to 0.3) | 32.9(-8.6 to 80.1) |
| Federal Republic of Nigeria | 20(16 to 28) | 30(21 to 43) | 49.1(9 to 107.1) | 0.2(0.2 to 0.3) | 0.2(0.1 to 0.3) | 3(-24.7 to 43) |
| Democratic Republic of Sao Tome and Principe | 1(1 to 1) | 1(1 to 2) | 25.6(1.5 to 59.3) | 8.2(5.3 to 11) | 7.1(4.6 to 9.3) | -13.5(-30.1 to 9.7) |
| Republic of Senegal | 2(1 to 2) | 3(2 to 4) | 69.8(28.4 to 117.3) | 0.2(0.1 to 0.3) | 0.2(0.1 to 0.3) | 22.8(-7.1 to 57.2) |
| Republic of Sierra Leone | 1(1 to 1) | 1(1 to 2) | 59.6(14.4 to 108.6) | 0.2(0.1 to 0.3) | 0.2(0.1 to 0.3) | 9.7(-21.4 to 43.3) |
| Togolese Republic | 1(0 to 1) | 1(1 to 2) | 97.5(38.9 to 170) | 0.2(0.1 to 0.2) | 0.2(0.1 to 0.3) | 24.9(-12.2 to 70.8) |
| American Samoa | 0(0 to 0) | 0(0 to 0) | -6.3(-30.1 to 31.2) | 3.2(2.3 to 4) | 2.2(1.7 to 3.4) | -30.2(-47.9 to -2.2) |
| Bermuda | 1(1 to 1) | 1(1 to 1) | 11.6(-5.3 to 35.7) | 4.1(3.7 to 4.6) | 3.4(2.8 to 4.2) | -17.5(-29.9 to 0.3) |
| Cook Islands | 0(0 to 0) | 0(0 to 0) | 26.6(-9.3 to 70) | 2.6(1.9 to 3.2) | 2.4(1.6 to 3.6) | -7.7(-33.9 to 24) |
| Greenland | 1(1 to 1) | 1(1 to 1) | 15(-12.5 to 48.3) | 8.9(6.5 to 11.8) | 6.9(5 to 9.8) | -22.3(-40.9 to 0.2) |
| Guam | 0(0 to 1) | 0(0 to 1) | 6.1(-20.7 to 40.7) | 1.7(1.2 to 2) | 1.2(0.9 to 1.7) | -27.1(-45.5 to -3.3) |
| Principality of Monaco | 1(0 to 1) | 1(0 to 1) | 7.2(-13.4 to 34) | 4.2(3 to 5.4) | 3.9(2.9 to 5.3) | -6.6(-24.6 to 16.7) |
| Republic of Nauru | 0(0 to 0) | 0(0 to 0) | 5(-16.8 to 34.6) | 4.2(2.5 to 6) | 3.9(2.3 to 5.4) | -9.2(-28 to 16.5) |
| Republic of Niue | 0(0 to 0) | 0(0 to 0) | 0.1(-17.3 to 19.3) | 3.5(2.4 to 4.3) | 3.1(2.2 to 3.9) | -10.8(-26.3 to 6.3) |
| Northern Mariana Islands | 0(0 to 0) | 0(0 to 0) | 14.7(-14.1 to 77.3) | 2.3(1.7 to 3) | 1.6(1.3 to 2.9) | -28.8(-46.7 to 10) |
| Republic of Palau | 0(0 to 0) | 0(0 to 0) | 37(3.6 to 81.4) | 2.1(1.4 to 2.8) | 1.9(1.2 to 2.7) | -10.7(-32.5 to 18.2) |
| Puerto Rico | 42(39 to 45) | 44(36 to 52) | 4.3(-15.1 to 23.3) | 4.2(3.9 to 4.5) | 3.8(3.1 to 4.4) | -10.9(-27.4 to 5.3) |
| Saint Kitts and Nevis | 1(0 to 1) | 1(0 to 1) | 13.1(-6.8 to 31.9) | 6.6(6.1 to 7.1) | 4.4(3.7 to 5.2) | -33.3(-45 to -22.2) |
| Republic of San Marino | 1(1 to 2) | 1(1 to 1) | -24.9(-47.4 to 4.4) | 14.6(10.6 to 20) | 8.6(5.5 to 12.6) | -41.1(-58.7 to -18.1) |
| Tokelau | 0(0 to 0) | 0(0 to 0) | -9.2(-27.1 to 10.2) | 3.5(2.4 to 4.6) | 3(2.1 to 4) | -15.9(-32.4 to 2.1) |
| Tuvalu | 0(0 to 0) | 0(0 to 0) | 12.8(-6.7 to 35) | 3.1(2.1 to 4) | 2.8(2 to 3.7) | -8.9(-24.6 to 9) |
| United States Virgin Islands | 1(1 to 2) | 2(1 to 2) | 8.6(-16 to 44) | 4.8(3.7 to 6.6) | 4.7(3.3 to 6.6) | -2.2(-24.4 to 29.6) |
| Republic of South Sudan | 16(10 to 23) | 22(14 to 33) | 38.1(5.3 to 80.6) | 3.5(2.2 to 5) | 3.6(2.2 to 5.3) | 3.5(-21.1 to 35.3) |
| Republic of Sudan | 97(68 to 147) | 130(92 to 191) | 33.9(4.1 to 70.9) | 4.3(3 to 6.6) | 4.2(2.9 to 6.1) | -3.6(-25.1 to 23) |

* GBTC represents gallbladder and biliary tract cancer, ASR represents age-standardized rate.

**Table 2-S3. The DALYs of GBTC between 2010 and 2021 at national level, both sexes in aged 55 years and older**

|  | | | | | | |
| --- | --- | --- | --- | --- | --- | --- |
| region | Case in 2010 | Case in 2021 | Change in number (95% UI) | ASR in 2010 | ASR in 2021 | change in ASR per 100 000 population (95% UI) |
| People's Republic of China | 510774(368251 to 600091) | 683908(482234 to 886803) | 33.9(7.5 to 65.2) | 197.8(142.6 to 232.4) | 180.5(127.3 to 234) | -8.8(-26.8 to 12.6) |
| Democratic People's Republic of Korea | 7627(5135 to 12980) | 8352(5539 to 15077) | 9.5(-12.3 to 34.2) | 176.9(119.1 to 301) | 148.2(98.3 to 267.5) | -16.2(-32.9 to 2.7) |
| Taiwan (Province of China) | 13355(12369 to 14135) | 16237(14611 to 17854) | 21.6(9.9 to 33.1) | 261.6(242.2 to 276.8) | 215.6(194 to 237.1) | -17.6(-25.5 to -9.7) |
| Kingdom of Cambodia | 1374(939 to 2832) | 2411(1530 to 5086) | 75.4(23.5 to 149.5) | 100.8(68.8 to 207.7) | 110.9(70.4 to 234) | 10.1(-22.5 to 56.5) |
| Republic of Indonesia | 20563(14133 to 44197) | 29916(19715 to 64324) | 45.5(13.7 to 76.8) | 76.3(52.4 to 163.9) | 71.4(47.1 to 153.6) | -6.3(-26.8 to 13.8) |
| Lao People's Democratic Republic | 481(299 to 1020) | 687(433 to 1512) | 42.9(5 to 86.7) | 92.3(57.5 to 195.8) | 88.9(56 to 195.5) | -3.7(-29.3 to 25.7) |
| Malaysia | 3548(2366 to 4400) | 5657(3588 to 6881) | 59.5(34.6 to 93.6) | 109.6(73.1 to 135.9) | 115.4(73.2 to 140.3) | 5.3(-11.2 to 27.8) |
| Republic of Maldives | 21(14 to 25) | 28(20 to 36) | 30.6(-0.4 to 67.9) | 71.4(47.5 to 84.7) | 51.6(36.7 to 66.4) | -27.8(-44.9 to -7.1) |
| Republic of the Union of Myanmar | 4483(2778 to 9030) | 6285(4053 to 13240) | 40.2(8.2 to 85.8) | 77.4(47.9 to 155.8) | 74.4(48 to 156.8) | -3.8(-25.7 to 27.6) |
| Republic of the Philippines | 4818(4140 to 8487) | 7415(5811 to 13215) | 53.9(27.7 to 83.1) | 51.8(44.5 to 91.3) | 53.2(41.7 to 94.8) | 2.6(-14.9 to 22) |
| Democratic Socialist Republic of Sri Lanka | 2920(2328 to 5392) | 3722(2235 to 6750) | 27.5(-19.5 to 75.2) | 86.6(69 to 159.9) | 77.3(46.4 to 140.1) | -10.8(-43.6 to 22.7) |
| Kingdom of Thailand | 79999(44728 to 98483) | 130620(63063 to 179217) | 63.3(14.8 to 123.7) | 654.5(365.9 to 805.8) | 672(324.4 to 922) | 2.7(-27.8 to 40.6) |
| Democratic Republic of Timor-Leste | 74(50 to 159) | 104(66 to 223) | 39.9(9.6 to 74.6) | 66.7(45 to 143.3) | 73(46.5 to 157.1) | 9.4(-14.3 to 36.5) |
| Socialist Republic of Viet Nam | 13143(10277 to 18141) | 21458(14633 to 32198) | 63.3(19.4 to 116.2) | 122.6(95.9 to 169.2) | 122.8(83.8 to 184.3) | 0.2(-26.7 to 32.7) |
| Republic of Fiji | 120(75 to 143) | 155(90 to 212) | 28.4(-5.5 to 69.3) | 118.9(73.7 to 141.5) | 113(66.1 to 155.1) | -5(-30.1 to 25.3) |
| Republic of Kiribati | 6(3 to 7) | 7(4 to 10) | 36(8.5 to 69.2) | 64.3(32.3 to 83.5) | 59.5(30.5 to 80.9) | -7.5(-26.2 to 15.1) |
| Republic of the Marshall Islands | 3(2 to 4) | 4(2 to 5) | 34.9(5.6 to 72.8) | 72.1(46.1 to 100.5) | 66.2(42.5 to 93) | -8.2(-28.1 to 17.7) |
| Federated States of Micronesia | 7(5 to 9) | 9(6 to 12) | 28.8(-0.6 to 64.3) | 72.8(47.7 to 98.3) | 67.4(44.8 to 94.1) | -7.3(-28.5 to 18.2) |
| Independent State of Papua New Guinea | 256(159 to 367) | 394(262 to 567) | 54.3(17.1 to 101) | 50.8(31.6 to 72.8) | 48(31.9 to 69.1) | -5.5(-28.2 to 23.2) |
| Independent State of Samoa | 13(9 to 18) | 16(11 to 21) | 18.8(-5.6 to 46.3) | 69.2(46 to 92.2) | 64.6(44.2 to 86) | -6.6(-25.8 to 15) |
| Solomon Islands | 24(13 to 33) | 34(21 to 48) | 43.1(10.2 to 82.4) | 60.1(34.4 to 83.8) | 62.2(39.4 to 89.2) | 3.5(-20.3 to 31.9) |
| Kingdom of Tonga | 7(4 to 9) | 7(5 to 10) | 3.9(-17.4 to 28.3) | 55.6(38.1 to 77.2) | 50.8(35 to 71.9) | -8.6(-27.4 to 12.8) |
| Republic of Vanuatu | 11(7 to 15) | 16(11 to 22) | 45.5(18.6 to 78.8) | 56.5(36.8 to 78.1) | 55.9(38.6 to 76.5) | -1.1(-19.4 to 21.5) |
| Republic of Armenia | 506(431 to 580) | 1377(1151 to 1617) | 172.1(117.1 to 239.6) | 81.5(69.4 to 93.4) | 175(146.4 to 205.5) | 114.8(71.3 to 168) |
| Republic of Azerbaijan | 996(640 to 1459) | 1370(802 to 2059) | 37.5(-14.2 to 113.3) | 90.9(58.4 to 133.1) | 71.5(41.9 to 107.5) | -21.3(-50.9 to 22) |
| Georgia | 1010(842 to 1206) | 1877(1522 to 2272) | 85.9(46.7 to 135.4) | 100.4(83.7 to 119.9) | 178.8(145 to 216.4) | 78(40.5 to 125.5) |
| Republic of Kazakhstan | 3358(3052 to 3692) | 3606(2971 to 4293) | 7.4(-13.4 to 29.1) | 145.7(132.4 to 160.2) | 113.6(93.6 to 135.3) | -22(-37.1 to -6.2) |
| Kyrgyz Republic | 472(395 to 546) | 956(778 to 1165) | 102.4(59.2 to 157.7) | 87.1(72.9 to 100.6) | 113.1(91.9 to 137.7) | 29.8(2.1 to 65.3) |
| Mongolia | 754(539 to 1115) | 1153(829 to 1735) | 53(2.6 to 115.2) | 341.7(244.3 to 505.8) | 292(210.1 to 439.5) | -14.5(-42.7 to 20.2) |
| Republic of Tajikistan | 90(61 to 124) | 125(86 to 173) | 38.7(-5.1 to 104) | 16.6(11.3 to 22.9) | 12.2(8.3 to 16.8) | -27(-50 to 7.4) |
| Turkmenistan | 272(236 to 310) | 618(475 to 814) | 127.5(73.7 to 206.6) | 61.3(53.3 to 70) | 87.3(67.1 to 115) | 42.4(8.7 to 91.9) |
| Republic of Uzbekistan | 679(545 to 847) | 1959(1518 to 2522) | 188.4(112.4 to 296.3) | 24.8(19.9 to 30.9) | 42.1(32.7 to 54.2) | 69.9(25.1 to 133.5) |
| Republic of Albania | 749(483 to 1173) | 999(667 to 1477) | 33.3(-13.1 to 98) | 125.3(80.7 to 196.3) | 127(84.8 to 187.8) | 1.4(-33.9 to 50.5) |
| Bosnia and Herzegovina | 3739(3017 to 6191) | 3643(2643 to 6210) | -2.6(-27.2 to 25.7) | 375.4(303 to 621.6) | 332.6(241.3 to 567) | -11.4(-33.8 to 14.3) |
| Republic of Bulgaria | 4135(3814 to 4427) | 3565(3001 to 4232) | -13.8(-28.2 to 3.1) | 172.6(159.2 to 184.8) | 150(126.2 to 178) | -13.1(-27.6 to 3.9) |
| Republic of Croatia | 4497(4102 to 4913) | 4419(3779 to 5041) | -1.7(-16.7 to 14.2) | 334.4(305.1 to 365.4) | 296.3(253.4 to 338) | -11.4(-24.9 to 3) |
| Czech Republic | 15305(13337 to 16822) | 14501(12272 to 17016) | -5.3(-20.2 to 12.5) | 486.7(424.1 to 535) | 415.1(351.3 to 487.1) | -14.7(-28.2 to 1.2) |
| Hungary | 12548(11702 to 13411) | 10646(9252 to 11991) | -15.2(-25.8 to -3.6) | 411.8(384 to 440.1) | 334.8(290.9 to 377.1) | -18.7(-28.9 to -7.6) |
| North Macedonia | 1350(1016 to 1649) | 1449(1056 to 1876) | 7.3(-16.9 to 35.1) | 279.2(210.2 to 341) | 244.3(178.1 to 316.4) | -12.5(-32.3 to 10.1) |
| Montenegro | 261(191 to 345) | 275(197 to 372) | 5.7(-21 to 39.8) | 168.6(123.5 to 222.9) | 157.9(113 to 213.1) | -6.3(-29.9 to 23.9) |
| Republic of Poland | 40661(38484 to 42226) | 40416(36754 to 43730) | -0.6(-8.3 to 7.4) | 395(373.9 to 410.2) | 333.6(303.4 to 360.9) | -15.6(-22.1 to -8.7) |
| Romania | 10561(9799 to 11254) | 10725(9318 to 12235) | 1.6(-13 to 15.9) | 179.9(166.9 to 191.7) | 178.6(155.2 to 203.7) | -0.7(-15 to 13.3) |
| Republic of Serbia | 6939(5149 to 8789) | 6961(4861 to 9279) | 0.3(-24.5 to 30) | 267.4(198.4 to 338.7) | 248.1(173.3 to 330.8) | -7.2(-30.2 to 20.2) |
| Slovak Republic | 5734(4462 to 7280) | 6735(4884 to 9156) | 17.4(-11.5 to 49) | 423.6(329.6 to 537.8) | 410.6(297.8 to 558.2) | -3.1(-27 to 23) |
| Republic of Slovenia | 1975(1795 to 2154) | 1912(1621 to 2219) | -3.2(-17.7 to 12.6) | 328.2(298.2 to 357.9) | 261.2(221.4 to 303) | -20.4(-32.4 to -7.5) |
| Republic of Belarus | 3971(3604 to 4322) | 4191(3366 to 5114) | 5.5(-13.5 to 31.6) | 161.7(146.8 to 176.1) | 145.7(117 to 177.8) | -9.9(-26.2 to 12.3) |
| Republic of Estonia | 710(644 to 767) | 831(714 to 944) | 17(1.8 to 33.9) | 179.9(163 to 194.4) | 190(163.3 to 215.9) | 5.6(-8.1 to 20.9) |
| Republic of Latvia | 892(826 to 963) | 918(794 to 1057) | 2.9(-10.1 to 18.9) | 140.7(130.2 to 152) | 139.5(120.6 to 160.7) | -0.9(-13.4 to 14.6) |
| Republic of Lithuania | 1534(1388 to 1664) | 1959(1699 to 2231) | 27.7(11 to 43.9) | 173.3(156.9 to 188) | 202.4(175.5 to 230.6) | 16.8(1.5 to 31.6) |
| Republic of Moldova | 975(918 to 1032) | 1035(934 to 1151) | 6.2(-5.8 to 20.1) | 110.1(103.7 to 116.5) | 98(88.5 to 109) | -11(-21 to 0.7) |
| Russian Federation | 54302(52354 to 55781) | 64124(58827 to 69359) | 18.1(8.7 to 27.7) | 151(145.6 to 155.1) | 150.9(138.4 to 163.2) | -0.1(-8 to 8.1) |
| Ukraine | 17718(16655 to 18778) | 15651(11636 to 20297) | -11.7(-33.5 to 16.5) | 142.8(134.2 to 151.3) | 115.3(85.7 to 149.5) | -19.3(-39.3 to 6.5) |
| Brunei Darussalam | 92(70 to 114) | 134(106 to 176) | 46.6(22 to 79.8) | 266.9(204.2 to 333.3) | 224.3(177.6 to 293.7) | -15.9(-30.1 to 3.1) |
| Japan | 301845(264871 to 321865) | 303178(256784 to 329623) | 0.4(-4.1 to 3.4) | 620.4(544.4 to 661.5) | 580.8(491.9 to 631.5) | -6.4(-10.6 to -3.6) |
| Republic of Korea | 67512(47311 to 81994) | 92635(57631 to 116711) | 37.2(13 to 63.1) | 645.8(452.6 to 784.3) | 553.9(344.6 to 697.9) | -14.2(-29.4 to 2) |
| Republic of Singapore | 1071(985 to 1154) | 1460(1314 to 1580) | 36.2(23.9 to 49.1) | 123(113.1 to 132.5) | 96.1(86.5 to 104) | -21.9(-28.9 to -14.5) |
| Australia | 7040(6359 to 7474) | 7909(7017 to 8525) | 12.3(5.6 to 19.1) | 129.8(117.2 to 137.8) | 106.8(94.7 to 115.1) | -17.7(-22.7 to -12.7) |
| New Zealand | 1355(1231 to 1452) | 2416(2158 to 2623) | 78.3(65.2 to 91.3) | 129.1(117.4 to 138.3) | 169.4(151.3 to 183.9) | 31.2(21.5 to 40.7) |
| Principality of Andorra | 48(35 to 66) | 47(32 to 65) | -3.4(-30.5 to 29.9) | 242.9(176.5 to 329.4) | 176.6(119.4 to 244) | -27.3(-47.6 to -2.1) |
| Republic of Austria | 5782(5284 to 6145) | 5340(4781 to 5785) | -7.6(-14 to -1) | 236.4(216 to 251.2) | 180.7(161.8 to 195.8) | -23.6(-28.8 to -18.1) |
| Kingdom of Belgium | 4245(3830 to 4554) | 3529(3119 to 3863) | -16.9(-23.1 to -10.9) | 132.7(119.7 to 142.4) | 93.3(82.5 to 102.2) | -29.7(-34.9 to -24.6) |
| Republic of Cyprus | 433(336 to 546) | 472(351 to 620) | 9(-13.2 to 37.2) | 177.6(137.6 to 223.8) | 135.1(100.5 to 177.4) | -23.9(-39.4 to -4.2) |
| Kingdom of Denmark | 2241(2067 to 2376) | 2225(2026 to 2412) | -0.7(-7.8 to 7.4) | 137.4(126.7 to 145.7) | 115.6(105.2 to 125.3) | -15.9(-21.9 to -9) |
| Republic of Finland | 3465(3145 to 3698) | 4159(3666 to 4539) | 20(9.1 to 30.6) | 199.9(181.4 to 213.3) | 206.7(182.1 to 225.5) | 3.4(-6.1 to 12.5) |
| French Republic | 26344(23491 to 28128) | 23419(20512 to 26068) | -11.1(-18.7 to -3.4) | 142(126.6 to 151.6) | 105.9(92.8 to 117.9) | -25.4(-31.8 to -18.9) |
| Federal Republic of Germany | 64625(58998 to 68409) | 70278(62638 to 75810) | 8.7(2.3 to 15.4) | 240.8(219.8 to 254.9) | 223(198.8 to 240.6) | -7.4(-12.9 to -1.7) |
| Hellenic Republic | 8717(8047 to 9285) | 5615(5123 to 5998) | -35.6(-39.8 to -30.9) | 257.8(238 to 274.6) | 152.8(139.4 to 163.2) | -40.7(-44.7 to -36.4) |
| Republic of Iceland | 87(79 to 95) | 97(84 to 108) | 11.5(0.5 to 22.5) | 121.1(110.7 to 131.9) | 99.5(86.4 to 110.9) | -17.9(-25.9 to -9.7) |
| Ireland | 1042(945 to 1127) | 1032(905 to 1136) | -0.9(-9.2 to 7.7) | 106.5(96.7 to 115.2) | 78.7(69 to 86.5) | -26.2(-32.3 to -19.8) |
| State of Israel | 1765(1592 to 1896) | 1745(1561 to 1919) | -1.1(-9.7 to 7.7) | 118.9(107.3 to 127.7) | 87.3(78.1 to 96) | -26.6(-32.9 to -20) |
| Republic of Italy | 64905(58323 to 68944) | 63411(56544 to 68431) | -2.3(-5.8 to 1.3) | 331.2(297.6 to 351.8) | 279.1(248.9 to 301.2) | -15.7(-18.7 to -12.7) |
| Grand Duchy of Luxembourg | 163(151 to 175) | 182(162 to 202) | 11.8(1 to 24.9) | 130.3(120.6 to 139.7) | 102.5(91.1 to 113.5) | -21.3(-28.9 to -12.1) |
| Republic of Malta | 122(112 to 132) | 130(114 to 145) | 6.8(-5.2 to 18.7) | 96.3(88.3 to 104.5) | 82.4(72.2 to 92.2) | -14.4(-24.1 to -4.9) |
| Kingdom of the Netherlands | 7767(7062 to 8249) | 8894(7937 to 9698) | 14.5(5 to 23.5) | 165.1(150.1 to 175.4) | 152(135.7 to 165.8) | -7.9(-15.5 to -0.7) |
| Kingdom of Norway | 1456(1329 to 1528) | 1680(1505 to 1790) | 15.4(10.5 to 20.2) | 110.8(101.1 to 116.2) | 103.6(92.8 to 110.4) | -6.5(-10.5 to -2.6) |
| Portuguese Republic | 5737(5195 to 6135) | 6060(5386 to 6593) | 5.6(-2.1 to 13.9) | 173.7(157.3 to 185.7) | 155.8(138.4 to 169.5) | -10.3(-16.8 to -3.3) |
| Kingdom of Spain | 22305(19929 to 24042) | 23532(20368 to 25846) | 5.5(-2 to 13.4) | 171.4(153.2 to 184.8) | 152.1(131.6 to 167) | -11.3(-17.6 to -4.7) |
| Kingdom of Sweden | 8045(7305 to 8554) | 9519(8293 to 10624) | 18.3(6.6 to 31.7) | 273.7(248.5 to 291) | 282.8(246.3 to 315.6) | 3.3(-6.9 to 15) |
| Swiss Confederation | 2729(2448 to 2979) | 3370(2909 to 3827) | 23.5(13.2 to 34.7) | 118.7(106.5 to 129.6) | 115(99.3 to 130.6) | -3.1(-11.2 to 5.7) |
| United Kingdom of Great Britain and Northern Ireland | 17042(15875 to 17749) | 23354(21333 to 24537) | 37(33 to 40.5) | 96.3(89.7 to 100.3) | 111(101.4 to 116.7) | 15.3(11.9 to 18.2) |
| Argentine Republic | 34121(32132 to 36142) | 31917(29504 to 34075) | -6.5(-12.5 to 1) | 442.8(417 to 469) | 342(316.1 to 365.1) | -22.8(-27.7 to -16.6) |
| Republic of Chile | 35995(33677 to 37886) | 36062(32960 to 39077) | 0.2(-7.1 to 8.6) | 1184.8(1108.5 to 1247) | 805.4(736.1 to 872.7) | -32(-37 to -26.3) |
| Eastern Republic of Uruguay | 3788(3506 to 4019) | 3873(3518 to 4225) | 2.2(-5.8 to 11.7) | 481.2(445.4 to 510.5) | 428.3(389 to 467.2) | -11(-18 to -2.8) |
| Canada | 12010(10941 to 12739) | 11947(10692 to 12904) | -0.5(-7.3 to 7.3) | 132.3(120.5 to 140.3) | 97.4(87.1 to 105.2) | -26.4(-31.4 to -20.6) |
| United States of America | 74109(68426 to 77514) | 94063(87053 to 98678) | 26.9(24.1 to 29.6) | 97.2(89.7 to 101.6) | 93.8(86.8 to 98.4) | -3.4(-5.6 to -1.4) |
| Antigua and Barbuda | 18(16 to 19) | 21(20 to 23) | 20.8(10.4 to 32) | 140.9(129.8 to 151.3) | 113.8(107.1 to 121.1) | -19.2(-26.1 to -11.7) |
| Commonwealth of the Bahamas | 80(74 to 87) | 106(87 to 129) | 32.5(7.5 to 65.6) | 170.6(157.5 to 185.8) | 147.3(120.3 to 179.2) | -13.6(-29.9 to 7.9) |
| Barbados | 96(90 to 101) | 124(95 to 154) | 29.8(-0.2 to 60.8) | 147.3(137.9 to 155.2) | 136.3(104.7 to 169.1) | -7.5(-28.8 to 14.6) |
| Belize | 41(38 to 43) | 54(47 to 61) | 31.8(15.3 to 50.6) | 142.1(133.3 to 151) | 107.1(93.7 to 121.8) | -24.6(-34.1 to -13.9) |
| Republic of Cuba | 3267(3063 to 3469) | 3302(2856 to 3741) | 1.1(-13.6 to 16) | 124.8(117 to 132.5) | 96.6(83.6 to 109.5) | -22.6(-33.8 to -11.1) |
| Commonwealth of Dominica | 22(16 to 28) | 26(19 to 33) | 17.5(-4.2 to 46.8) | 192.4(142.6 to 243.1) | 172.2(126.8 to 218.3) | -10.5(-27.1 to 11.8) |
| Dominican Republic | 995(756 to 1437) | 1446(1078 to 2089) | 45.3(10 to 89.4) | 82.8(63 to 119.6) | 86.5(64.5 to 125) | 4.5(-20.9 to 36.2) |
| Grenada | 27(25 to 30) | 29(25 to 34) | 8.5(-6.7 to 25.2) | 167.5(153.5 to 182.3) | 146.5(125.2 to 167.8) | -12.5(-24.8 to 0.9) |
| Republic of Guyana | 106(97 to 117) | 131(103 to 167) | 23.3(-4.7 to 55.9) | 129.1(117.2 to 141.9) | 116.5(91.2 to 148.2) | -9.7(-30.2 to 14.2) |
| Republic of Haiti | 1545(888 to 2312) | 1946(1197 to 2978) | 25.9(-1.3 to 63.4) | 180(103.4 to 269.4) | 164.2(101 to 251.3) | -8.8(-28.5 to 18.3) |
| Jamaica | 480(440 to 518) | 597(457 to 759) | 24.5(-6.1 to 59.4) | 116.9(107.1 to 126.1) | 112.9(86.3 to 143.6) | -3.4(-27.1 to 23.7) |
| Saint Lucia | 23(21 to 24) | 35(28 to 42) | 53.3(23.7 to 84.7) | 84.9(79.4 to 90.5) | 82.1(66.1 to 98.9) | -3.4(-22 to 16.4) |
| Saint Vincent and the Grenadines | 18(17 to 20) | 22(20 to 25) | 22.3(5.4 to 41.7) | 105.3(97.6 to 112.8) | 87.7(77.6 to 100.2) | -16.7(-28.2 to -3.5) |
| Republic of Suriname | 83(65 to 110) | 108(77 to 149) | 31.1(-4 to 73.6) | 113.9(90 to 152) | 97.1(68.5 to 133.1) | -14.7(-37.5 to 12.9) |
| Republic of Trinidad and Tobago | 259(243 to 274) | 366(285 to 466) | 41.2(8.3 to 80.8) | 106.3(99.7 to 112.5) | 105(81.9 to 133.7) | -1.2(-24.2 to 26.5) |
| Plurinational State of Bolivia | 7471(4757 to 10379) | 9561(6093 to 13625) | 28(1.5 to 65) | 672.9(428.4 to 934.8) | 618.7(394.2 to 881.6) | -8.1(-27.1 to 18.5) |
| Republic of Ecuador | 7455(6789 to 8187) | 8644(6799 to 10818) | 15.9(-10.2 to 48.8) | 398.8(363.2 to 438) | 312.3(245.7 to 390.9) | -21.7(-39.4 to 0.5) |
| Republic of Peru | 16246(11638 to 20042) | 24035(16556 to 34169) | 47.9(5.7 to 105.2) | 424.3(303.9 to 523.4) | 429.7(296 to 610.9) | 1.3(-27.6 to 40.4) |
| Republic of Colombia | 17229(15993 to 18553) | 24097(19945 to 28319) | 39.9(15.3 to 65.4) | 270.8(251.4 to 291.6) | 252(208.6 to 296.1) | -7(-23.3 to 10) |
| Republic of Costa Rica | 1286(1173 to 1383) | 1872(1634 to 2130) | 45.5(27.2 to 64.7) | 208.5(190.2 to 224.2) | 195.5(170.7 to 222.4) | -6.2(-18 to 6.1) |
| Republic of El Salvador | 2006(1661 to 2870) | 2744(2104 to 3813) | 36.8(8.7 to 70.3) | 250.8(207.7 to 358.8) | 268.3(205.7 to 372.8) | 7(-15 to 33.2) |
| Republic of Guatemala | 2838(2707 to 2968) | 3098(2650 to 3589) | 9.2(-6.7 to 27.2) | 230.1(219.5 to 240.7) | 169.2(144.7 to 196) | -26.5(-37.2 to -14.3) |
| Republic of Honduras | 2442(1671 to 3333) | 3836(2611 to 5181) | 57.1(18 to 106) | 344(235.5 to 469.6) | 364.7(248.3 to 492.7) | 6(-20.4 to 39) |
| United Mexican States | 37122(35802 to 37952) | 49347(43315 to 55336) | 32.9(17.3 to 48.4) | 262(252.7 to 267.8) | 229(201 to 256.8) | -12.6(-22.9 to -2.4) |
| Republic of Nicaragua | 1439(1162 to 1986) | 1861(1440 to 2934) | 29.4(2.1 to 63.1) | 269.3(217.5 to 371.8) | 228.6(176.9 to 360.5) | -15.1(-33 to 7) |
| Republic of Panama | 688(641 to 729) | 931(740 to 1116) | 35.2(5.1 to 61.7) | 138.4(129 to 146.7) | 125.1(99.5 to 150.1) | -9.6(-29.7 to 8.1) |
| Bolivarian Republic of Venezuela | 5883(5558 to 6193) | 8747(6447 to 11297) | 48.7(8.8 to 91.4) | 168.9(159.5 to 177.8) | 167.2(123.2 to 215.9) | -1(-27.6 to 27.4) |
| Federative Republic of Brazil | 71883(67891 to 74767) | 103356(95954 to 108212) | 43.8(38.2 to 49.2) | 245.8(232.1 to 255.7) | 238.7(221.6 to 249.9) | -2.9(-6.6 to 0.8) |
| Republic of Paraguay | 1377(989 to 1757) | 1947(1351 to 2688) | 41.5(5.7 to 85.6) | 200.6(144.1 to 256) | 196.7(136.5 to 271.4) | -1.9(-26.7 to 28.7) |
| People's Democratic Republic of Algeria | 11944(8714 to 15867) | 17305(12457 to 22472) | 44.9(15.4 to 79.8) | 307.4(224.2 to 408.3) | 284.9(205.1 to 370) | -7.3(-26.1 to 15) |
| Kingdom of Bahrain | 61(41 to 77) | 110(71 to 155) | 78.8(31.4 to 145.8) | 80.5(53.7 to 100.7) | 67.5(43.5 to 95.2) | -16.2(-38.4 to 15.3) |
| Arab Republic of Egypt | 11010(7688 to 13094) | 14874(10291 to 18835) | 35.1(7.3 to 69.8) | 146(101.9 to 173.6) | 134.4(93 to 170.2) | -7.9(-26.9 to 15.7) |
| Islamic Republic of Iran | 7411(4104 to 8609) | 10131(5627 to 11738) | 36.7(25.9 to 48) | 87.7(48.5 to 101.8) | 78(43.3 to 90.4) | -11(-18.1 to -3.7) |
| Republic of Iraq | 2406(1691 to 3237) | 4028(2755 to 5299) | 67.4(20.5 to 122.4) | 98(68.9 to 131.8) | 103.7(70.9 to 136.4) | 5.8(-23.9 to 40.6) |
| Hashemite Kingdom of Jordan | 804(554 to 995) | 1390(946 to 1957) | 72.8(31.5 to 129.4) | 152.3(105 to 188.4) | 110.5(75.3 to 155.7) | -27.4(-44.8 to -3.6) |
| State of Kuwait | 271(249 to 295) | 417(339 to 498) | 53.6(26.9 to 86.4) | 130.9(119.8 to 142) | 89.5(72.6 to 106.9) | -31.6(-43.5 to -17.1) |
| Lebanese Republic | 1296(812 to 1648) | 1434(886 to 1903) | 10.6(-11.7 to 37.9) | 180.3(112.9 to 229.1) | 146.3(90.4 to 194.2) | -18.8(-35.2 to 1.2) |
| State of Libya | 2059(1527 to 2503) | 2970(2026 to 4029) | 44.3(9.8 to 86) | 397.7(295 to 483.5) | 355.2(242.3 to 481.7) | -10.7(-32 to 15.1) |
| Kingdom of Morocco | 2895(1772 to 3938) | 4294(2477 to 5515) | 48.3(12.6 to 91.1) | 72.5(44.4 to 98.7) | 71.5(41.2 to 91.8) | -1.5(-25.2 to 26.9) |
| Palestine | 421(269 to 503) | 531(338 to 669) | 26(-0.1 to 60.2) | 162.4(103.9 to 194) | 123.3(78.7 to 155.5) | -24.1(-39.8 to -3.4) |
| Sultanate of Oman | 163(116 to 203) | 202(142 to 279) | 23.7(-3.1 to 59) | 98.3(70 to 122.2) | 64.7(45.5 to 89.2) | -34.2(-48.5 to -15.4) |
| State of Qatar | 88(64 to 114) | 141(93 to 224) | 59.9(7.3 to 140.2) | 132.5(96.4 to 170.8) | 92(60.9 to 146.3) | -30.6(-53.5 to 4.2) |
| Kingdom of Saudi Arabia | 3164(2564 to 4417) | 4356(3236 to 6489) | 37.7(8.5 to 68.4) | 178.3(144.5 to 249) | 144.7(107.5 to 215.5) | -18.9(-36 to -0.7) |
| Syrian Arab Republic | 141(79 to 181) | 190(107 to 258) | 34.2(-2.3 to 75.9) | 8.9(5 to 11.4) | 8(4.5 to 10.9) | -10.1(-34.5 to 17.8) |
| Republic of Tunisia | 3573(2332 to 4799) | 5014(3325 to 6964) | 40.3(3.8 to 91.9) | 234.4(153 to 314.9) | 214.5(142.3 to 298) | -8.5(-32.3 to 25.2) |
| Republic of Turkey | 18094(12706 to 21875) | 22024(16374 to 29926) | 21.7(-6.3 to 57.4) | 162.9(114.4 to 196.9) | 133.3(99.1 to 181.1) | -18.2(-37 to 5.8) |
| United Arab Emirates | 700(479 to 976) | 1426(1037 to 2029) | 103.6(45.7 to 203) | 369.2(252.3 to 514.4) | 198.6(144.4 to 282.6) | -46.2(-61.5 to -19.9) |
| Republic of Yemen | 1633(1127 to 2496) | 2444(1560 to 3674) | 49.7(13.8 to 94.7) | 106(73.2 to 162.1) | 108.7(69.4 to 163.4) | 2.5(-22.1 to 33.4) |
| Islamic Republic of Afghanistan | 2561(1255 to 4417) | 2519(1194 to 3981) | -1.6(-21.4 to 27.9) | 202(99 to 348.4) | 205(97.2 to 324) | 1.5(-18.9 to 32) |
| People's Republic of Bangladesh | 30067(21204 to 42674) | 46538(31382 to 73296) | 54.8(17.6 to 103.5) | 211.4(149.1 to 300) | 199.2(134.3 to 313.7) | -5.8(-28.4 to 23.9) |
| Kingdom of Bhutan | 169(125 to 248) | 234(159 to 341) | 38.7(12.4 to 74) | 228.4(168.9 to 335.9) | 234.8(159.4 to 342.1) | 2.8(-16.7 to 29) |
| Republic of India | 317291(214150 to 368326) | 507327(345752 to 600888) | 59.9(39.3 to 81.9) | 223.5(150.8 to 259.4) | 252.3(172 to 298.9) | 12.9(-1.6 to 28.5) |
| Federal Democratic Republic of Nepal | 5860(4308 to 8240) | 9327(6807 to 12905) | 59.2(22.2 to 105.3) | 203.5(149.6 to 286.1) | 235.7(172 to 326.1) | 15.8(-11.1 to 49.4) |
| Islamic Republic of Pakistan | 51844(38882 to 67564) | 70001(50602 to 93258) | 35(1.3 to 77.5) | 376.9(282.7 to 491.2) | 353.1(255.2 to 470.4) | -6.3(-29.7 to 23.1) |
| Republic of Angola | 488(339 to 640) | 776(508 to 1072) | 59(17.5 to 112.1) | 41.4(28.7 to 54.3) | 40.1(26.3 to 55.5) | -3.1(-28.4 to 29.3) |
| Central African Republic | 104(64 to 169) | 134(82 to 223) | 28.2(3 to 61.9) | 40.7(24.9 to 65.8) | 37.4(23.1 to 62.2) | -8(-26.1 to 16.1) |
| Republic of the Congo | 127(88 to 177) | 191(123 to 259) | 49.9(19.5 to 96.8) | 45.4(31.2 to 63) | 43.9(28.4 to 59.7) | -3.2(-22.9 to 27) |
| Democratic Republic of the Congo | 1278(823 to 1997) | 2023(1279 to 3113) | 58.4(22.5 to 103.1) | 31.8(20.5 to 49.7) | 33.5(21.2 to 51.5) | 5.3(-18.5 to 35) |
| Republic of Equatorial Guinea | 22(13 to 36) | 32(19 to 48) | 45.4(0.1 to 110.7) | 40.7(24 to 66.8) | 40.1(24.3 to 61.1) | -1.6(-32.2 to 42.7) |
| Gabonese Republic | 59(38 to 78) | 77(49 to 104) | 31.7(3.5 to 68.6) | 46.2(30.3 to 61.5) | 43.2(27.4 to 58.3) | -6.4(-26.5 to 19.8) |
| Republic of Burundi | 426(268 to 579) | 637(366 to 941) | 49.5(16.4 to 91.8) | 84(52.8 to 114.1) | 79.6(45.7 to 117.5) | -5.2(-26.2 to 21.6) |
| Union of the Comoros | 50(33 to 73) | 71(44 to 111) | 42.7(12.6 to 81.2) | 86.5(56.9 to 127.9) | 87.7(54.2 to 137.4) | 1.4(-20 to 28.8) |
| Republic of Djibouti | 50(29 to 78) | 85(49 to 139) | 69.1(27.6 to 132.5) | 83.6(48.3 to 129.7) | 81.6(47.6 to 134.5) | -2.4(-26.3 to 34.2) |
| State of Eritrea | 326(204 to 476) | 463(271 to 689) | 41.9(13.2 to 81.8) | 106.5(66.5 to 155.5) | 105.1(61.4 to 156.4) | -1.3(-21.3 to 26.4) |
| Federal Democratic Republic of Ethiopia | 6994(4748 to 9673) | 9987(6782 to 14326) | 42.8(14.3 to 73.4) | 141.9(96.3 to 196.2) | 145.9(99.1 to 209.3) | 2.8(-17.7 to 24.9) |
| Republic of Kenya | 3010(2240 to 4291) | 4535(3237 to 6496) | 50.7(23.1 to 85.1) | 122(90.8 to 173.9) | 121.2(86.5 to 173.7) | -0.6(-18.8 to 22.1) |
| Republic of Madagascar | 801(516 to 1116) | 1253(766 to 1751) | 56.5(20.4 to 101.8) | 67.8(43.7 to 94.5) | 67.8(41.5 to 94.8) | 0.1(-23 to 29.1) |
| Republic of Malawi | 252(161 to 339) | 335(202 to 489) | 32.7(-0.5 to 73.4) | 29.7(19 to 39.9) | 28.6(17.3 to 41.8) | -3.7(-27.8 to 25.9) |
| Republic of Mauritius | 174(164 to 186) | 275(253 to 293) | 57.7(44.6 to 71.1) | 78.2(73.7 to 83.5) | 81.2(74.7 to 86.7) | 3.9(-4.8 to 12.7) |
| Republic of Mozambique | 1322(776 to 1940) | 1735(959 to 2589) | 31.2(-2.3 to 74.2) | 95.5(56.1 to 140.1) | 97(53.6 to 144.7) | 1.5(-24.4 to 34.8) |
| Republic of Rwanda | 623(416 to 867) | 1041(678 to 1465) | 67.3(28.8 to 118.1) | 95.7(64 to 133.3) | 98.5(64.2 to 138.5) | 2.9(-20.8 to 34.2) |
| Republic of Seychelles | 14(12 to 21) | 18(14 to 26) | 25.3(1.3 to 47.5) | 110.1(90.2 to 159.6) | 86.8(69.2 to 126.1) | -21.1(-36.2 to -7.1) |
| Federal Republic of Somalia | 664(393 to 1124) | 855(488 to 1436) | 28.8(-0.5 to 62.3) | 88.5(52.4 to 149.8) | 90.7(51.7 to 152.3) | 2.5(-20.8 to 29.2) |
| United Republic of Tanzania | 2607(1750 to 3610) | 3598(2376 to 5056) | 38(4 to 84.7) | 89.4(60.1 to 123.9) | 87.3(57.6 to 122.7) | -2.4(-26.5 to 30.6) |
| Republic of Uganda | 1344(962 to 1812) | 1960(1313 to 2769) | 45.8(9.9 to 92.4) | 85(60.9 to 114.6) | 82.6(55.4 to 116.8) | -2.8(-26.8 to 28.2) |
| Republic of Zambia | 732(495 to 972) | 1067(655 to 1444) | 45.7(9.6 to 89.7) | 104.3(70.6 to 138.5) | 98.7(60.7 to 133.7) | -5.3(-28.8 to 23.2) |
| Republic of Botswana | 133(96 to 185) | 161(111 to 245) | 21.1(-15.2 to 70.5) | 82.8(59.5 to 115) | 66.4(45.7 to 101) | -19.8(-43.8 to 12.9) |
| Kingdom of Lesotho | 180(117 to 271) | 187(123 to 282) | 4.1(-18.8 to 38.3) | 99.7(65 to 150.1) | 103.8(67.9 to 156.1) | 4(-18.8 to 38.3) |
| Republic of Namibia | 99(68 to 129) | 130(86 to 186) | 31.6(-5.6 to 72.1) | 59.7(41.3 to 78) | 57.5(37.9 to 82.3) | -3.7(-30.9 to 26) |
| Republic of South Africa | 4946(3567 to 5858) | 6600(4297 to 7790) | 33.4(13.4 to 55) | 86.8(62.6 to 102.8) | 83.7(54.5 to 98.8) | -3.6(-18.1 to 12) |
| Kingdom of Eswatini | 82(45 to 128) | 90(57 to 136) | 9.3(-19.6 to 60.4) | 111.5(61.3 to 173.4) | 99.3(62.5 to 149.9) | -10.9(-34.4 to 30.8) |
| Republic of Zimbabwe | 1172(852 to 1585) | 1426(984 to 1993) | 21.7(-6.2 to 57.5) | 133.1(96.7 to 180) | 128.8(88.9 to 180) | -3.2(-25.4 to 25.3) |
| Republic of Benin | 20(14 to 27) | 33(20 to 45) | 67.9(20.2 to 121.5) | 3.6(2.6 to 4.8) | 4(2.4 to 5.4) | 11.9(-19.9 to 47.6) |
| Burkina Faso | 43(31 to 57) | 73(42 to 98) | 71.2(21.9 to 124.1) | 3.9(2.8 to 5.2) | 4.9(2.8 to 6.5) | 25.2(-10.9 to 63.9) |
| Republic of Cameroon | 58(41 to 82) | 97(57 to 138) | 66.7(14.9 to 131.8) | 4.3(3 to 6) | 4.8(2.8 to 6.8) | 10.6(-23.8 to 53.8) |
| Republic of Cabo Verde | 19(7 to 27) | 28(10 to 38) | 43(2 to 84.9) | 39.4(14.3 to 55.2) | 36.4(13.1 to 49.6) | -7.7(-34.1 to 19.4) |
| Republic of Chad | 26(19 to 37) | 40(27 to 54) | 57.1(13.5 to 110.1) | 3.8(2.9 to 5.5) | 4.3(2.9 to 5.8) | 13.5(-18 to 51.8) |
| Republic of C么te d'Ivoire | 44(34 to 58) | 78(45 to 105) | 74.7(24.4 to 128.6) | 3.6(2.8 to 4.8) | 4.3(2.5 to 5.9) | 18.5(-15.6 to 55.1) |
| Republic of the Gambia | 0(0 to 0) | 0(0 to 0) | 35.5(6.2 to 72.3) | 0(0 to 0) | 0(0 to 0) | 1.4(-20.6 to 28.9) |
| Republic of Ghana | 70(52 to 89) | 130(82 to 169) | 86.9(32.2 to 151.8) | 3.7(2.7 to 4.7) | 4.8(3 to 6.2) | 30.2(-8 to 75.4) |
| Republic of Guinea | 31(22 to 44) | 40(25 to 56) | 30.7(-1.4 to 71.2) | 3.9(2.8 to 5.6) | 4.4(2.7 to 6.1) | 11.9(-15.6 to 46.5) |
| Republic of Guinea-Bissau | 4(3 to 6) | 6(4 to 9) | 52.8(14 to 102) | 4.8(3.2 to 7.2) | 5.6(3.6 to 7.8) | 15.6(-13.7 to 52.8) |
| Republic of Liberia | 9(6 to 12) | 13(8 to 19) | 52.4(12 to 97.9) | 3.6(2.5 to 4.9) | 4(2.3 to 5.6) | 8.7(-20.1 to 41.1) |
| Republic of Mali | 275(211 to 409) | 375(264 to 572) | 36.6(5.8 to 70.5) | 26.5(20.3 to 39.5) | 25.9(18.2 to 39.5) | -2.2(-24.3 to 22) |
| Islamic Republic of Mauritania | 9(6 to 13) | 15(9 to 22) | 71.5(25.1 to 124.3) | 3.5(2.4 to 5) | 4.2(2.5 to 6.1) | 20.8(-11.9 to 58) |
| Republic of the Niger | 25(17 to 39) | 51(32 to 74) | 107.8(45.3 to 181.8) | 2.8(1.9 to 4.4) | 3.7(2.3 to 5.3) | 31.7(-7.9 to 78.7) |
| Federal Republic of Nigeria | 440(328 to 598) | 681(455 to 955) | 54.7(9.8 to 122.3) | 4.3(3.2 to 5.8) | 4.5(3 to 6.4) | 6.9(-24.2 to 53.5) |
| Democratic Republic of Sao Tome and Principe | 22(14 to 30) | 30(18 to 39) | 32.9(6.7 to 68.9) | 176.3(110.1 to 236.1) | 161.4(100.5 to 213.5) | -8.5(-26.5 to 16.3) |
| Republic of Senegal | 33(24 to 47) | 57(34 to 82) | 70(28.7 to 119.2) | 3.6(2.5 to 5.1) | 4.4(2.7 to 6.3) | 23(-6.9 to 58.6) |
| Republic of Sierra Leone | 16(11 to 22) | 26(15 to 35) | 61.5(16.6 to 113) | 3.8(2.7 to 5.3) | 4.3(2.5 to 5.9) | 11(-19.9 to 46.3) |
| Togolese Republic | 15(10 to 21) | 28(17 to 42) | 93.7(37 to 164) | 3.7(2.6 to 5.3) | 4.5(2.7 to 6.7) | 22.5(-13.3 to 66.9) |
| American Samoa | 4(3 to 5) | 4(3 to 6) | -3.8(-29.1 to 35.7) | 68.2(48.3 to 86.3) | 48.9(37.6 to 74.7) | -28.4(-47.2 to 1.1) |
| Bermuda | 13(12 to 15) | 14(12 to 18) | 6.8(-10 to 30.7) | 77.7(69 to 87.2) | 61.3(51.5 to 76.2) | -21(-33.4 to -3.4) |
| Cook Islands | 2(1 to 2) | 2(2 to 3) | 24.8(-10.2 to 66.9) | 53.4(38.9 to 67.7) | 48.6(32.2 to 72.3) | -9(-34.5 to 21.7) |
| Greenland | 19(14 to 25) | 21(16 to 30) | 15.5(-11.9 to 48.9) | 201.9(147.3 to 267.7) | 157.5(115.6 to 217.3) | -22(-40.5 to 0.6) |
| Guam | 10(7 to 12) | 11(8 to 15) | 9.4(-17.3 to 45) | 38.5(26.2 to 46.2) | 29(21.7 to 39.5) | -24.8(-43.2 to -0.4) |
| Principality of Monaco | 10(7 to 13) | 10(8 to 14) | 8.2(-13.4 to 37) | 69.8(51.5 to 93.1) | 65.8(48.3 to 89.5) | -5.8(-24.6 to 19.3) |
| Republic of Nauru | 1(0 to 1) | 1(0 to 1) | 4.2(-18.1 to 35.7) | 101.3(58.6 to 144.3) | 91.3(53.4 to 129.5) | -9.9(-29.2 to 17.4) |
| Republic of Niue | 0(0 to 0) | 0(0 to 0) | 4.4(-15.3 to 26.7) | 69.4(47.1 to 86.3) | 64.6(44.6 to 83.1) | -7(-24.5 to 12.9) |
| Northern Mariana Islands | 3(2 to 4) | 4(3 to 7) | 12.7(-17.7 to 75.9) | 56(39.8 to 73.6) | 39.2(30.7 to 71.1) | -30(-48.9 to 9.2) |
| Republic of Palau | 1(1 to 2) | 2(1 to 3) | 41.6(3.6 to 92.5) | 50.5(33.5 to 67.8) | 46.6(29.8 to 68.2) | -7.7(-32.5 to 25.5) |
| Puerto Rico | 807(746 to 870) | 802(656 to 945) | -0.6(-19.4 to 18.2) | 80.9(74.8 to 87.2) | 68.7(56.2 to 81) | -15.1(-31.2 to 1) |
| Saint Kitts and Nevis | 10(9 to 11) | 13(10 to 15) | 25.1(1.6 to 48.2) | 132.6(121.7 to 143.2) | 97.8(80 to 116.4) | -26.2(-40.1 to -12.5) |
| Republic of San Marino | 22(17 to 30) | 17(10 to 25) | -23.4(-47.6 to 9.4) | 235.9(182.2 to 320.3) | 141.7(87.8 to 208.4) | -39.9(-58.9 to -14.2) |
| Tokelau | 0(0 to 0) | 0(0 to 0) | -7(-25.8 to 14.3) | 69.1(46.4 to 91.3) | 59.6(41.6 to 81.1) | -13.8(-31.2 to 6) |
| Tuvalu | 1(1 to 1) | 1(1 to 1) | 14.2(-7.5 to 39.5) | 68.6(46.5 to 91) | 63.3(44 to 81.9) | -7.7(-25.3 to 12.7) |
| United States Virgin Islands | 30(22 to 40) | 30(21 to 42) | 1.2(-20.3 to 34.4) | 101.8(77.1 to 137.2) | 92.8(65.9 to 130) | -8.9(-28.3 to 20.9) |
| Republic of South Sudan | 359(219 to 524) | 526(323 to 789) | 46.6(10.1 to 95.7) | 76.9(46.8 to 112.2) | 84.5(51.8 to 126.7) | 9.8(-17.5 to 46.6) |
| Republic of Sudan | 2108(1419 to 3165) | 2884(1962 to 4247) | 36.8(4.3 to 78.1) | 94(63.3 to 141.2) | 92.6(63 to 136.4) | -1.5(-24.9 to 28.2) |

* GBTC represents gallbladder and biliary tract cancer, ASR represents age-standardized rate.

1. **Section 2: Supplementary Figures**

**
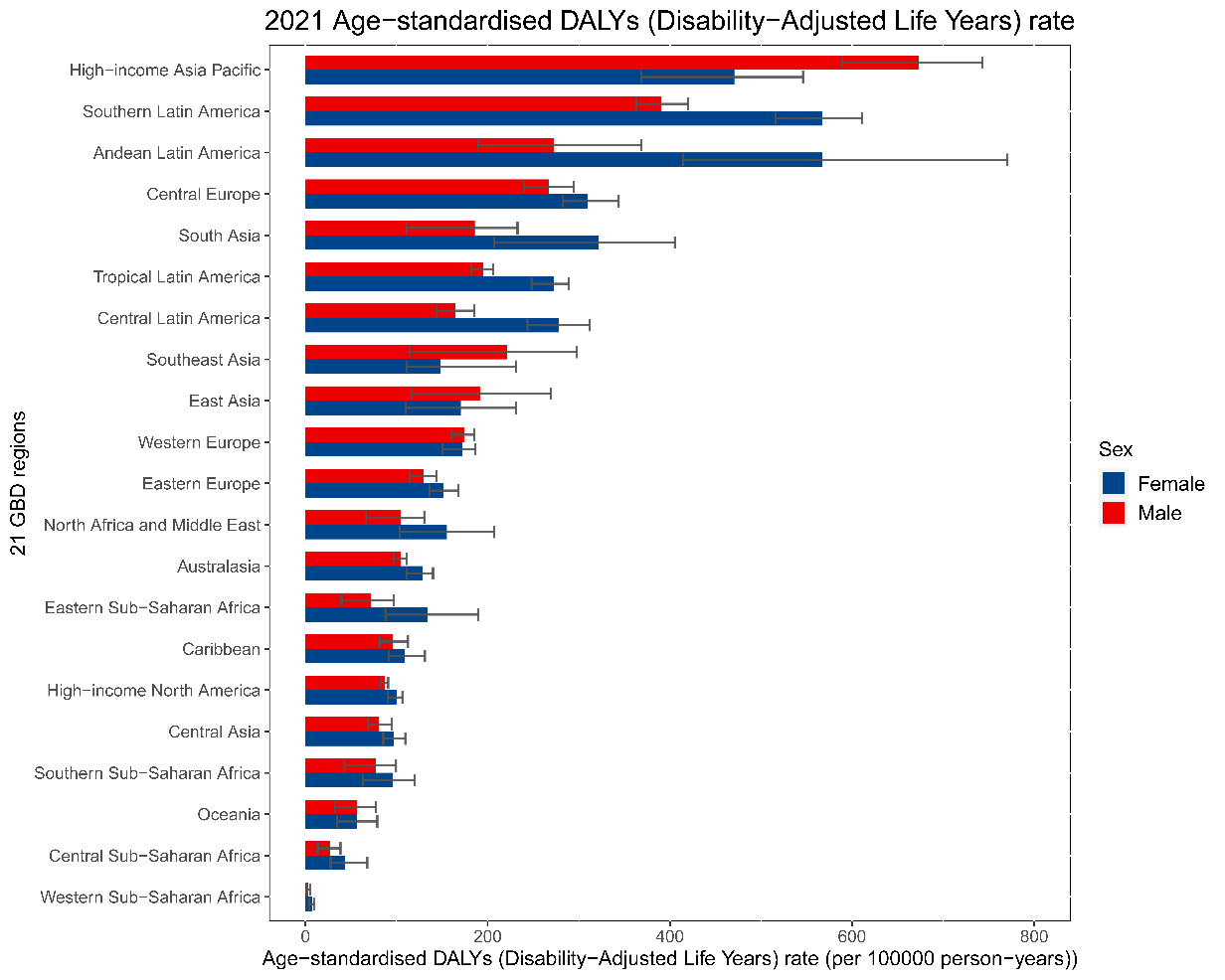
**

**Supplemental Figure 1** The ASDR of GBTC in 2021, for aged 55 years and older.

ASDR, age-standardized disability-adjusted life years rate; GBTC, gallbladder and biliary tract cancer.

**
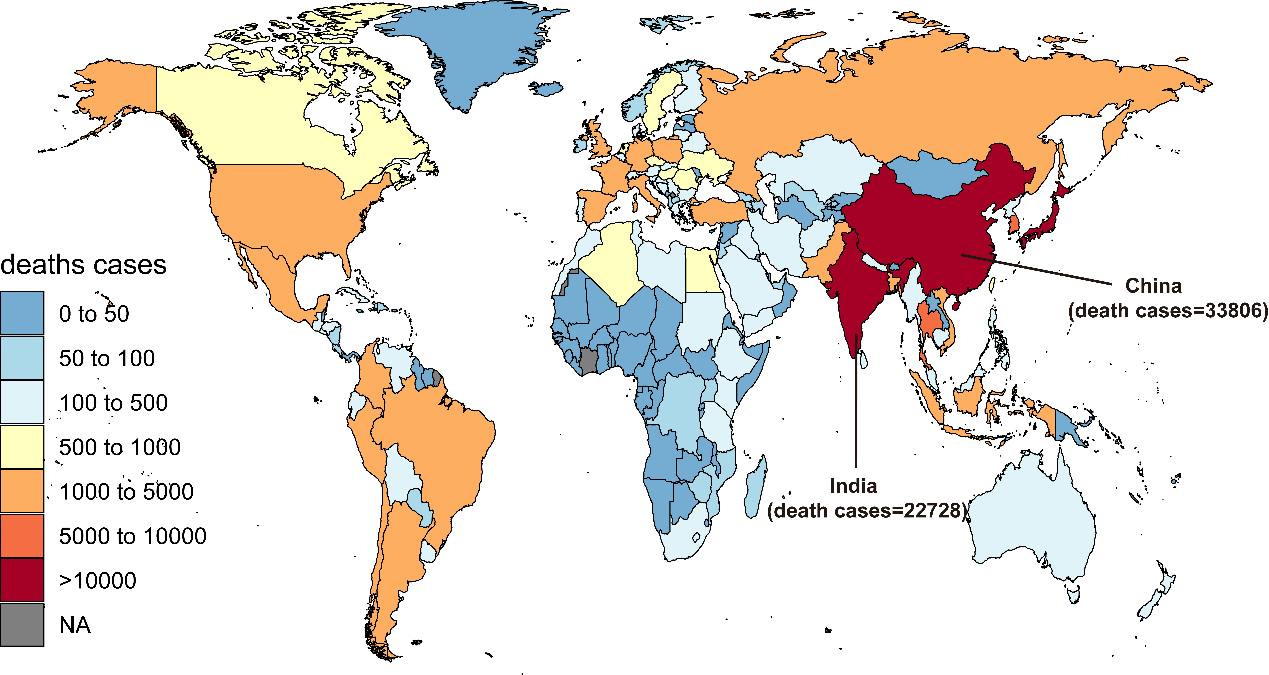
**

**Supplemental Figure 2** The death cases of GBTC burden of 204 countries and territories in 2021, for aged 55 years and older.

GBTC, gallbladder and biliary tract cancer.

**
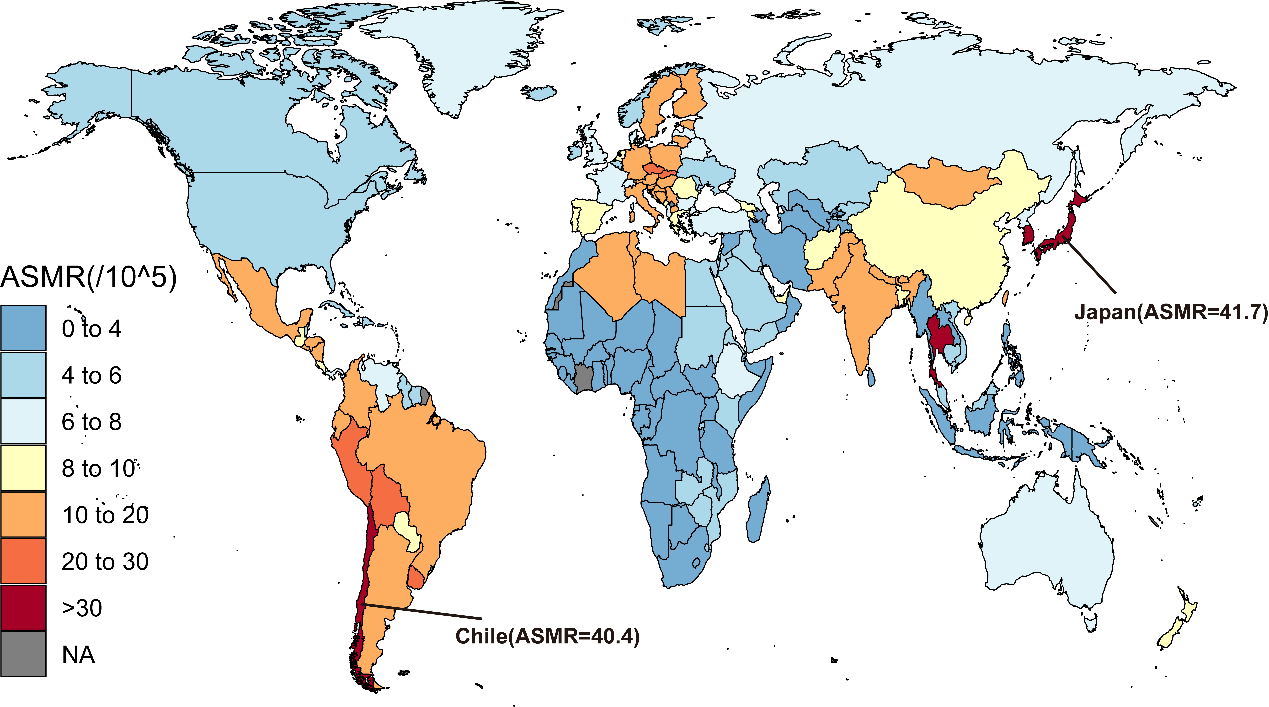
**

**Supplemental Figure 3** The ASMR of GBTC burden of 204 countries and territories in 2021 for aged 55 years and older.

ASMR, age-standardized mortality rate; GBTC, gallbladder and biliary tract cancer.

**
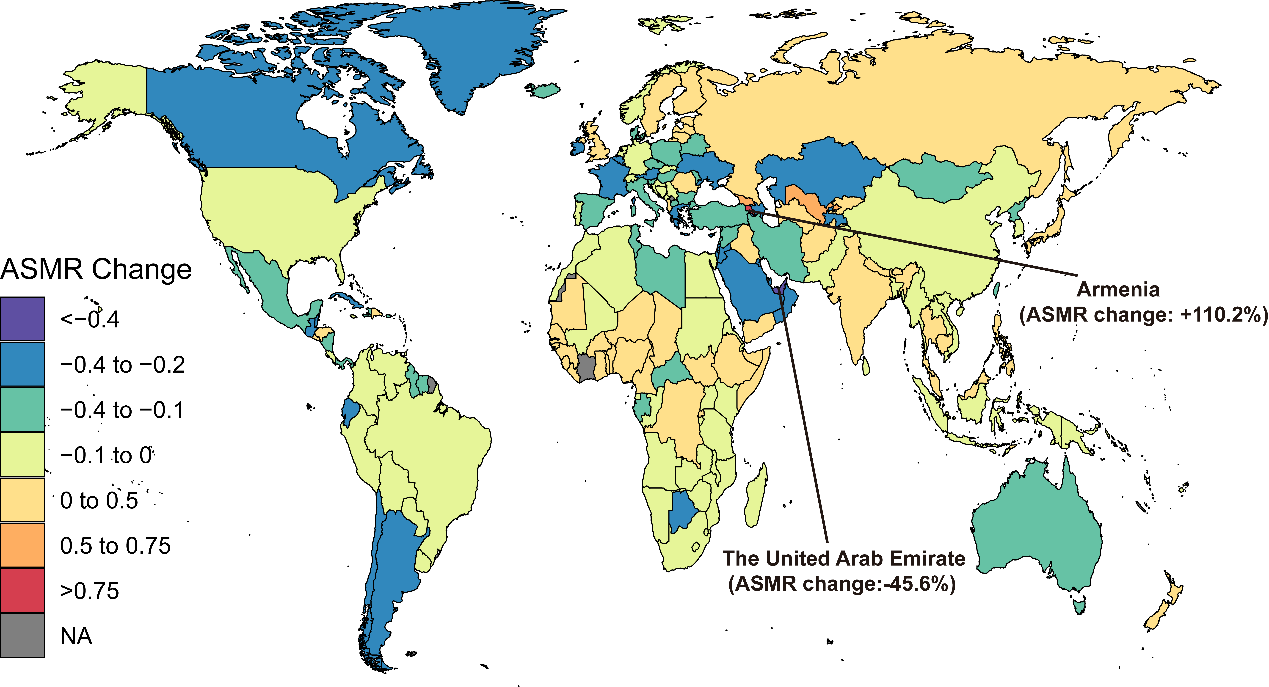
**

**Supplemental Figure 4** The ASMR percentage change of GBTC from 2010 to 2021, for aged 55 years and older.

ASMR, age-standardized mortality rate; GBTC, gallbladder and biliary tract cancer.

**
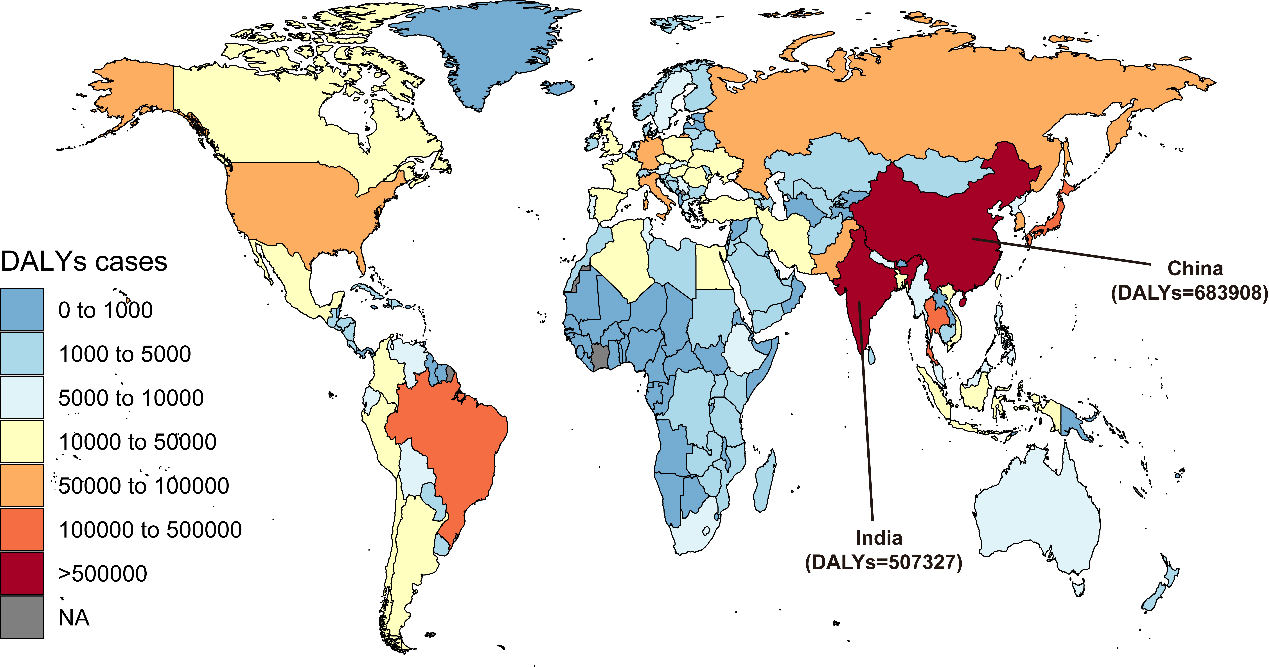
**

**Supplemental Figure 5** The DALY of GBTC burden in 2021, at the aged 55 years and older.

DALY, disability-adjusted life years; GBTC, gallbladder and biliary tract cancer.

**
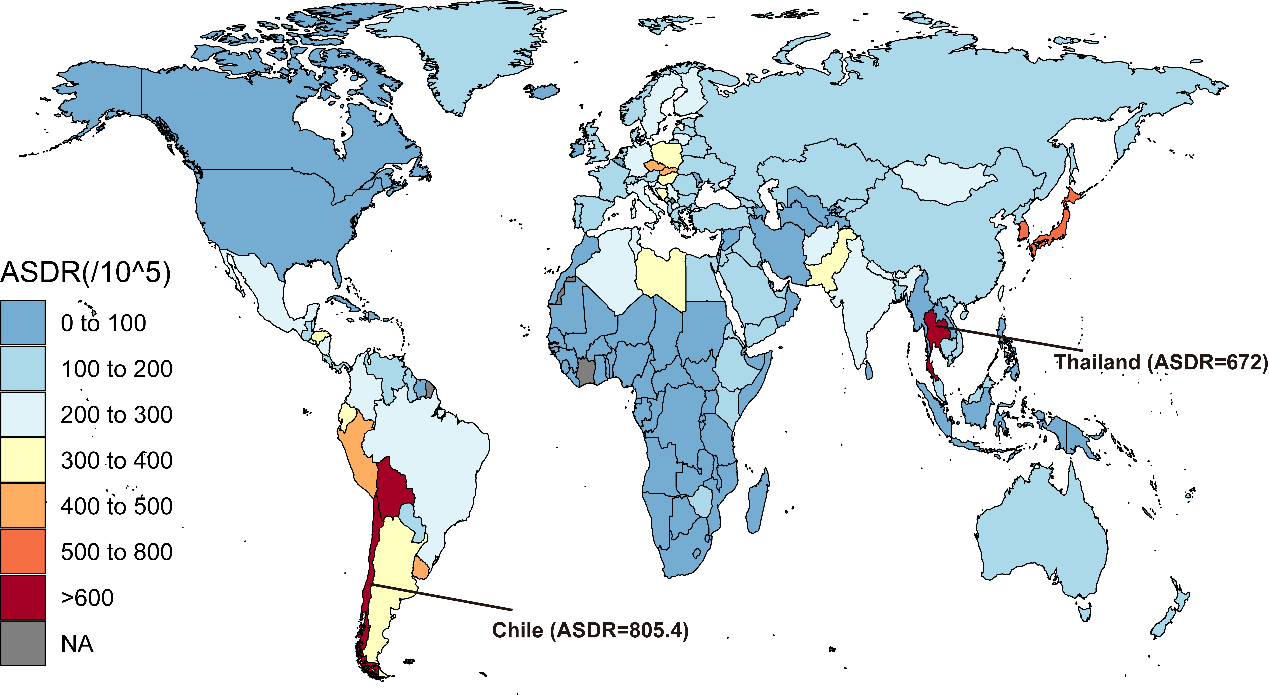
**

**Supplemental Figure 6** The ASDR of GBTC burden of 204 countries and territories in 2021 for aged 55 years and older.

ASDR, age-standardized disability-adjusted life years rate; GBTC, gallbladder and biliary tract cancer.


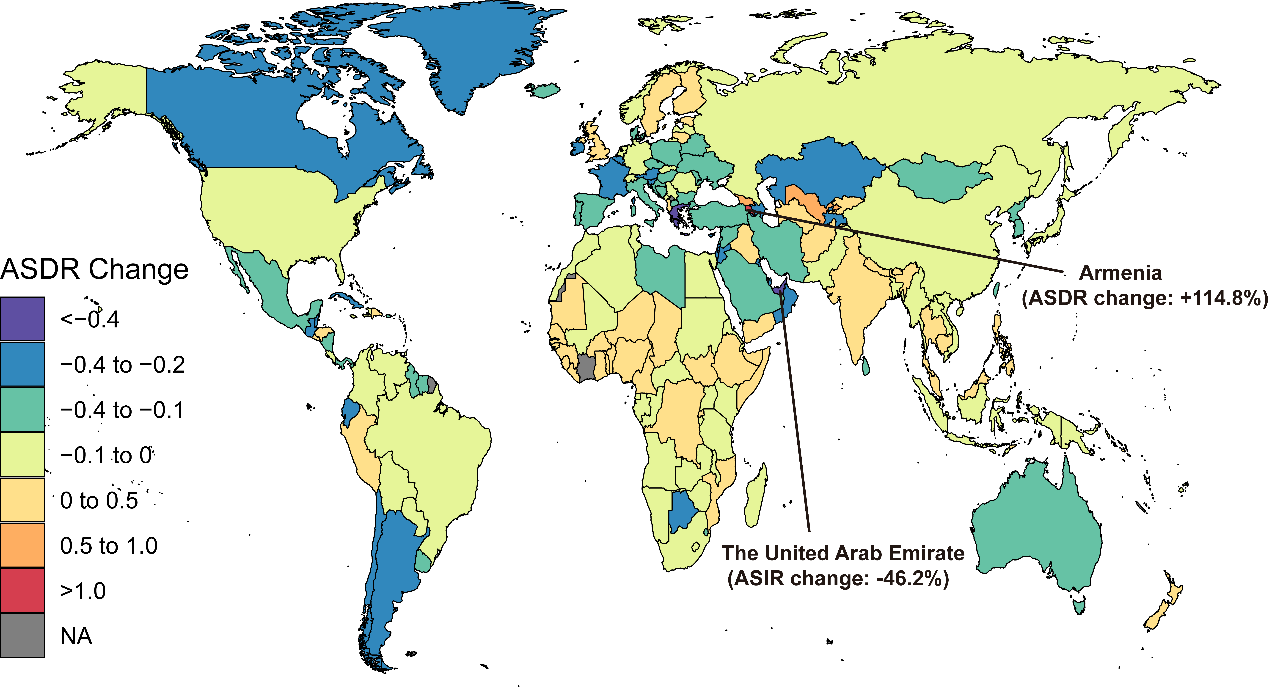


**Supplemental Figure 7** The ASDR percentage change of GBTC from 2010 to 2021 for aged 55 years and older.

ASDR, age-standardized disability-adjusted life years rate; GBTC, gallbladder and biliary tract cancer.


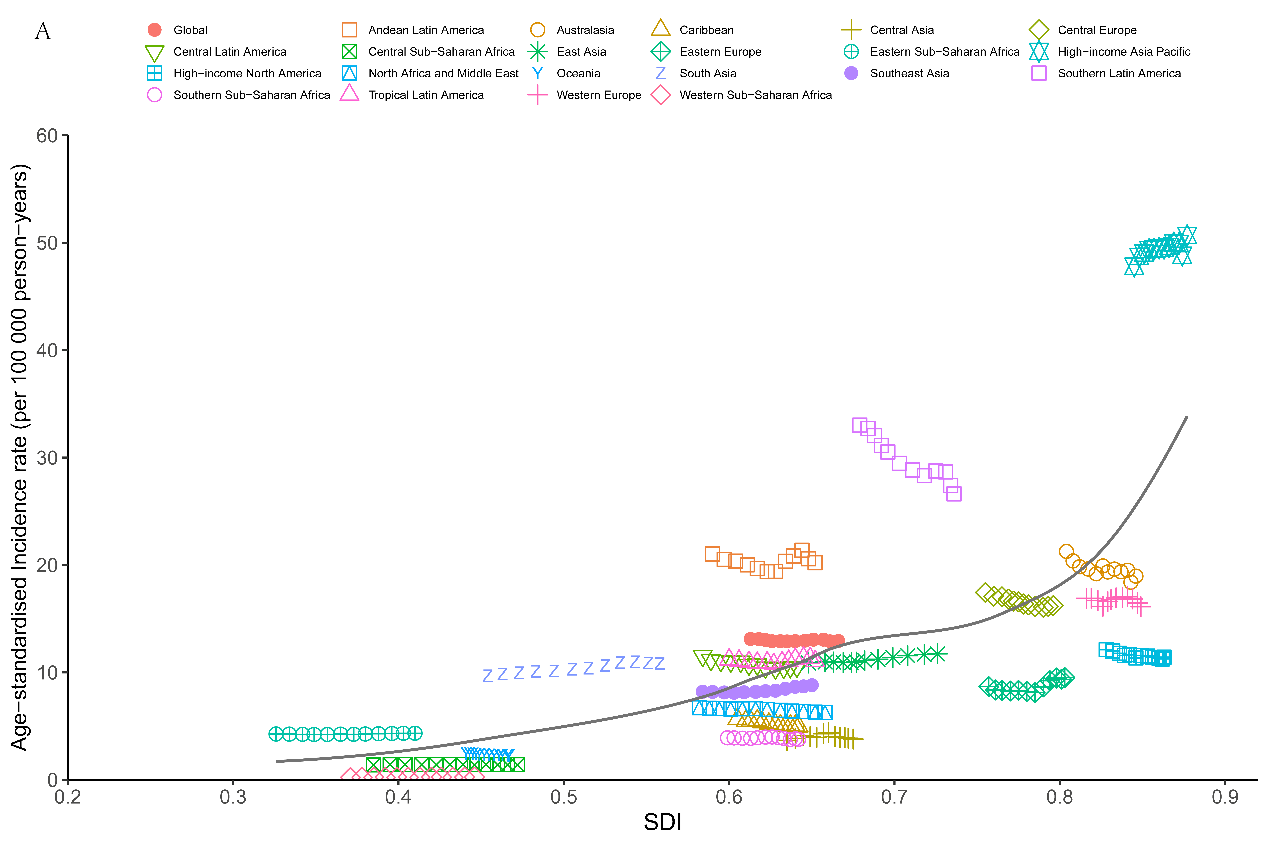


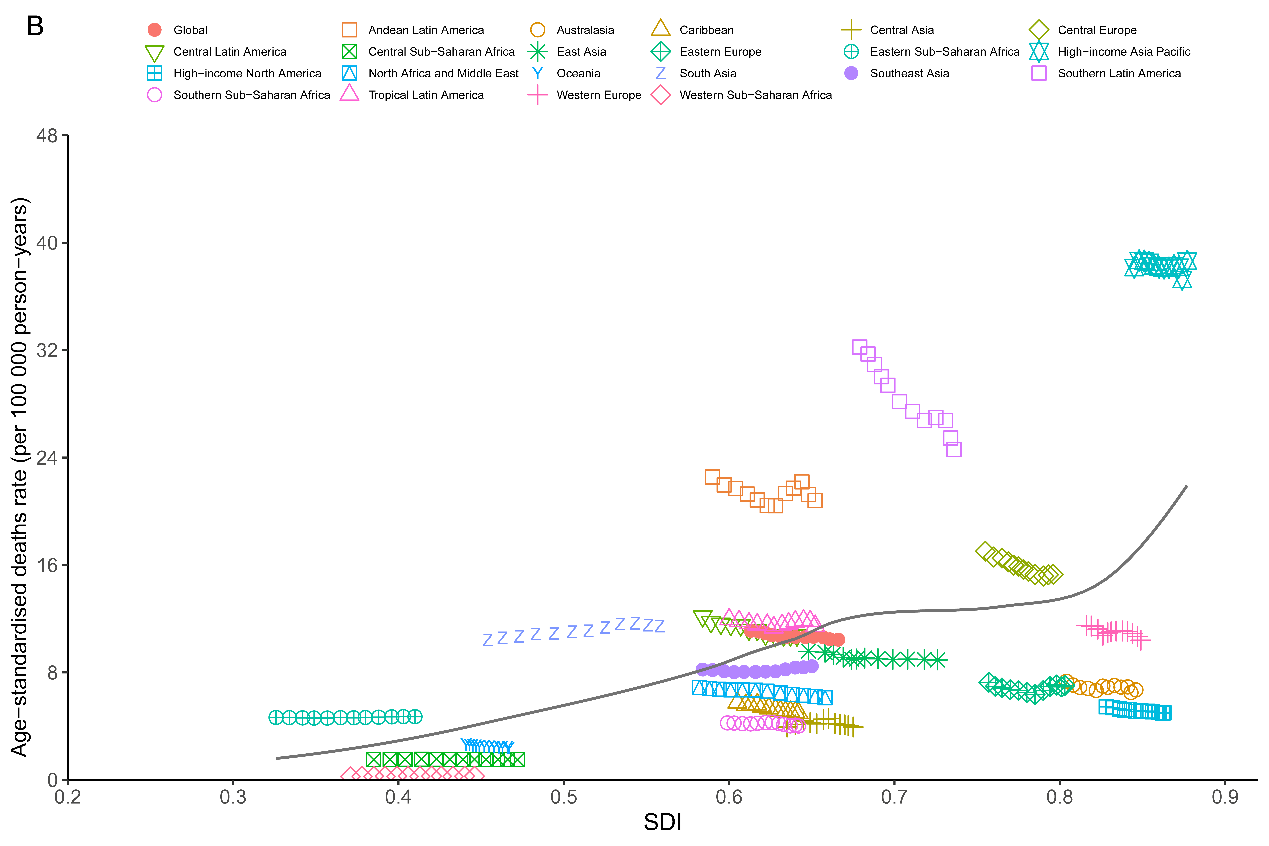


**Supplemental Figure 8** The trend in (A) ASIR and (B) ASMR in aged 55 years and older of GBTC across 21 GBD regions by SDI for both sexes, from 2010 to 2021. Expected values are shown as the black line.

ASIR, age-standardized incidence rate; ASMR, age-standardized mortality rate; GBTC, gallbladder and biliary tract cancer; SDI, Socio-demographic Index.

**
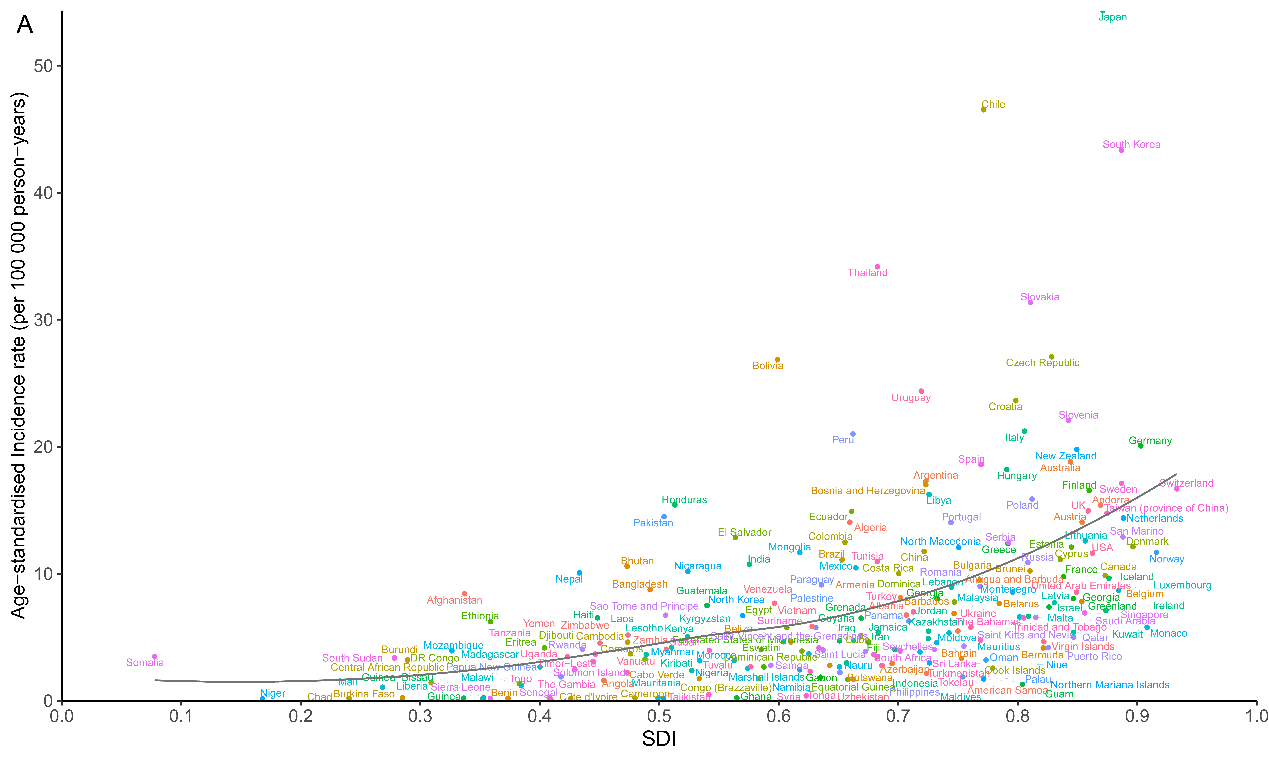
**

**
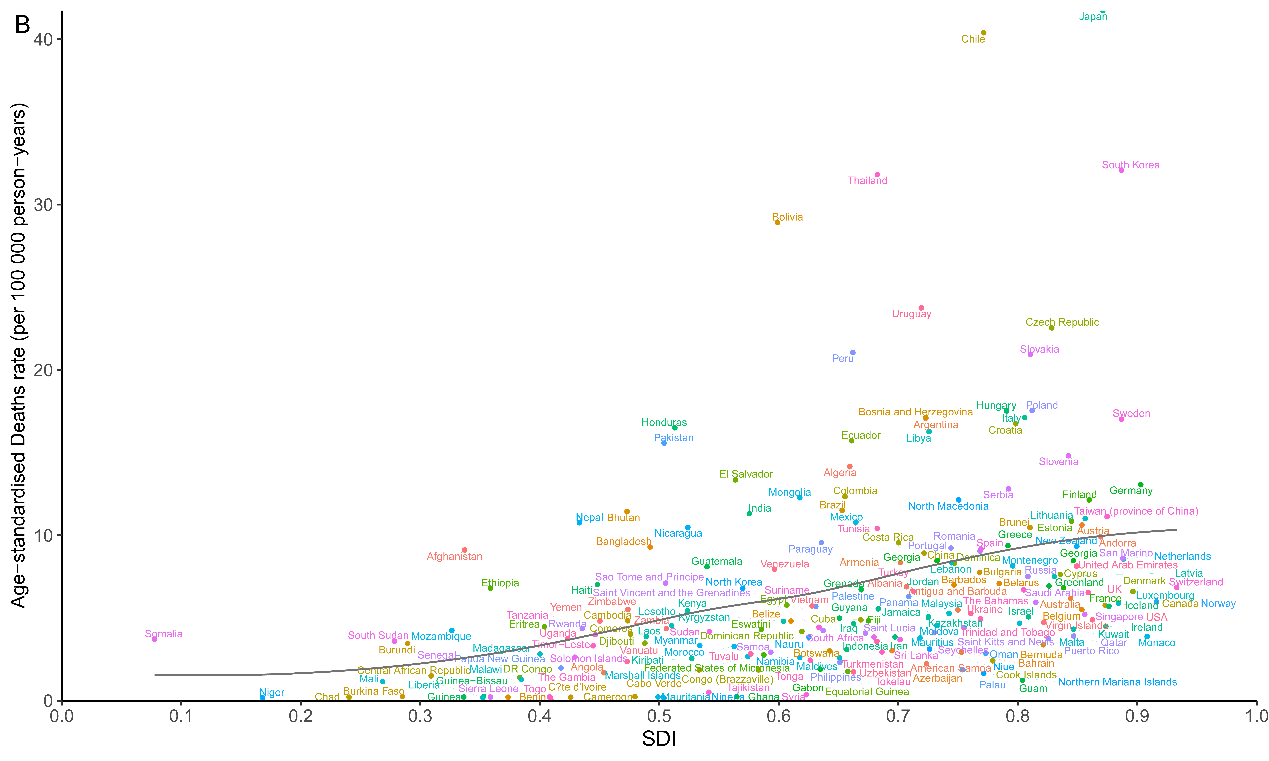
**

**Supplemental Figure 9** The trend in (A) ASIR and (B) ASMR in aged 55 years and older of GBTC across 204 countries and territories by SDI for both sexes, from 2010 to 2021. Expected values are shown as the black line.

ASIR, age-standardized incidence rate; ASMR, age-standardized mortality rate; GBTC, gallbladder and biliary tract cancer; SDI, Socio-demographic Index.

**
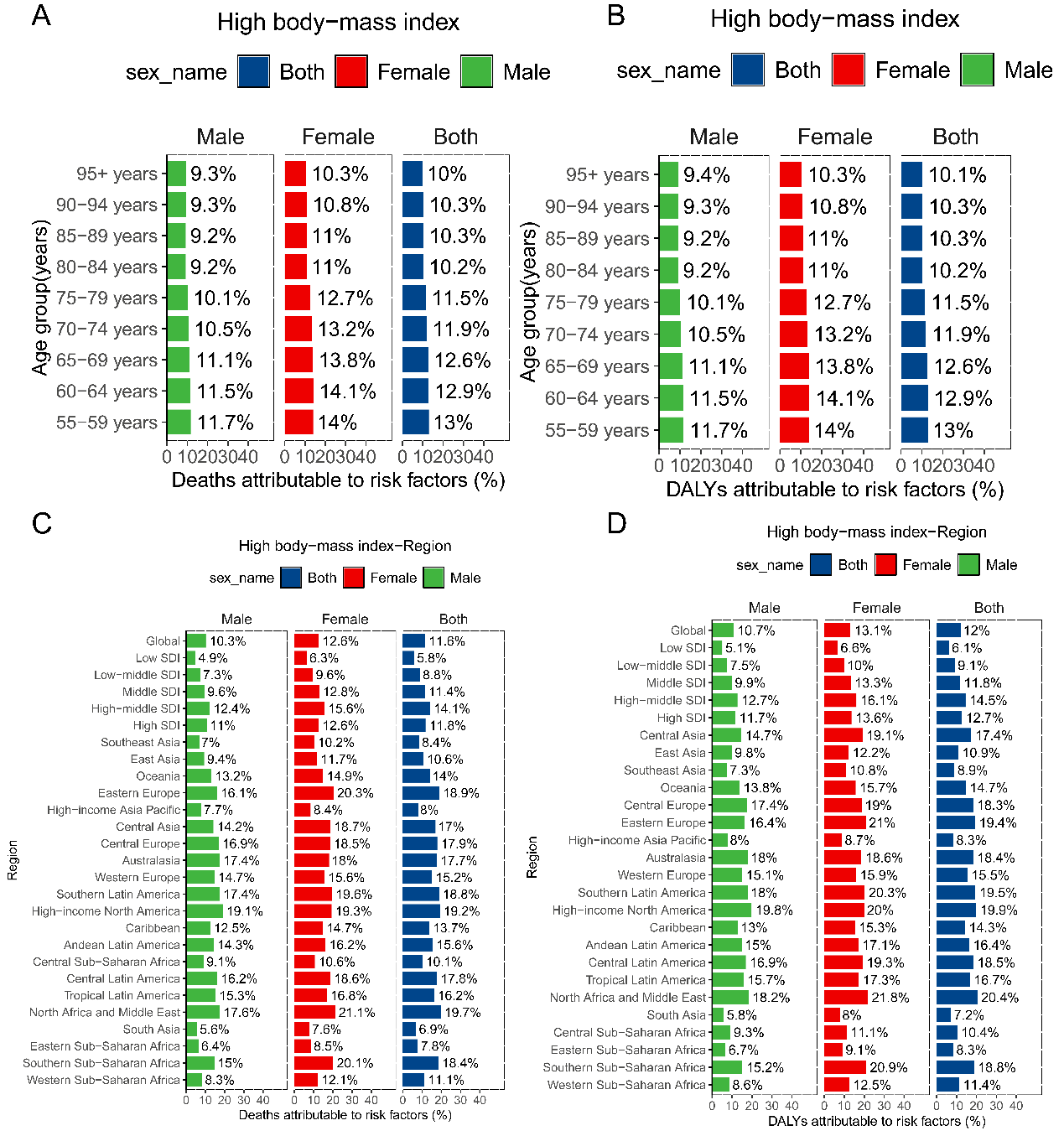
**

**Supplemental Figure 10** The distribution of GBTC risk factor in sex, regions, and aged 55 years and older.

(A)Deaths and (B) DALYs distribution caused by risk factor, both sex and age group. (C) Deaths and (D) DALYs distribution of GBTC risk factor and sex in global, 5 SDI areas, and 21 regions at the aged 55 years and older.

GBTC, gallbladder and biliary tract cancer; DALYs, disability-adjusted life years; SDI, Socio-demographic Index.
